# Materials–Structure–Hardware–Algorithm Co-Driven Hierarchical Optimization for Flexible Sensors

**DOI:** 10.1007/s40820-026-02274-w

**Published:** 2026-08-10

**Authors:** Pengyu Zhu, Peng He, Jinbo Pang, Mark Hermann Rummeli, Hong Liu, Weijia Zhou, Rafael Gregorio Mendes, Shuye Zhang

**Affiliations:** 1 State Key Laboratory of Precision Welding & Joining of Materials and Structures, School of Materials Science and Engineering, Harbin Institute of Technology, 92 Xidazhi Street, Nangang District, Harbin, 150001 People’s Republic of China; 2 Shandong Key Laboratory of Functional Materials for Integrated Lithium Niobate Photonics, Institute for Advanced Interdisciplinary Research (iAIR), University of Jinan, 336 Nanxinzhuang West Road, Jinan, 250022 People’s Republic of China; 3 Chongqing Research Institute, Harbin Institute of Technology, Chongqing, 401135 People’s Republic of China; 4 Soochow Institute for Energy and Materials Innovations, College of Energy, Key Laboratory of Advanced Carbon Materials and Wearable Energy Technologies of Jiangsu Province, Key Laboratory of Core Technology of High Specific Energy Battery and Key Materials for Petroleum and Chemical Industry, Soochow University, Suzhou, 215006 People’s Republic of China; 5 Electron Beam Emergent Additive Manufacturing (EBEAM) Centre, Institute of Environmental Technology (IET), Centre for Energy and Environmental Technologies (CEET), VSB—Technical University of Ostrava, 17. Listopadu 15, 708 33 Ostrava, Czech Republic; 6 Institute for Materials Chemistry, Leibniz Institute for Solid State and Materials Research Dresden (IFW Dresden), 20 Helmholtz Strasse, 01069 Dresden, Germany; 7 State Key Laboratory of Crystal Materials, Shandong University, 27 Shanda South Road, Jinan, 250100 People’s Republic of China; 8 Interfaces, Confinement, Matériaux Et Nanostructures, CNRS-Orléans, UMR7374, 1b rue de la Férollerie—CS40059, 45071 Orléans, France

**Keywords:** Flexible sensors, Multimodal sensors, In-sensor computing, Sensing algorithms, Human–machine interfaces

## Abstract

Flexible electronics represent a paradigm shift in modern electronics, with flexible sensors serving as pivotal components in these systems. Despite significant advances driven by innovations in materials, structures, hardware, and algorithms, conventional design approaches that focus on optimizing individual hierarchies have inherent performance trade-offs, limiting further development. This review contends that future performance enhancements can no longer rely solely on breakthroughs in separate components. Still, it must adopt a new co-design paradigm spanning the “materials–structure–hardware–algorithm” hierarchy. In this review, we systematically organized the research landscape and representative advances across these four key hierarchies, analyzed the importance and recent breakthroughs in hierarchical synergy, and established a forward-looking theoretical framework to foster innovation and development in the field of flexible sensing.

## Highlights

- A novel co-design paradigm is proposed. A holistic co-design paradigm of “materials–structure–hardware–algorithm” is proposed here to overcome the performance trade-offs of traditional fragmented sensors.
- A systematic research framework is established based on the fundamental composition of flexible sensors. The interrelations within this dynamic field are clarified by structuring the review on the core hierarchies of materials, structure, hardware, and algorithms.
- The research landscape across the field’s core components is systematically organized. The review provides a comprehensive overview of representative advances at the material, structural, hardware, and algorithmic hierarchies, and outlines future challenges and directions for next-generation flexible sensors.

## Graphical abstract

**Figure:**
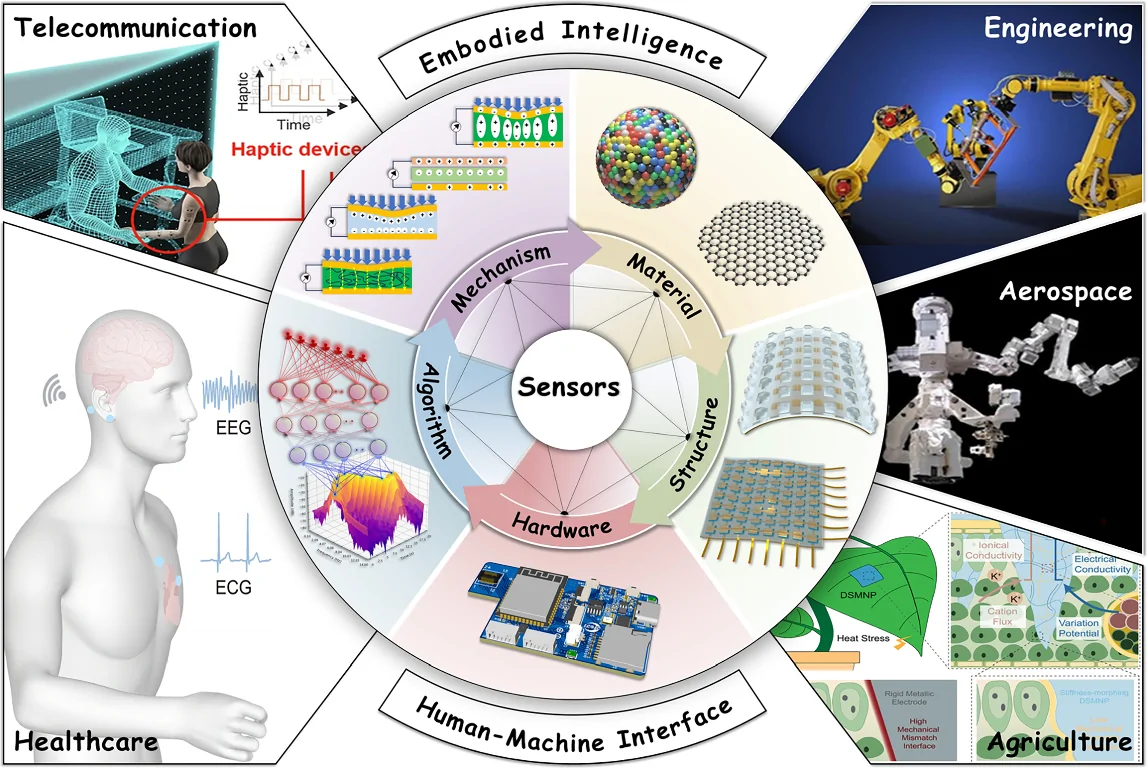

## Introduction

Flexible electronics represent a paradigm shift in modern electronics. These electronics are driving transformative progress across fields, such as healthcare, robotics, and human–computer interactions, leveraging advancements in microelectronics, e.g., the Internet of Things (IoT), mechatronics, materials science, and chemistry [1, 2]. In this shift, flexible sensors and sensing devices have emerged as pivotal components because they not only adopt flexible materials and deformable structural designs, but also maintain stable sensing performance under mechanical deformations such as bending, stretching, and surface conformal contact.

Flexible sensors have higher adaptability than their conventional rigid counterparts [3], thus enabling seamless integration with irregular, complex curvilinear surfaces such as mechanical components, human skin, and textiles. These advanced devices can be categorized into the following main types according to the target stimuli, such as flexible pressure sensors, strain sensors, gas sensors, and humidity sensors [4–6]. These diverse functionalities, however, can only be realized with their essential “soft” components, typically including flexible structural substrates (e.g., elastomers and polymers) and soft active sensing elements (e.g., functionalized nanomaterials, conductive hydrogels, and liquid metals). These soft components have unique advantages because of their defining characteristics, such as exceptional mechanical stretchability, biocompatibility, and stable electrical performance under dynamic strain, which precisely satisfy the increasing demand for sensing technologies in new conditions such as wearable devices, embodied intelligence, smart healthcare [7–9], smart homes [10], intelligent machinery [11], and smart agriculture [12, 13].

In recent years, flexible sensors have experienced remarkable progress as follows. The first is in material innovation, where various high-performance materials and fabrication techniques have emerged, including novel 2D materials with high conductivity and tunable properties [14–16], highly conductive and stable gel materials [17–19], high-precision 3D printing technologies [20, 21], self-healing materials [22–24], and recyclable/biodegradable materials [25–27], which are integrated and collectively enhance sensor performance. The second is structural engineering, which further enhances the capabilities of these materials. For example, interlocked micro-dome array structures significantly boost sensitivity [28] and further improve the detection limit to 0.2 Pa [29]. The third is efficient hardware and circuit design, which allows for not only the applications of these advancements, but also reduced system response latency to the millisecond level and minimized power consumption [30]. The fourth is advanced algorithms, such as the Quasi-Recurrent Neural Network (QRNN), which enable real-time, multi-parameter, and multimodal signal analysis, and therefore ensure high accuracy and allow for high-dimensional applications [31].

Fundamental challenges still remain, however, which limit the further enhancement of the performance. First of all, it is known that a complete flexible sensing system works on the underlying physical mechanisms and involves sequential design of a multi-level hierarchy composed of materials, structure, hardware, and algorithms. In these sequential stages, an integrated signal-perception chain is formed and each level can influence the final performance. In traditional designs, however, usually only a single hierarchy is optimized, no matter in material innovation or in structural engineering, without the consideration of synergistic co-design across materials, structures, hardware, and algorithms. This is a fragmented methodology, which inevitably induce performance trade-offs in conventional flexible sensors, i.e., high sensitivity can only be achieved at a constrained detection range [32]. In addition, excellent flexibility is usually achieved at the expense of good stability [33], and multimodal integration is often achieved at the expense of high performance in individual modalities.

**Fig. 1:**
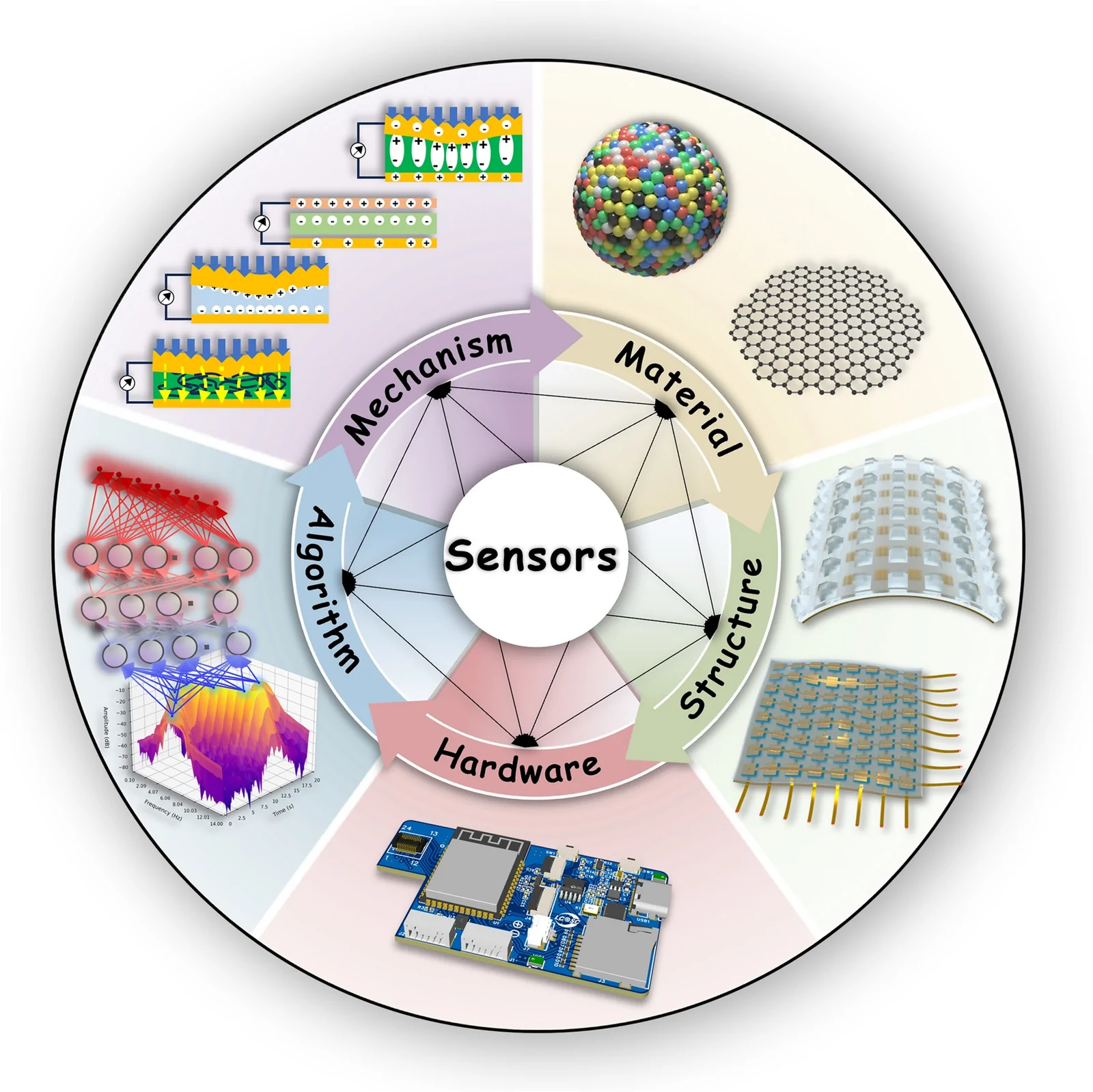
Schematic of holistic co-design. Sensing mechanisms, Material, Structure, Hardware, Algorithms

With the rapid evolution in this field, the performance of sensors cannot be enhanced solely through breakthroughs in individual components. Instead, holistic co-design is needed which involves material selection, structural design, hardware implementation, and algorithmic optimization, as illustrated in Fig. 1. Considering this background, the co-design paradigm of “materials–structure–hardware–algorithm” has emerged as a critical pathway to overcome the existing bottlenecks in performance and application. This co-design paradigm systematically coordinates decisions among multiple levels of the hierarchy, thereby enabling hierarchical optimization and the consequent global system-level optimization of flexible sensors. This paradigm also enables a leap forward in overall sensor performance by exploiting cross-level interactions and trade-offs to realize the potential of each component. Compared with traditional research approaches, the above co-design paradigm overcomes the inherent limitations of fragmented design through system-level optimization, enabling the release of “system-level performance gains” beyond the scope of single-point optimization, and represents a promising direction for advancing the field of flexible electronics.

While existing reviews provide comprehensive overviews of the field [34–39], systematic organization and analysis across multiple hierarchies, from underlying materials to top-level algorithms, remain underexplored. This gap has hindered the refinement of the theoretical framework and limited the expansion of application boundaries. With the rapid development of frontier applications such as human–computer interaction, embodied intelligence, and brain–computer interfaces, the demand for flexible sensors continues to grow. At this critical juncture, a deep understanding and systematic integration of co-design principles are vital for developing and deploying next-generation flexible sensors.

In this review, we systematically organize the research landscape and representative advances in flexible sensors across the four key hierarchies of “materials–structure–hardware–algorithm”, and conceptualize this emerging methodology of hierarchical synergy while analyzing its importance and recent breakthroughs. Finally, we summarize the key challenges facing the field and outline critical future directions. This review aims to establish a forward-looking theoretical framework to enrich researchers’ thinking, support groundbreaking research and foster innovative development in flexible sensors.

As a narrative review focused on introducing the novel “materials–structure–hardware–algorithm” co-design paradigm, this article selectively cites representative works that exemplify key innovations at each level of the framework, prioritizing studies with high impact or strong conceptual relevance to the proposed paradigm. Efforts were made to ensure balanced coverage across different material systems, sensing modalities, and application areas.

## Sensing Mechanisms

**Fig. 2:**
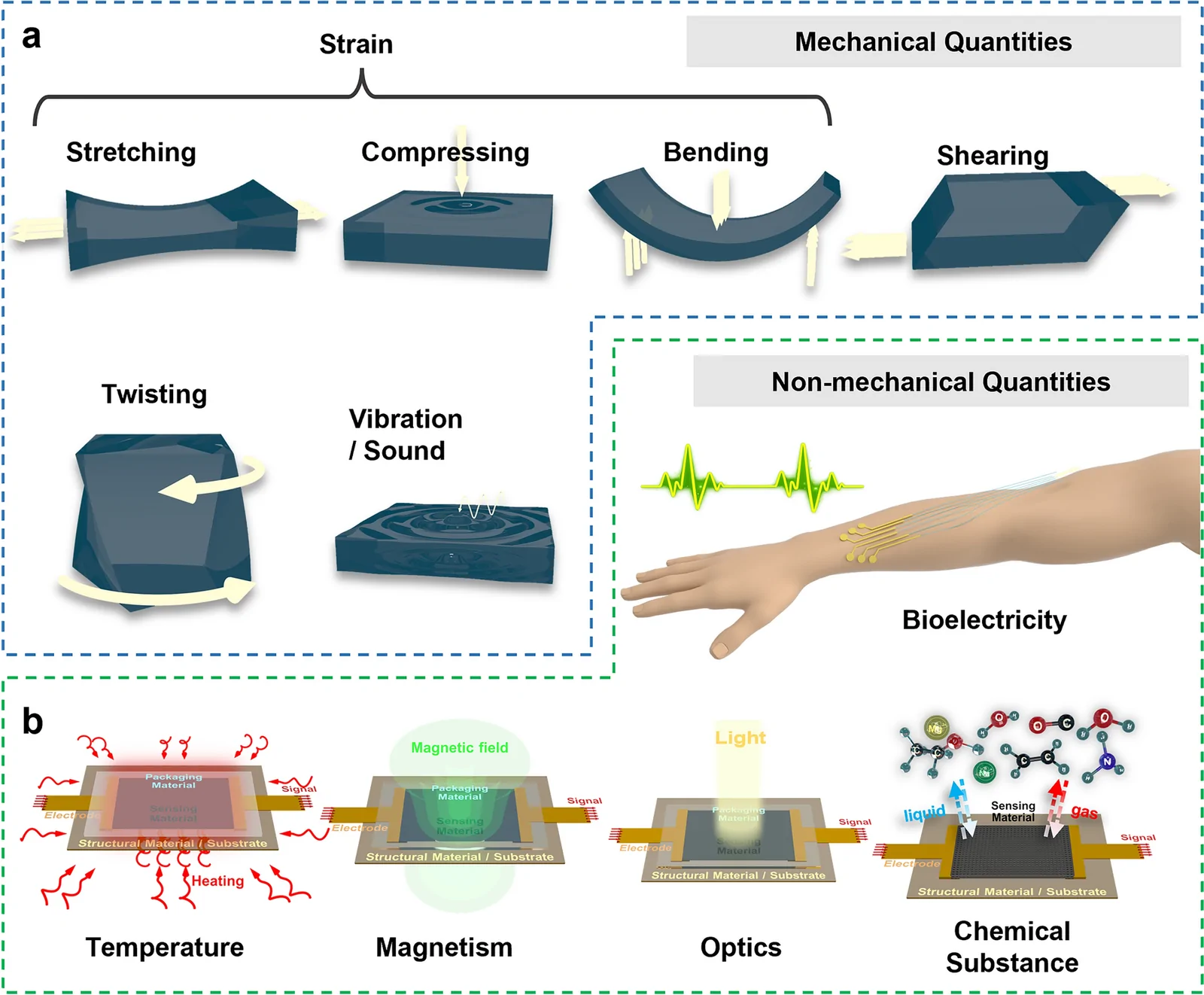
Sensing quantities. **a** Mechanical quantities. **b** Non-mechanical quantities

The physical/chemical characteristics of sensing objects directly dictate sensor design paradigms, particularly the sensing mechanism, material selection, and device architecture. Currently, sensing objects covered by sensor technology can be primarily categorized into two classes, as illustrated in Fig. 2:

- 1. Mechanical quantities and induced deformation include static loads such as stretch, compression [40], bending [41] (also often classified as *strain*), shear [42], and twist [43], as well as dynamic responses such as vibrations [44] and acoustic waves [45], along with the resulting spatial deformation fields (e.g., force/strain distribution, shape, and posture).
- 2. Non-mechanical quantities refer to physical or chemical information in the environment, encompassing physical signals such as bioelectricity [46, 47], temperature [48, 49], optics [50], and magnetism [51], as well as chemical signals including the concentration of specific molecules in ambient gases or liquids (e.g., water [52], ethanol [53], purines [53], carbon dioxide [54], bacteria [54], nitrogen dioxide [55–57], lactic acid [58–60], acetic acid [61], ascorbic acid [62], dopamine [62], uric acid [53, 59, 63], amino acids [59, 63], vitamins [63], oxygen [64] and glucose [60, 62, 63]).

Notably, in some cases, the detection of the aforementioned non-mechanical quantities can be essentially achieved indirectly through mechanical deformation as an intermediary mechanism, e.g., from temperature variation, to mechanical deformation, and to deformation sensing [48]; from humidity variation, to mechanical deformation, and to deformation sensing; and from magnetic field, to magnetostriction, and to deformation sensing [65].

### Fundamental Mechanisms

**Fig. 3:**
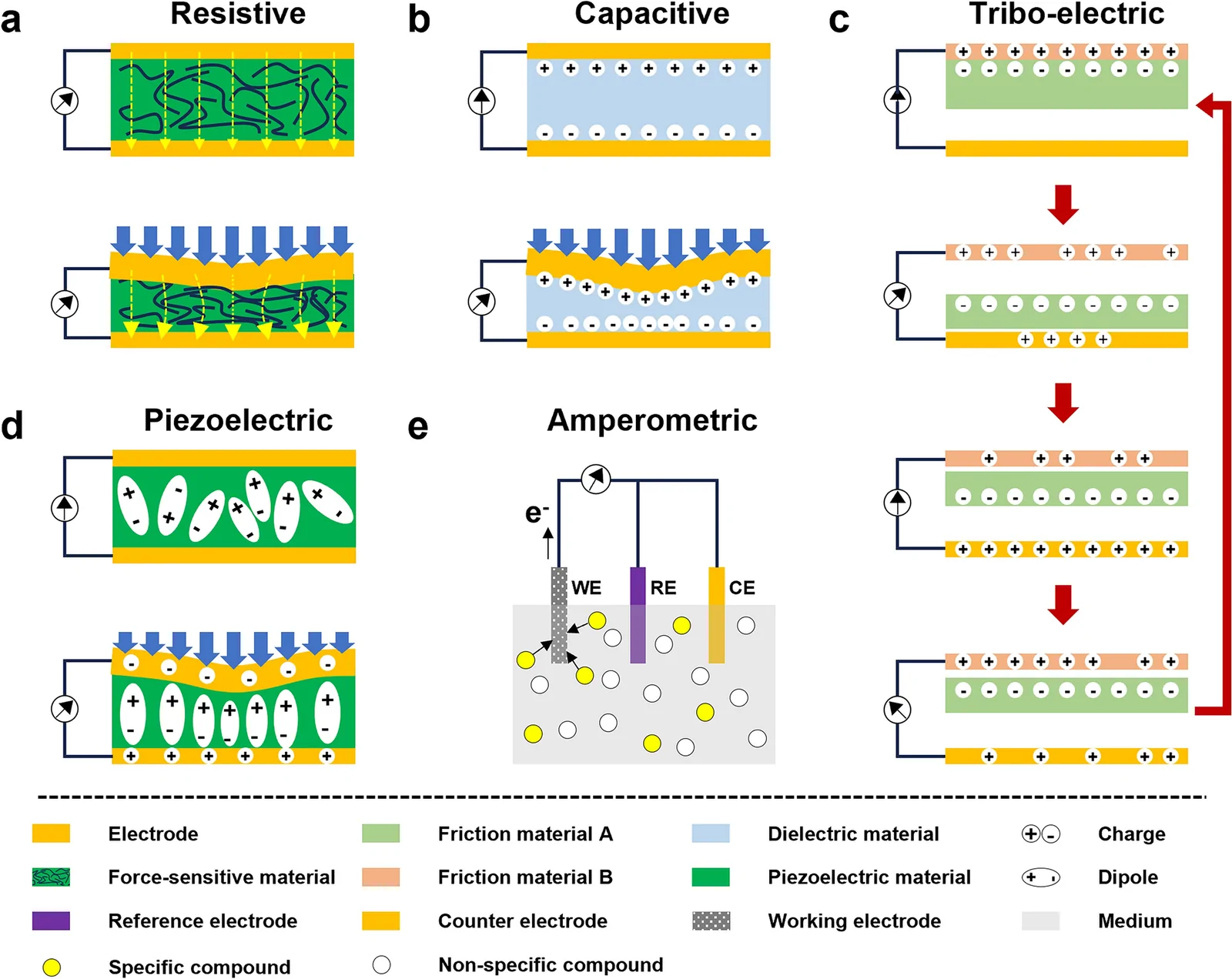
Fundamental sensing mechanisms: **a** Resistive, **b** Capacitive, **c** Triboelectric, **d** Piezoelectric, and **e** Amperometric

The fundamental sensing mechanism serves as the cornerstone of the overall sensor system, influencing all subsequent designs of materials, structures, hardware circuits, and algorithms. The primary sensing mechanisms include resistive, capacitive, triboelectric, piezoelectric, and amperometric modes, as shown in Fig. 3.

#### Resistive Sensors

Resistive sensors operate by establishing a mapping relationship between external stimuli and the electrical resistance of the sensing material. The fundamental mechanism relies on changes in the core parameters governing a material’s resistivity, including the charge-carrier status (e.g., concentration and mobility) [66], the conductive distribution, and the interfacial contact conditions of conductive materials under external stimulation [40]. These changes subsequently induce a measurable change in the material’s overall electrical resistance. The basic electrical resistance formula is given by **Eq. **(1):

1 $$R = \rho \cdot \frac{L}{A}$$

where ***ρ*** denotes the resistivity of the material, ***L*** represents its length, and ***A*** is the cross-sectional area.

A basic resistive sensor assembly typically consists of a sensing material, two electrodes, and a necessary supporting substrate. The structural simplicity of this configuration results in exceptional versatility and lower cost than other modes [11], making resistive sensing the most widely used methodology. This mechanism can be used to detect diverse stimuli such as stretch [67, 68], compression [69–73], bending [74], shear [75, 76], twisting [75], vibrations [69], temperature [77, 78], humidity [49, 77], bioelectricity [47, 79], chemical species [55], and sound [69].

#### Capacitive Sensors

A capacitive sensor is a device that detects external stimuli through changes in capacitance, with its operating principle based on the parallel-plate capacitor model, as described by **Eq. **(2):

2 $$C = \frac{{\varepsilon_{0} \cdot \varepsilon_{r} \cdot A}}{d}$$

where ***ε0*** is the vacuum permittivity, ***εr*** is the relative permittivity of the dielectric material, ***A*** is the effective overlapping area of the electrodes, and ***d*** is the separation distance between the electrodes. This equation demonstrates that changes in capacitance, and its enabled sensing, can be measured by external stimuli induced variations in the inter-electrode distance ***d***, the relative permittivity ***εr***, or overlapping area ***A***.

The parameters ***d*** and ***A*** are typically affected by mechanical quantities, and the parameter ***ε***_***r***_ is primarily affected by non-mechanical quantities. Therefore, capacitive sensors can detect a wide range of signals, including stretch [80, 81], compression [82–87], bending [84, 88], shear [87], twist [88], vibration [44, 86], and humidity [89, 90].

Capacitive sensors have these advantages: a wide detection range, broad linear response region [91, 92], fast dynamic response, and low-power consumption. They can be used in non-contact proximity sensing [84, 90]. However, their interference immunity are relatively poor and they are susceptible to environmental disturbances, e.g., variations in temperature, humidity, or electromagnetic fields, which consequently influence the properties of the electrodes or the dielectric material [84].

#### Triboelectric Sensors

A triboelectric sensor is a device based on the principles of both triboelectric effect and electrostatic induction to transduce mechanical stimuli into measurable electrical signals when two dissimilar materials come into contact and subsequently separate. In this process, electrons transfer from one material to the other due to a difference in their electron affinities, thus resulting in positive charges on the donor surface and negative charges on the acceptor surface. The magnitude of the transferred charge is determined by the properties, contact area, and separation kinetics of the materials. When these electrostatically charged surfaces approach a conductor, the induced electric field drives the redistribution of free charges in the conductor via electrostatic induction, thus generating a measurable electrical output [49].

A typical triboelectric sensor comprises a pair of triboelectric layers with distinct electron affinities, electrodes for charge collection, and a deformable support structure that provides a restoring force.

Triboelectric sensors offer several advantages, such as self-powering [93–95], high sensitivity, and broad material compatibility [96, 97]. They can also detect a diverse range of signals, including stretch [98, 99], compressive [100] and bending strain [100], shear [82], humidity [49], temperature [49], and vibration suitability [93]. A principal drawback, however, is their long-term stability, which can be compromised by charge decay caused by material wear [94].

#### Piezoelectric Sensors

Piezoelectric sensors operate on the principle of the direct piezoelectric effect to detect signals, focusing on measurable electrical signals generated by piezoelectric materials upon deformation. When a piezoelectric material is subjected to an external force and undergoes mechanical strain, the internal dipoles within the material become polarized and reorient. This results in the accumulation of equal-magnitude but opposite-polarity charges on the material’s surface, including a potential difference between the electrodes, and generating an electrical signal in the circuit. After unloading the external force, the electric dipoles return to their original positions, restoring the sensor to an electrically neutral state [101].

Its structure is also analogous to that of resistive sensors, with the most fundamental piezoelectric sensor requiring only a fragment of piezoelectric material and a pair of electrodes [101, 102].

The fundamental principle of the direct piezoelectric effect makes it especially suitable for pressure sensing [101–103]. Additional advantages include self-powering capabilities [101, 102], favorable dynamic characteristics [104], and a broad frequency bandwidth. Other applicable measurands encompass shear [42], torque [43], acoustic signals [45], and vibrations [104]. However, the electric charge generated by the piezoelectric effect tends to leak gradually within the circuit. It cannot be maintained over extended periods, rendering these sensors unsuitable for static measurements.

#### Amperometric Sensors

Amperometric sensors have significant advantages in chemical substances sensing scenarios, which accomplish quantitative sensing by measuring the electric current generated at the interface between a medium (liquid or gas) and the working electrode under potentiostatic conditions. The electric current is induced during the oxidation or reduction reactions of the target compounds at the interfacial region.

This process can be explained by the principle of Faraday’s laws of electrolysis. The magnitude of the Faradaic current sustained by continuous electron transfer at the working electrode surface in specific compounds in the medium is directly proportional to their concentration under a fixed applied potential and conditions of stable mass transport. Amperometric sensors are the most extensively applied type among all electrochemical sensors.

An amperometric sensor typically has three electrodes: a working electrode (WE), a reference electrode (RE), and a counter electrode (CE). The working electrode is used to drive redox reactions under a constant potential; the reference electrode is used as a stable potential reference; and the counter electrode is used to pass the current to maintain the circuit required by the working electrode. However, the working electrode has to be modified to prevent its response to multiple compounds and the consequent impair of its anti-interference capability. Scholars often modify the working electrode with enzymes (enzymatic amperometric detection principle [60, 62]). However, it still remains challenging to achieve completely interference-free detection in complex conditions even with enzyme modification.

Amperometric sensors have good features including high sensitivity, fast response, simple instrumental structure, and favorable miniaturization potential. Therefore, they can determine the concentration of specific compounds in liquid or gaseous media, which allows for their increasing potential applications in health diagnosis fields such as body fluid analysis [53, 58].

Amperometric sensors also have a major limitation, i.e., their restricted selectivity, which means that an overlapping background current is still produced in coexisting interferents with similar redox potentials. In addition, their long-term stability is further restricted by electrode fouling, membrane degradation, and enzyme inactivation.

#### Brief Summary

The primary sensing mechanisms are listed with their corresponding characteristics in Table 1. Except these five most prevalent sensing mechanisms, there are numerous other mechanisms, including impedance [105], magnetic [106–108], force-induced thermal field conversion [43], electrochemical [109, 110], optical [111], computer vision [106], and pyroelectric [112]. These mechanisms also have unique advantages in specific applications although they are not widely applied.

**Table 1:** Summary of key features of fundamental sensing mechanisms

Types of Mechanism	Advantages	Disadvantages	Scope of applications
Resistive sensors	Simple structure, high versatility, low cost, wide application range	Poor anti-interference capability	Stretch [67, 68], compression [69–73], bending [74], shear [75, 76], twisting [75], vibrations [69], sound [69], temperature [77, 78], humidity [49, 77], bioelectricity [47, 79], chemical species [55]
Capacitive sensors	Wide detection range, broad linear response region [91, 92], good dynamic response, low-power consumption, non-contact proximity sensing [84, 90]	Poor anti-interference capability [84]	Stretch [80, 81], compression [82–87], bending [84, 88], shear [87], twist [88], vibration [44, 86], and humidity [89, 90]
Triboelectric sensors	Self-powering [93–95], sensitive to transient signals, broad material compatibility [96, 97]	Unsuitable for static measurements, poor long-term stability [94]	Stretch [98, 99], compressive [100], bending [100], shear [82], humidity [49], temperature [49], and vibration [93]
Piezoelectric sensors	Self-powering [101, 102], highly sensitive to transient and high-frequency signals, favorable dynamic characteristics [104], a broad frequency bandwidth	Unsuitable for static measurements	Compression [101–103], shear [42], torque [43], acoustic waves [45], vibrations [104]
Amperometric sensors	High sensitivity, fast response, simple instrumental structure, favorable miniaturization potential	Limited anti-interference capability, poor long-term stability	Specific molecules in liquid/gaseous media [53, 58]

Different sensing mechanisms are usually used according to the specific requirements of the target applications. For instance, resistive sensors have a broad scope of applicability and are suitable for simultaneous detection of multiple signals; capacitive sensors are used in proximity sensing; triboelectric sensors are used for low-frequency transient signals; piezoelectric sensors are used in high-frequency/vibration situations; and for amperometric sensors, specific enzymes must be used according to the electrochemical properties of the target molecule. Every sensing mechanism has its own advantages and disadvantages [113]. Therefore, multimodal sensors with multiple mechanisms have attracted increasing attention (elaborated in Sect. 4.2).

Beyond their different applications, different sensing mechanisms require different materials and structures according to their distinct physical or chemical principles, which conversely determine the final performance metrics of the sensors.

Resistive sensors function according to the response of the overall resistivity of the material to external stimuli. Therefore, in addition to the intrinsic carrier properties (concentration and mobility) and the resistivity (*ρ*) of the material, these factors shall also be considered, i.e., the morphology of the conductive network, the interfacial contact state, and their evolution under external stimuli, all of which are directly correlated with their sensitivity and dynamic range.

Capacitive sensors are highly dependent on the dielectric properties of the dielectric layer material, including the relative permittivity (*ε*_*r*_) and loss tangent [114]. A high *ε*_*r*_ enhances sensitivity, and a low loss tangent reduces signal attenuation and therefore improves signal stability and signal-to-noise ratio. Improved properties contribute to enhanced linearity, response speed, and anti-interference capability of the sensors.

Triboelectric sensors function because of the difference in electron affinity between materials to induce electric charge. Therefore, their key performance parameters include the surface charge density of the material, the charge retention capability, surface roughness, wear resistance, and the combination of different materials. The amount of induced triboelectric charge increases with the difference in electron affinity between disparate materials, thus resulting in better sensor sensitivity and transient signal capture capability [49]; favorable surface roughness increases the contact area and boosts sensitivity; and high wear resistance mitigates charge decay and improves long-term stability.

For piezoelectric sensors, the core parameters include the piezoelectric constant (efficiency of charge output in response to applied force), electromechanical coupling coefficient, and mechanical strength of piezoelectric materials. The conversion efficiency from mechanical strain to electrical signals increases with the piezoelectric constant, thereby improving the sensitivity; the sensor’s dynamic characteristics and structural stability depend on appropriate electromechanical coupling coefficient and mechanical strength, which collectively determine the sensor’s sensitivity to dynamic forces and frequency response range.

Amperometric sensors function depending on the catalytic activity, anti-interference capability, and service life of the working electrode (including immobilized enzymes). Their sensitivity, limit of detection, anti-interference capability, and service life are influenced by the type of enzyme, the electrochemical properties and microstructure (which affects specific surface area) of the electrode material, and the design of the encapsulation structure.

In summary, the intrinsic correlations between different sensing mechanisms and material properties ultimately translate into quantifiable sensor performance metrics. In practice, the corresponding sensing mechanism and material properties should be matched according to detection requirements (e.g., static/dynamic measurement, precision, power consumption, environmental adaptability). Precise regulation of material parameters enables the optimization of sensor performance and its adaptation to engineering applications.

### Performance Evaluation of Flexible Sensors

The primary performance parameters of sensors include sensitivity (S), range, limit of detection (LOD), linearity, response time (*t*_res), recovery time (*t*_rec), repeatability, resolution, bandwidth, and hysteresis error. Their definitions are summarized in Table 2.

**Table 2:** Performance parameters and definitions of sensors

Performance parameters	Definition
Sensitivity (S)	Ratio of the change in sensor output (ΔOutput, e.g., resistance, voltage, current, capacitance) to the corresponding change in the input (ΔInput, e.g., strain, pressure, curvature, temperature, concentration of chemical substances, intensity of light). Its dimensions and units depend on the specific input and output signal types. In resistive strain sensors, this is commonly normalized and reported as the gauge factor (GF)
Limit of detection (LOD)	Minimum input quantity that the sensor can reliably detect
Range	Minimum to maximum input values that a sensor can reliably detect
Linearity	Degree to which the sensor’s actual input–output characteristic curve approximates a straight line. In scientific research, the coefficient of determination (R^2^) from linear regression is predominantly used (highly sensitive to the measurement range). High linearity over a wide range is considered highly beneficial for reducing algorithm development complexity and enhancing the accuracy and operational range of the sensing system [32]. Otherwise, more demanding requirements are placed on signal conditioning circuits and sophisticated processing algorithms [70]
Response time (t_res)	Time required for sensor output to reach its theoretical steady-state value following a step change in the input quantity. It is commonly quantified by t90, which refers to the time required for the sensor output to reach its 90% steady-state value after a step change in the input quantity
Recovery time (t_rec)	Time required for sensor output to return to its baseline value after the input quantity is restored to its original state. It is commonly quantified by t10, which refers to the time required for the sensor output to fall to 10% of the steady-state value after the input quantity is restored to its original state. Due to viscoelastic hysteresis in flexible materials, recovery time may exceed response time and exhibit asymmetric loading/unloading behavior [115]
Repeatability	Closeness of agreement among multiple repeated output readings obtained under identical conditions (repeatability data obtained under different conditions cannot be directly compared), with the same input applied at a certain frequency and quantity
Resolution	Smallest change in input that the sensor can reliably distinguish. It is usually expressed in the units of the input quantity (e.g., Pa, N, %, °C, mg)
Bandwidth	Range of input signal frequencies over which the sensor can respond effectively
Hysteresis error	Maximum difference between the input–output characteristic curves of the sensor during increasing (upscale) and decreasing (downscale) inputs under identical conditions

Notably, accuracy, signifying the closeness of agreement between a sensor’s measurement value and the true value of the input, is the most widely recognized performance parameter. However, it is actually a composite indicator that encompasses various factors such as linearity, hysteresis, and repeatability. As an overall evaluation metric for the entire sensing system, it generally should not be used alone to evaluate the sensor element itself.

## Materials

**Fig. 4:**
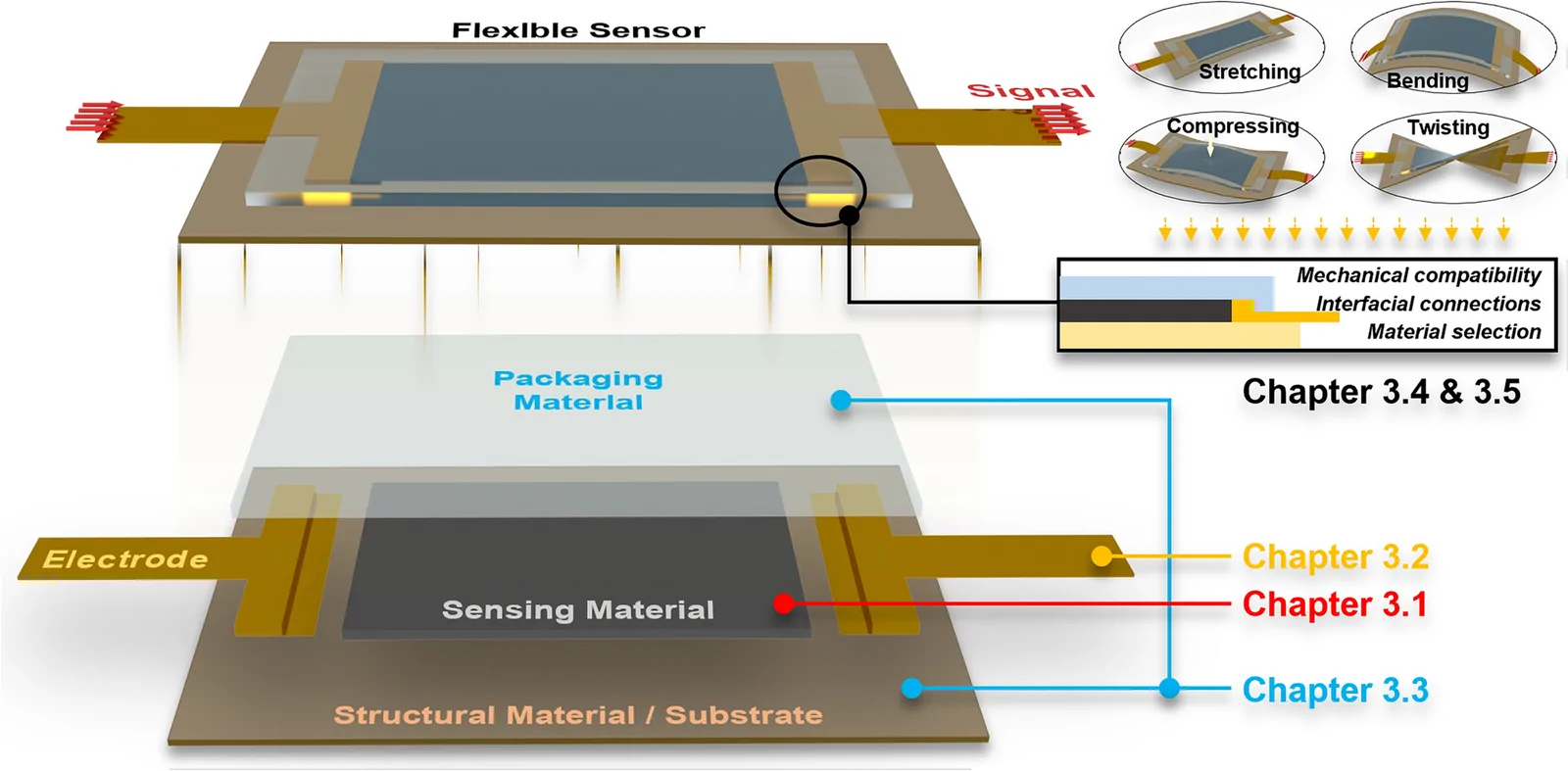
Flexible sensors’ three types of materials by functions: sensing, electrode, and structural/packaging materials

As the physical embodiment of sensing mechanisms and structures, material choice serves as the core of sensor performance. In the field of flexible sensors, materials can be categorized into three types according to their functional roles: sensing, electrode, and structural/packaging materials, as shown in Fig. 4. Through efficient synergistic design of these materials, flexible sensors can conform to complex curved surfaces and diverse real-world applications, and thus to achieve more accurate and versatile detection performance.

### Types and Performance Optimization of Sensing Materials

Sensing materials are core functional unit of sensors, which first detect specific external physical, chemical, or biological stimuli and then directly convert them into quantifiable electrical signals. The sensing materials can be categorized into three types:

- 1. Intrinsic sensing materials consist of a continuous functional layer formed directly from single or multiple functional materials with inherent stimulus–response characteristics, whose electrical properties vary in response to external stimuli. A notable advantage of these materials lies in their typically high sensitivity, low LOD, and rapid response and recovery [116]. However, the absence of a self-supporting structure necessitates reliance on structural and packaging components to maintain mechanical integrity.
- 2. Composite elastomer sensing materials are elastomers formed by dispersing functional materials within a polymer matrix (organized in Sect. 3.3 and Table 3). The polymer matrix offers a stable structural skeleton and effective bonding, thereby conferring superior mechanical buffering capacity and structural integrity. Furthermore, they can accommodate a wider variety of sensor structural designs, demonstrating greater design flexibility.
- 3. Conductive gels are composed of a polymer network and an ionic solution (or liquid ionic compounds), and include hydrogels [117], organo-gels, iono-gels [118], and hybrid organo-hydrogels [119]. These materials exhibit exceptional stretchability (manifested as extremely low modulus and high elongation at break), outstanding biocompatibility [120–122], strong tissue adhesion [121], low interfacial impedance [111, 123], self-healing capability, and high optical transparency, rendering them irreplaceable in bioelectronic applications [124–126]. However, because ionic conductors primarily rely on ionic conduction, their electrical conductivity is visibly lower than that of conventional conductive materials such as graphene and metals, due to significant disparities in charge-carrier density and mobility. Moreover, their environmental durability and long-term stability have long been constraints on practical application. In recent years, several performance optimization strategies have been developed, including PEDOT:PSS-based hydrogels [105, 127], nanocomposite modification [42], and the construction of mesh-like composite microstructures [120, 122], which have increased the electrical conductivity of some materials close to the level of certain conductive thin films. Furthermore, iono-gels and organo-gels with good weatherability and stability have also attracted increasing attention [128].

Representative sensing materials and their typical applications include the following:

- 1. Materials responsive to stretch, pressure, bending, and temperature for resistive sensors: CNTs [129–138], MXenes [139, 140], carbon black (CB) [89], graphene [141], Ag flakes/particles [74], Ag nanowires (AgNWs) [142], high-entropy alloy nanoparticles [67], organic semiconductor F4TCNQ-Pg_3_2T-TT [40] and P3HT [132], PEDOT:PSS [78, 143], conductive gels [26, 105], Cr/Au [73] and composite liquid metal nanomaterials [144–146].
- 2. Dielectric layers for capacitive sensors: conductive gels [91, 92], poly(vinylidene fluoride) (PVDF) [88], thermoplastic polyurethane (TPU) [85], polydimethylsiloxane (PDMS) [93, 147], Ecoflex [136], and gels [140].
- 3. Piezoelectric materials for piezoelectric sensors: lead zirconate titanate (PZT) [101, 102], aluminum nitride (AlN) [102], PVDF [103, 104], and conductive gels [42].
- 4. Humidity-sensitive materials for humidity sensors: BC_2_N [148], MXenes [149], graphene [143], and conductive gels [150].
- 5. Bio-electrodes with low interfacial impedance: conductive gels [121, 123].
- 6. Temperature-sensitive materials: nickel oxide (NiO) [66], MXenes [77], reduced graphene oxide (rGO) [78, 151], and PEDOT:PSS [78].
- 7. Molecule-sensitive materials: indium gallium zinc oxide (IGZO) [55], Zn [110], CoP/MoS_2_ [152], Bi_2_S_3_ [153] for NO_2_, In_2_O_3_ for H_2_S [154], Pd/In_2_O_3_ [155] and SiO_2_/WO_3_ [156] for H_2_, SnO_2_ for 3-hydroxy-2-butanone [157], FeCu/NC catalyst [109] for NO_2_ and H_2_S, SnPx/rGO [158] for NH_3_, crystalline porous framework material for SO_2_ [159].

In addition, other specialized sensing materials include polyaniline (PANI) for pH sensing [53] and Pt nanoparticles as sensitive elements in chemical sensors [160]. In addition to our work, sensing materials have also been systematically reviewed in several existing studies [161–163].

### Selection and Application of Electrode Materials

Electrode materials provide a stable electrical contact interface with the sensing materials, efficiently and reliably collect electrical signals from them, and transmit these signals to peripheral circuits and subsequently to communication and processing systems. They should ideally possess high electrical conductivity, strong compatibility (electrical, mechanical, thermal) with the sensing materials, excellent environmental stability, and sufficient mechanical stability and adhesion. To match the overall flexibility of the sensor, they are typically fabricated with a sufficiently thin geometry, taking the form of foils, films, or meshes.

Commonly used electrode materials comprise the following:

- 1. Metal foils, such as Au [164], Ag [88], Cu [165], Ti [82], Cr [105], Pt [104], Fe [101], and Mo [102].
- 2. Metallic nanomaterials, such as silver paste [166, 167], Ag nanowires [168], Ag nanoparticles [81], Au nanoparticles [111], and Cu nanowires [94].
- 3. Carbon-based materials, such as CNTs [169], graphene [170], and CB [171].
- 4. Conductive oxides and polymers, including ITO [82] and PEDOT:PSS [171].

Notably, the distinction between sensing materials and electrode materials often remains ambiguous in flexible sensors [74]. As expressed in **Eq. **(1), the deformation of a conductive material is inevitably reflected in its electrical characteristics. Consequently, during sensor operation, not only does the intended stimulus acting on the sensing material produce signal changes, but the unintended deformation of the electrodes themselves also generates electrical signals, introducing interference that compromises measurement accuracy and signal-to-noise ratio. Considering this, structural design, circuit configuration, and signal processing algorithms must be systematically considered to effectively suppress, or exploit [75, 76], the interference potentially caused by electrode deformation.

As a result of this challenge, liquid metal materials, which exhibit strain-insensitive electrical behavior and good biocompatibility [172–175], have attracted widespread interest in the design of high-precision, interference-resistant flexible sensors [176, 177]. Characteristically, their resistance increases by only about 4.1% even when stretched to 800% [174], and remains stable after 10,000 stretching cycles at 30% strain [50]. Therefore, this exceptional electrical stability enables the fabrication of stretchable micro-processing systems [178].

### Impact of Structural, Packaging Materials & Polymers on Sensor Performance

Structural and packaging materials (including substrates and, in their absence, the sensor configurations known as electronic tattoos [13, 67]) fulfill several core functions. First, they provide fundamental flexibility and stretchability of sensors, provide mechanical support to maintain the sensors’ specific functional geometry, electrically isolate to prevent leakage currents and mitigate the risk of short circuits inside sensors and ensure essential biocompatibility, particularly for wearable or implantable applications. They also protect the sensing and electrode materials from mechanical abrasion, moisture, oxygen, dust, chemical corrosion, and biofouling among all the environmental interferences, thus ensuring structural integrity and long-term reliability. They shall also be designed for the efficient transmission of the target external stimuli to the sensing region and help suppress the generation of crosstalk.

Therefore, it is critical to choose and design proper structural and packaging materials. This not only directly influences the sensor’s stimulus–response performance (e.g., sensitivity, linearity, hysteresis) but also profoundly affects the overall reliability, service life, environmental adaptability, and user comfort.

Flexible sensors now extensively using flexible polymer materials owing to their exceptional flexibility, outstanding processability, light weight, and abundant material options, which are almost indispensable [21]. Polymer materials, as the main choice for structural and packaging materials, directly determines the overall mechanical compliance and long-term stability of sensors.

Proper polymers used as structural and packaging materials should have the following features: appropriate mechanical properties (e.g., modulus, strength, elongation at break) to effectively transmit and match mechanical strains; suitable rheological properties, thermomechanical properties, and cohesive energy density to achieve excellent processability; favorable adhesion to other materials for enhanced reliability of interfacial bonding between different components of sensors; adequate electrical properties (e.g., high electrical insulation, a stable low dielectric constant) to preserve signal quality in sensors; and the other properties needed for particular applications (e.g., biocompatibility, optical transparency, self-healing ability, recyclability, and biodegradability).

In addition, polymers are widely used in the various previously mentioned elastomer materials and realize the following functions: matrix of composite elastomer sensing materials, the main constituent of dielectric layers for capacitive sensors, and the triboelectric layers for triboelectric sensors [114]. They act as a stable supporting and dispersing medium for the functional components (e.g., conductive fillers, dielectric particles), which not only ensure their uniform distribution without agglomeration or detachment during mechanical deformation, but also preserve the structural integrity and functional stability of the sensing layer [179].

It is critical to use proper functional components in elastomers, because it involves a trade-off between the functionality (e.g., electrical conductivity, dielectric constant, surface charge density) and the mechanical flexibility [66]. Taking the percolation threshold phenomenon of conductive composites as an example [68], excessive pursuit of high electrical conductivity may render elastomers rigid and brittle, making them unable to meet flexibility requirements. This is also a key reason why conductive materials with a large aspect ratio (represented by nanowires [142], which help significantly reduce the percolation threshold) are widely favored.

For such elastomer materials, in addition to the parameter considerations mentioned in the structural and packaging materials, extra attention should be paid to indicators such as polymer density, modulus, elongation at break, viscosity, and surface energy. These parameters influence the acceptable filler content range, the dispersibility of functional components in the polymer matrix, and the wettability between functional components and polymer molecules.

Common polymer materials primarily include PDMS, polyimide (PI), polyethylene terephthalate (PET), thermoplastic polyurethane (TPU), Ecoflex silicone rubber, styrenic block copolymers (SBS/SEBS), and various textiles and fibrous substrates. The characteristics of common polymer materials have been summarized in Table 3.

**Table 3:** Comparison of commonly used polymer materials for sensors

Materials	Mechanics	Thermology	Other characteristics	Functional applicability
PDMS	Hardness: low; Stretching: low modulus, low strength, high elongation after fracture (~ 100%); Compressing: low modulus, high strength; Bending: low modulus, high strength	Thermosetting; Service temperature: -100 ~ + 200 °C; colorless, transparent	Stretchable; Good biocompatibility; Sensors involving light [50]; Adjustable mechanical properties by composition [31, 201]	Substrate [31, 50, 68, 77, 80, 89, 101, 180, 185, 187, 202–204]; Matrix [28, 29, 43, 68, 70, 127, 142, 160, 205]; Structure [72, 75, 81, 82, 84, 202]; Package [31, 42, 44, 50, 52, 58, 68, 71, 76, 92, 98, 127, 168, 180, 206]; Dielectric layer [93, 147]
PI	Hardness: high; Stretching: high modulus, high strength, low elongation after fracture (< 30%); Compressing: high modulus, high strength; Bending: high modulus, high strength;	Thermosetting; Service temperature: -200 ~ + 300 °C; tawny, transparent	Non-stretchable; Heat-resisting [74]	Substrate [43, 44, 46, 53, 58, 66, 74–77, 79, 84, 102, 127, 150, 160, 185, 207–209]; Package [43, 101, 102];
PET	Hardness: high; Stretching: high modulus, high strength, low elongation after fracture (< 50%); Compressing: high modulus, high strength; Bending: high modulus, high strength;	Thermoplastic; Service temperature: -20 ~ + 120 °C; colorless, transparent	Non-stretchable; Lower minimum bending radius; Suitable for bending sensing	Substrate [31, 40, 42, 53, 77, 93]; Package [207]; Structure [123];
Ecoflex	Hardness: low; Stretching: low modulus, low strength, extremely high elongation after fracture (> 600%); Compressing: low modulus, high strength; Bending: low modulus, high strength	Thermosetting; Service temperature: -20 ~ + 150 °C; Milky white, translucent	Highly stretchable [41, 80]; Good biocompatibility [80]; Adjustable mechanical properties by composition [171, 191]; Force-transfer component [44, 210]	Package [44, 70, 75, 76, 80, 99, 102, 106, 108, 125, 167, 205, 209]; Matrix [81, 99, 136]; Substrate [94, 171, 205]; Tribo-layer [99, 100]; Dielectric layer [136]
TPU	Hardness: low; Stretching: low modulus, low strength, high elongation after fracture (> 200%); Compressing: low modulus, high strength; Bending: low modulus, high strength	Thermoplastic; Service temperature: -20 ~ + 80 °C; colorless, transparent	Stretchable; Suitable for spinning [85, 116, 143], extrusion [85], 3D printing [46, 190], hot pressing [71]; Solvent recovery and solvent welding [23]	Substrate [26, 48, 86, 122, 143, 186]; Matrix [46, 47, 66, 85, 86, 129, 144]; Structure [71, 190]; Package [85]; Dielectric layer [85]
PU	Hardness: high; Stretching: low modulus, high strength, low elongation after fracture (< 50%); Compressing: low modulus, high strength; Bending: low modulus, high strength	Thermosetting; Service temperature: -20 ~ + 120 °C; light-yellow, translucent	Stretchable; Biocompatible [175]	Substrate [79, 123, 168, 202]; Matrix [146]; Package [26, 46, 79, 92, 105, 202, 211]
Epoxy resin	Hardness: high; Stretching: high modulus, high strength, low elongation after fracture (< 10%); Compressing: high modulus, high strength; Bending: high modulus, high strength	Thermosetting; Service temperature: -50 ~ + 200 °C; light-yellow, transparent	Non-stretchable; Suitable for high-temperature sensing [74]; Adjustable mechanical properties by composition [198]; Shape memory [198]; Good interconnection [204]; solvent welding [192]	Matrix [74, 81, 204]; Package [147, 198, 204, 211, 212]; Inter-connection [33, 58]; Transfer template [202]
PVDF	Hardness: high; Stretching: high modulus, high strength, high elongation after fracture (< 100%); Compressing: high modulus, high strength; Bending: high modulus, high strength	Thermoplastic; Service temperature: -20 ~ + 150 °C; light-yellow, translucent	Suitable for piezoelectric sensors [88, 103, 104, 206]; Good biocompatibility [88]	Substrate [88]; Package [206]; Dielectric layer [87, 88]
SBS/SEBS	Hardness: low; Stretching: low modulus, low strength, extremely high elongation after fracture (> 400%); Compressing: low modulus, high strength; Bending: low modulus, high strength	Thermoplastic; Service temperature: -20 ~ + 80 °C; colorless, transparent	Solvent welding [60]; Good biocompatibility [60]	Package [127, 181]; Substrate [60]; Dielectric layer [132]; Matrix [145]
Fabric	Material-, process-, and structure-dependent	Material-dependent	High specific surface area, air permeability; Suitable for chemical gas/liquid, humidity sensing and wearable applications [45, 46, 48, 52, 78, 116, 131, 133, 143, 164, 213]	Structure [45, 94, 112, 166]; Substrate [92, 166, 186]

PDMS is one of the most widely used flexible materials due to its excellent stretchability, biocompatibility, high optical transparency, and good processability. It can be used as substrate [180], matrix [142], packaging material [168], structural component [140], as well as dielectric or triboelectric layer [93, 94]. It is especially suitable for flexible devices integrating optical sensing or display elements because of its high transparency [50]. Furthermore, it can be tailored for specific requirements through compositional modification [47].

PI [101, 102] and PET [82] can be easily made into thin, flexible films used as substrates, owing to their outstanding tensile and flexural strength, moderate elongation at break, and inherent dimensional stability, despite their relatively high flexural modulus. These materials have been extensively used in applications that need significant bendability and long-term reliability. Notably, however, they are generally unsuitable for applications that need substantial stretching.

Materials such as TPU [46, 47], Ecoflex silicone rubber [136], and SBS/SEBS [60, 181] are often used as polymer matrices or packaging materials. They are used in sensing scenarios that need dynamic deformation due to their good elasticity, flexibility, biocompatibility, and considerable stretchability.

Epoxy resins have excellent bonding to most materials despite their rigidity and brittleness. Therefore, they are commonly used as the matrix of conductive adhesives in applications where component interconnection are involved [58, 182].

Additionally, textile materials, e.g., cotton cloth [94], spandex [82], glass fiber fabric [92], nylon fabric [92], polyester fiber [112], and paper-based substrates [183] are also used as substrates or supporting structures in specific types of flexible sensors, leveraging their unique properties such as breathability, wearing comfort, or foldability.

### Basic Principles for Material Selection of Sensors

Materials innovation is of first importance for research. The intrinsic constitution of a material, including its elemental composition, chemical bonding, and micro- and nano-structure, determines its unique spectrum of intrinsic properties (e.g., mechanical, chemical, thermodynamic, electrical, optical, magnetic, thermal, acoustic, electrochemical, thermomechanical, self-healing capability, and reconfigurability). These properties, in turn, dictate the material’s suitability for specific applications and the methods by which it can be effectively processed and manufactured. No material is exclusively designated for a single task. Therefore, depending on the designer’s intent and strategic considerations, the same material can be adapted to perform distinct roles across different applications [194, 195].

**Fig. 5:**
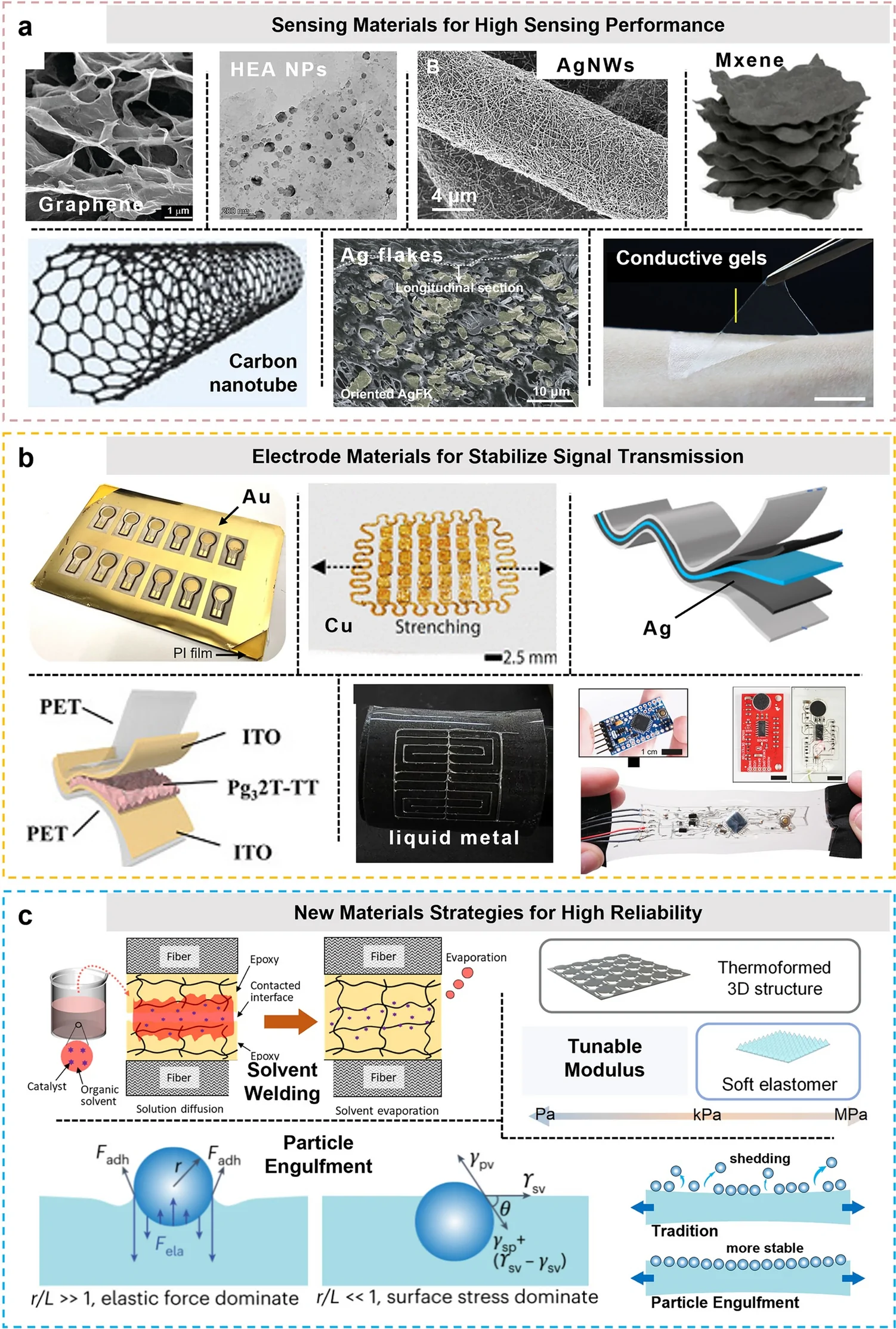
Schematic diagram depicting materials for sensors. **a** Sensing materials: graphene [184] Copyright 2010, American Chemical Society, HEA nanoparticles [185] Copyright 2024, Springer Nature, AgNWs [186] Copyright 2024, Elsevier, MXene [116] Copyright 2025, Springer Nature, CNTs [187] Copyright 2025, American Chemical Society, Ag flakes/particles [188] Copyright 2025, Springer Nature, conductive gel [120] Copyright 2025, The American Association for the Advancement of Science. **b** Electrode materials: Au [147] Copyright 2025, American Chemical Society, Cu [189] Copyright 2025, The American Association for the Advancement of Science, Ag [31] Copyright 2025, John Wiley and Sons, ITO [40] Copyright 2025, John Wiley and Sons, liquid metal [190] Copyright 2025, John Wiley and Sons, liquid metal electrode-based flexible and stretchable monolithic Arduino pro mini system [191] Copyright 2024, The American Association for the Advancement of Science. **c** Solvent welding [192] Copyright 2025, Elsevier, Tunable-modulus/stiffness design [33] Copyright 2025, The American Association for the Advancement of Science, and Particle engulfment [193] Copyright 2025, Springer Nature

Commonly used materials in the sensor field are shown in Fig. 5, and a summary of representative sensors across these categories is provided in Table 4.

**Table 4:** Materials, structure and performance summary of some representative sensors

Quantities	Modals	Structure	Material, structure and patterning process	Sensitivity	(LOD) Range	Linearity (%)	T_res/T_rec (ms/ms)	Repeatability (load/frequency/cycles)	Other characteristics	Ref
Compress	Resistive	Micro-contact	PET/ITO/Ag-MnO_2_/ITO/PET Main process: laser cutting	-	(0.01 Pa) 0 ~ 200 kPa	–	3.8/5.5	–/–/3000	Maximum bandwidth = 5000 Hz	[69]
		Porous	CNT/CB/PDMS foam Main process: template method + ultrasonic load	0.36 kPa^−1^	0 ~ 225 kPa	99.9%	–/–	–/–/180000	Maximum bandwidth = 2 Hz	[165]
		Mesh/woven	Polyester fiber@CNTs fabric Main process: solution impregnation method	1.7	0 ~ 80% (press)	–	20/60	–/–/10000	–	[112]
		Micro-protrusion	Crocodile(PDMS/rGO)-Shark(PDMS/rGO) Main process: template transfer method	18.2 kPa^−1^;	1 ~ 80 kPa	–	21/28	3%/–/20000	–	[202]
		Micro-contact + interdigitated + Sandwich	TPU fabric/Mxene-PEDOT:PSS/PDA-TPU/Mxene-PEDOT:PSS Main process: electro-spinning + screen printing + spraying	270.0 kPa^−1^ (0 ~ 11.5 kPa) 78.8 kPa^−1^ (11.5 ~ 38.3 kPa)	0 ~ 38.3 kPa	99.8% (0 ~ 11.5 kPa) 99.6% (11.5 ~ 38.3 kPa)	78/90	–	–	[143]
	Capacitive	Micro-contact + mesh	Cu/TPU-PANI/Cu Main process: electro-spinning	0.0611 kPa^−1^ (0 ~ 1.675 MPa) 0.0185 kPa^−1^ (1.675 ~ 11.480 MPa) 0.00776 kPa^−1^ (11.48 ~ 23 MPa)	(3 Pa) 0 ~ 23 MPa	99.8% (0 ~ 580 kPa) 99.1% (10 ~ 23 MPa)	175/75	–/–/10000	–	[66]
		Micro-protrusion	PVA-H_3_PO_4_-Gly IG/PVDF/PVA-H_3_PO_4_-Gly IG Main process: template transfer method + spin coating	1.31 kPa^−1^ (0 ~ 10 kPa) 0.12 kPa^−1^ (10 ~ 50 kPa)	(50 Pa) 0 ~ 50 kPa	–	44/52	–/–/10000	–	[87]
		Micro-protrusion + Porous + Sandwich	Ni foam/mesh fabric/monofilaments/mesh fabric@PVA IG/Ni foam	49.76 kPa^−1^	0 ~ 1550 kPa	94.84%	170/190	–/–/5000	–	[83]
Stretch	Resistive	Micro-contact	PBSE/AgNWs/MXene/TPU fabric Main process: blade coating	1158.1	0 ~ 57%	–	30/1000	5%/–/2800	Maximum bandwidth = 2 Hz	[186]
		Porous	EGaInSn LM/PDMS Foam Main process: template method	GF = 8.3	1 ~ 120%	99.6%	60/81	–/–/10000	Maximum bandwidth = 2.56 Hz	[205]
		Micro-contact + interdigitated	TPU fabric/Mxene-PEDOT:PSS Main process: electro-spinning + screen printing	50.8 (0 ~ 50%)	0 ~ 200%	99% (0 ~ 50%)	260/410	–/–/5000	–	[143]
Humidity	Resistive	Micro-contact + interdigitated	TPU fabric/Mxene-PEDOT:PSS/GO Main process: electro-spinning + screen printing + spraying	0.94375 (%/%RH)	0 ~ 100% RH	–	40,300/27700	–	–	[143]
	Capacitive	Porous + interdigitated	SA conductive gel/Cu Interdigital electrode/PI Main process: spinning coating + solvent-induced phase separation	9% RH^−1^ (5% ~ 30% RH) 235% RH^−1^ (30% ~ 70% RH) 1580% RH^−1^ (70% ~ 100% RH)	5% ~ 100% RH	–	900/500	–/–/100	Resolution = 0.01% RH	[150]

'-' denotes that corresponding parameters are not mentioned in the original work. Performance data adopted in this table are as reported in the original literature

In the material selection process for flexible sensors, the core functional performance indicators, clarified based on the requirements of detection scenarios, should always be given top priority [196]. These indicators include high specific selectivity needed in chemical substance detection (e.g., gas molecules, liquid small molecules, biomarkers), indispensable biocompatibility (e.g., low toxicity, excellent tissue adaptability, and immunological inertness) needed in biomedical applications (e.g., wearable health monitoring, implantable sensing), rapid response and precise conversion capabilities to environmental stimuli, e.g., light, heat, magnetism, and humidity, as well as the functional indicators needed in special applications (e.g., special morphological designs like porous, layered heterostructures, or environmentally friendly properties such as self-healing, reparability, and degradability/recyclability).

Once the core functions are fulfilled, the basic mechanical properties of the selected materials, the degree of mechanical compatibility between different parts, and the quality of their interfacial connections throughout the structure remain important factors to be considered, all of which are essential for flexible sensors, because “flexibility” is their core technical feature. In flexible sensors, the key parameters of materials include Young’s modulus, flexural modulus, tensile strength, elongation at break, wettability, and adhesion strength with other materials, all of which directly influence the critical sensor characteristics such as the sensitivity, wear comfort, detection limit, measurement range, stability, and robustness.

It is critical to use materials with appropriate mechanical properties, so as to ensure sensor performance [197]. The performance parameters do not follow the principle of “higher is better” or “lower is better”, but should be matched to the specific target applications. For example, in strain sensors, materials with a low elastic modulus are usually used to achieve high sensitivity and a low detection limit. But an excessively low modulus degrades their structural stability, response speed, hysteresis performance, and interference resistance. It has been demonstrated that excessive elasticity reduces response speed, increases detection limits and significant hysteresis (PDMS [33], Ecoflex [115]). Therefore, it is critical to use a material with a suitable modulus [27, 98].

Sensors’ overall performance is also influenced by other two crucial factors, the degree of mechanical compatibility and the quality of their interfacial connections [171]. The first one influences the interfacial stress concentration, incompatible deformation, and interlayer peeling caused by excessive modulus differences, which can ensure the mechanical stability of the overall structure under dynamic deformation. The second one includes interfacial bonding strength, connection stability under complex environments, and interfacial integrity under dynamic deformation conditions such as repeated bending, stretching, and folding. An interface should be optimally designed to ensure sufficient adhesion [72, 198] and rational mechanical matching [47] for maximized stress-transfer efficiency.

The primary application value of flexible sensors lies in their ability to adapt to complex dynamic deformation environments (such as human motion, equipment bending, and curved surface fitting). The aforementioned mechanical properties are all needed for the structural durability, sensing performance stability, and service life of the sensors during long-term dynamic operation.

### Performance Trade-Offs and Reliability Design

The ultimate goal of material design is to endow the device with satisfactory overall mechanical flexibility to meet application requirements while ensuring its functional reliability. In practice, however, compromises are often inevitable between the functionality and mechanical properties of materials to fulfill target demands. This leads to challenges such as the inherent mechanical mismatch and weak interfacial bonding strength between heterogeneous materials, as well as microdefects introduced during the fabrication, all of which can act as triggers for device failure. The main failure modes of flexible sensing devices have been summarized in Table 5.

**Table 5:** Main failure modes of flexible sensing devices

Failure modes	Failure mechanisms	Evaluation	Mitigation strategy
Interfacial delamination	Unsatisfactory mechanical compatibility and bonding between heterogeneous materials	Relative difference of moduli and elongation after fracture, Peel strength/ Interfacial shear strength, Cyclic bending tests	Gradient-modulus transition, Adhesives, Package-reinforced designs, Solvent welding, Mono-polymer-matrix system design, Tunable-modulus/stiffness design, Particle engulfment
Contact-resistance drift	Unstable electrical contact due to deformation	ΔR/R_0_ stability in cycle tests	Conductive adhesives, FPC integration, Dedicated electrode connectors, Mono-polymer-matrix system design
Fatigue	Mechanical degradation under long-term usage	Fatigue life (N_f_)	Stress-buffering layers, Gradient-modulus transition, Strain-delocalizing structures, Solvent welding, Underfill encapsulation
Environmental instability	Humidity/chemical corrosion effects on sensitive materials	Sensitivity fluctuation	Enhanced hermetic/hydrophobic packaging
Long-term signal drift	Creep or thermal variations of related materials	Baseline drift rate	Pre-conditioning/Pre-stretching, Algorithm compensation, Stable reference electrodes [76]

In this scenario, effective structural reinforcement and protection strategies must be implemented on the devices [199].

Common approaches include the use of (conductive) adhesives (e.g., the epoxy-based materials mentioned earlier [182]) as interconnecting and stress-buffering layers between heterogeneous materials, and local reinforcements. Package-reinforced designs—including enhanced encapsulation at fragile locations and redundant designs featuring large-area, high-volume coverage of packaging materials—are also adopted to increase the overall mechanical failure threshold of the device.

In recent years, several strategies have been advanced, including solvent welding (for repairing damaged regions or improving local bonding strength) [60, 192], mono-polymer-matrix system design for full devices (where the bonding strength between identical polymer matrices is generally more reliable) [31], tunable-modulus/stiffness design (enabling a smoother transition between flexible and rigid regions) [33], and particle engulfment (embedding nanomaterials into polymers to substantially enhance the bonding strength between sensing materials and substrates) [193], as shown in Fig. 5c. These approaches have been shown to enhance interfacial stability and long-term device reliability significantly [200].

Finally, the integration of sensing devices with back-end hardware systems constitutes the final step toward realizing their system-level functionalities. As hardware systems incorporate numerous rigid components including chips, resistors, capacitors, and printed circuit boards (PCBs), further efforts are still required to ensure the reliability of structural–hardware interconnections between the devices and the hardware.

In addition to robust dedicated electrode connectors such as terminal blocks, header and socket [75], the interconnections can be improved by adopting several approaches: using flexible printed circuits (FPCs) either as a bridge between the components or as a direct substitute for the rigid PCBs; and strengthening electrical interconnections between electrodes via conductive adhesive bonding or solder welding. All these methods can be used to withstand certain degrees of mechanical deformation while also providing robust and stable electrical pathways.

In addition, for final optimization, integrated packaging strategies can be used as follows: local reinforced electrode interconnections with flexible encapsulant, dissipating stress around rigid components with underfill materials, and constructing gradual stiffness transition layers at rigid–flexible interfaces with gradient-modulus materials.

Thus, the sensors materials are selected considering the factors from fundamental working principles to hardware packaging, so as to ensure the long-term reliability of the entire signal pathway from the flexible sensing front-end to the rigid processing back-end.

## Structure

The sensor’s structural design is pivotal in determining how effectively its intrinsic material properties can be exploited. By incorporating specific functional structures, the stimulus–response characteristics of sensors can be precisely tailored to targeted performance metrics. The structural design strategies for flexible sensors, from microscopic to macroscopic, primarily include: homogeneous, intrinsic, conductive elastomers; micro-contact structures; porous structures; geometric/topological designs; mesh/woven structures; micro-protrusion mechanical structures; and sandwich structures. At a more macroscopic scale, structural design must also incorporate higher-dimensional design considerations, while comprehensively accounting for core requirements including multifunctional integration, multidimensional adaptation, multimodal synergy, high-dimensional information perception, wide-area coverage, and anti-crosstalk capability.

### Designing Functional-Sensing Structures for Enhanced Performance

**Fig. 6:**
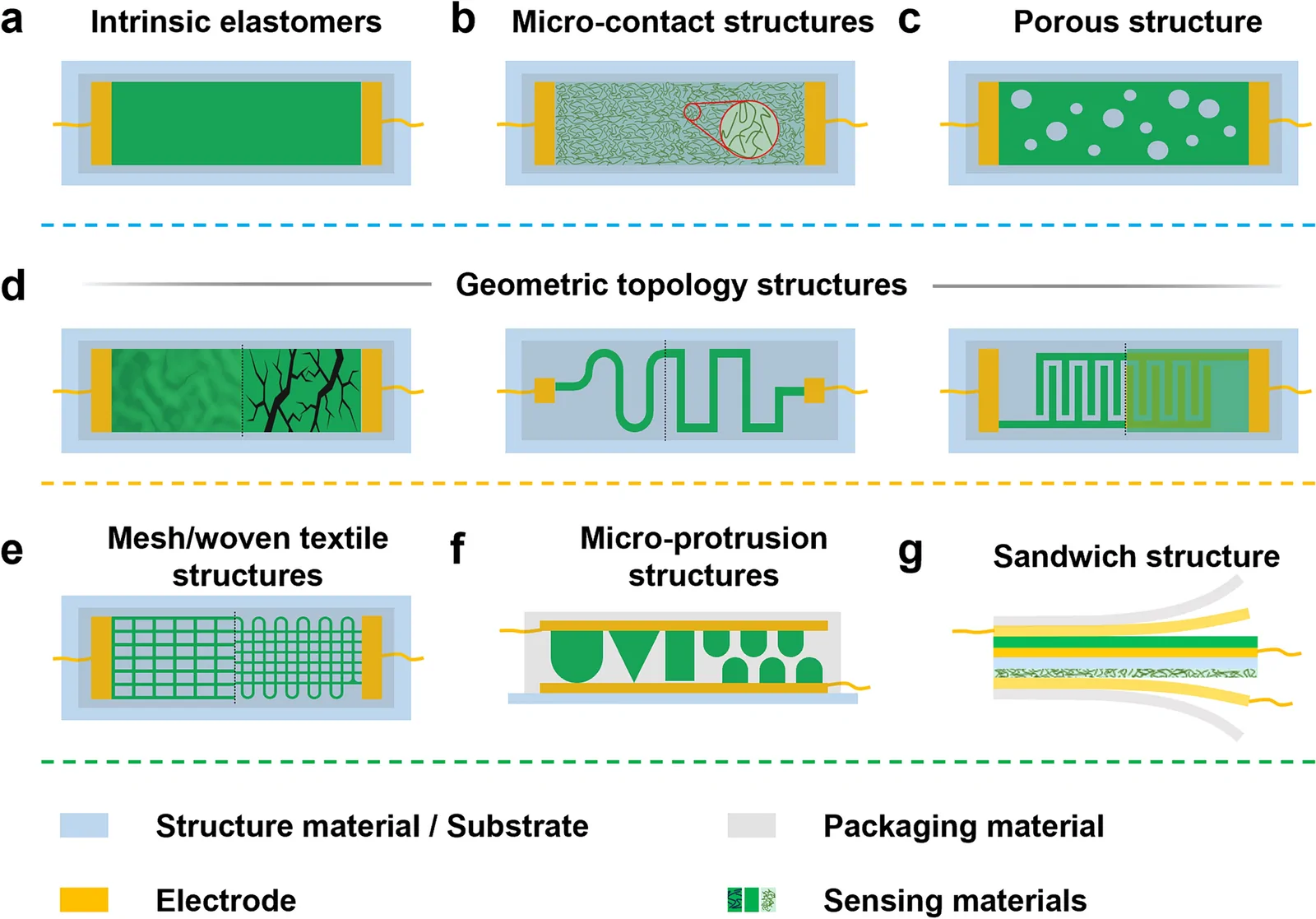
Main functional structures of flexible sensors: **a** Homogeneous intrinsic conductive elastomers, **b** Micro-contact structures, **c** Porous structures, **d** Geometric topological designs, **e** Mesh/woven structures, **f** Micro-protrusion mechanical structures, and **g** Sandwich structures

Typical functional-sensing structural design strategies are illustrated in Fig. 6.

**Fig. 7:**
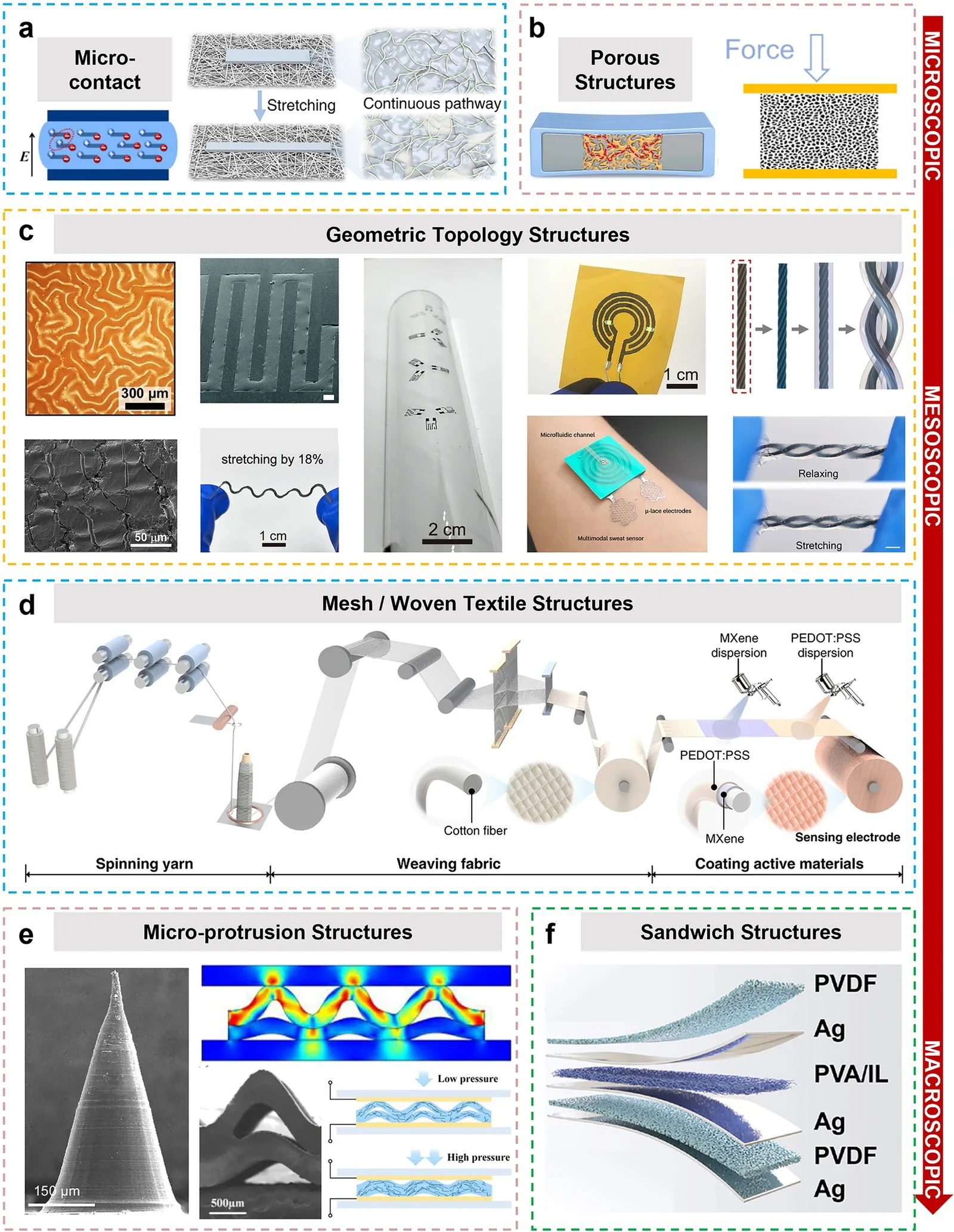
Typical example of sensor functional structure design. **a** Micro-contact structures: capacitive [187], resistive [143]. **b** Porous structures [165, 205]. **c** Geometric topology structures: predefined textures [181], predefined circuits/cracks [68], interdigitated [220], serpentine [67], spiral [67, 164], vortex [80]. **d** Woven/textile structure [166]. **e** Micro-protrusion structures [42, 70]. **f** Sandwich structure [88]. **a** Reproduced with permission from Ref. [187]. Copyright 2025, American Chemical Society. Reproduced with permission from Ref. [143]. Copyright 2025, John Wiley and Sons. **b** Reproduced with permission from Ref. [165]. Copyright 2025, The American Association for the Advancement of Science. Reproduced with permission from Ref. [205]. Copyright 2025, John Wiley and Sons. **c** Reproduced with permission from Ref. [181]. Copyright 2025, Springer Nature. Reproduced with permission from Ref. [68]. Copyright 2023, John Wiley and Sons. Reproduced with permission from Ref. [220]. Copyright 2022, Royal Society of Chemistry. Reproduced with permission from Ref. [67]. Copyright 2025, Springer Nature. Reproduced with permission from Ref. [164]. Copyright 2025, Springer Nature. Reproduced with permission from Ref. [80]. Copyright 2025, The American Association for the Advancement of Science. **d** Reproduced with permission from Ref. [166]. Copyright 2025, John Wiley and Sons. **e** Reproduced with permission from Ref. [42]. Copyright 2025, Springer Nature. Reproduced with permission from Ref. [70]. Copyright 2025, John Wiley and Sons. **f** Reproduced with permission from Ref. [88]. Copyright 2025, John Wiley and Sons

Micro-contact structures (Fig. 7a) improve sensing performance through micro- or nanoscale structural engineering, with micro-nano-percolation networks and microcrack structures serving as the two main types, both possessing advantages including fast response and high sensitivity [136, 214]: (1) Micro-nano-percolation networks utilize changes in the inter-filler spacing (Δd) within the composite sensing materials to induce abrupt transitions (governed by insulator-tunneling-contact mechanisms [215]) in electrical conduction [69], thermal conduction [66], or capacitance [187], thereby enabling the detection of corresponding stimuli. This mechanism is prevalent in composite elastomers, and it is largely insensitive to the overall macroscopic shape, allowing related materials to be fabricated into film/bulk [69, 136] or fiber/wire [141, 144] forms according to application requirements. (2) Microcrack structures can modulate the connectivity of electrical or magnetic pathways through the opening and closing of strain-induced submicron-scale cracks [214, 216]. This type of structure is commonly observed in thin films made from intrinsic sensing materials [137].

Porous structures (e.g., aerogels/foams [130], Fig. 7b) contain numerous micro/nanoscale pores. Their high specific surface area provides abundant micro-contact/active interfaces, which enhance sensor sensitivity [217, 218], the linear response range [219], and the measurement range [32]. These structures are typically fabricated using methods such as the sacrificial template technique [210], foaming/expansion [136], solvent-induced phase separation [150], supercritical drying, and freeze-drying [130]. Such structures can be adopted in resistive [217], capacitive [150, 219], and force-induced thermal field conversion [43] sensors. Their large specific surface area also makes them particularly suitable for sensing specific components in gases or liquids [150].

Well-established principles of topology and structural mechanics can be incorporated at the macroscopic level, which allows for enabling more targeted design of sensor structures.

In geometric topology structures, predefined patterns are used (Fig. 7c), such as circuits/textures/cracks [221–223], interdigitated shapes [131], serpentine layouts [115, 132], and spiral/vortex shapes [98], which allows for optimized conductive pathways of sensing materials. These structures regulate the distribution of sensing regions and tailor the electrical response characteristics. They increase the specific surface area [43], reduce overall stiffness, and significantly delay material failure by improving stress distribution [224], all of which enhance the device sensitivity, stretchability, LOD, stability, and reliability [223]. These structures can be fabricated using the following methods: pre-stretching [41, 194], laser cutting [214], inkjet printing [225, 226], 3D printing [139], photolithography [43, 76], dispensing [74], screen printing [67], blade coating [79, 185], spray coating [227], solvent evaporation [181], and template transfer [222].

In mesh and woven textile structures (Fig. 7d), it is possible to regulate strain distribution, conductive paths, specific surface area, triboelectric area, and overall flexibility by using different fiber materials and weaving patterns, which allows for improved stimulus–response characteristics [228]. The structures can be constructed using elastic fabric networks entirely from elastomers via extrusion/spinning followed by weaving [228] or 3D printing [187]. They can also be alternatively constructed by depositing sensing materials onto pre-formed mesh textiles [194] or individual fibers through dipping [78], drop-casting [133], or spraying [166, 227], followed by weaving [78, 112]. The high specific surface area, flexibility, and breathability of these designs make them particularly suitable for chemical vapor/liquid sensing, humidity detection, and wearable applications [213].

The micro-protrusion structure (Fig. 7e) represents a bio-inspired design that emulates biological skin [229, 230]. This enables directed and quantitative optimization of sensor input–output characteristics through controlled deformation of the functional layer and modulation of interfacial contact area. Such structures can be implemented in resistive [230], capacitive [231], triboelectric [93, 229], and piezoelectric [25, 42] sensors. As a result, these designs can significantly improve key sensor metrics such as sensitivity [232], linearity [233], and stability [230], making them suitable for detecting pressure [132], stretching [28, 81], shear force [42, 211], bending [28], and proximity, as well as enabling multidimensional sensing. These highly customizable geometric parameters, including protrusion shape (e.g., micro-hemispheres [71, 72], cones [42], whips [221], pillars [86], pyramids [142]), arrangement (e.g., ridges [70], rings [87]), spacing [72], size, array density [71, 72], and interlocking structures [234], allow for targeted performance optimization [231] and multi-axis force decoupling [87]. Combined with co-designed electrodes, they further facilitate multidimensional force sensing [211]. However, micro-protrusions are prone to plastic deformation or complete collapse under high pressure (typically > 150 kPa), leading to saturation that limits sensitivity, linear range, and working range, thereby presenting an inherent trade-off between high sensitivity and wide dynamic range [32]. This structure is typically constructed with template transfer [202], 3D printing [234, 235], screen printing [71], and laser cutting [233].

Sandwich structures (Fig. 7f), (note: Simple tri-layer stacks of substrate/single sensing layer/package are excluded from this discussion) have multiple advantages in sensing. (1) Specifically, they can optimize mechanical and thermal transfer pathways in sensors and therefore improve both sensing and stability [74]. (2) They can be used to integrate functional layers with distinct response characteristics, which allows for the construction of multifunctional sensors to detect multiple stimuli [170]. (3) They can also increase the effective contact area or triboelectric interface and significantly improve the sensitivity [81, 94]. Therefore, sandwich-structured sensors usually have excellent repeatability, reliability, operational stability, and environmental robustness [208]. Given their inherent operating mechanisms, layered structures remain nearly indispensable as a standard design for capacitive and triboelectric sensors [187].

It is important to note that these structural design strategies are not mutually exclusive and can often be effectively combined [214, 236].

**Fig. 8:**
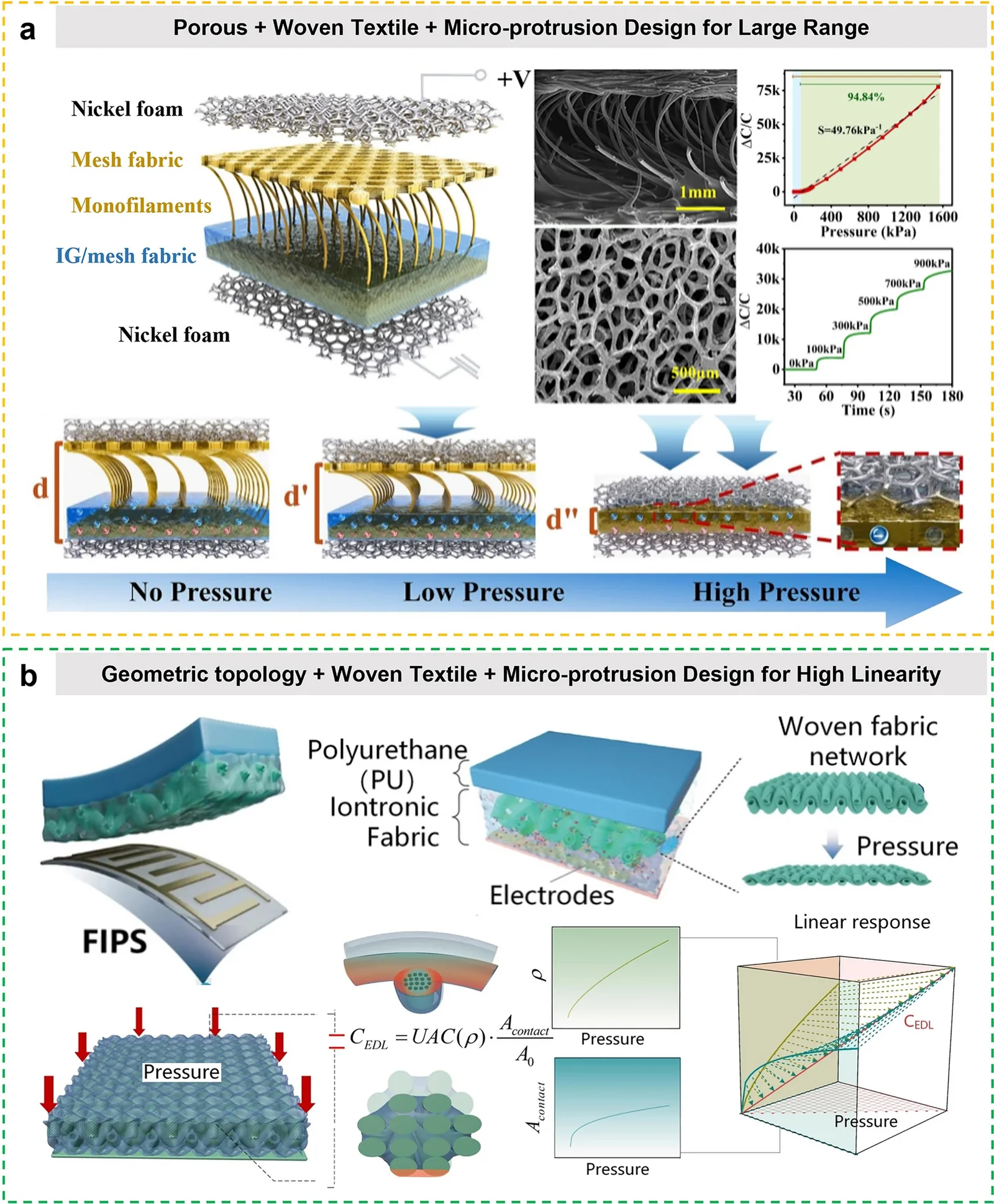
Typical example of multi-structure coupling design. **a** 3DPLE/IG capacitive pressure sensor [83] Copyright 2025, Elsevier. **b** FIPS capacitive pressure sensor [92] Copyright 2025, Springer Nature

For example, Duan, Wang et al. [83] fabricated a 3DPLE/IG capacitive pressure sensor that simultaneously incorporated a whisker-like micro-protrusion structure, a porous structure, and a sandwich structure. The whisker-like micro-protrusions provided high sensitivity at low pressure, while at higher pressures, once the micro-protrusions reached their deformation limit, the sandwich and porous structures became dominant. The large specific surface area of the porous nickel foam electrode facilitated the full utilization of the electric double-layer (EDL) capacitive sensing mechanism within the sandwich structure under high pressure. This effective coupling of these mechanisms contributed to a wide linearity of 94.84% across a 0 ~ 1550 kPa range and a sensitivity of 49.76 kPa^−1^, as illustrated in Fig. 8a.

Li, Yang et al. [92] developed a flexible capacitive pressure sensor (FIPS) featuring a multi-structure coupled design that incorporated interdigital electrodes, a textile matrix, an iono-gel composite, and a sandwich architecture (Fig. 8b). The bottom layer consisted of interdigital flexible printed electrodes to enhance signal collection efficiency. The middle functional layer consisted of a composite of a textile matrix (cotton or leather, leveraging its warp-weft woven structure to enable a regular expansion of contact area under pressure) and an iono-gel (PVA-H_3_PO_4_ or PVDF-HFP). The top layer comprised a polyurethane (PU) package. Through the synergistic dual-mechanism of the textile microstructure and the iono-gel, the sensor achieved a high sensitivity of 242 kPa^−1^, a linearity of 99.7%, and a record-breaking linear sensing factor (LSF) of 242,000 across a broad measurement range of 0 ~ 1 MPa.

Additionally, textile materials including cotton cloth [94], spandex [82], glass fiber fabric [92], nylon fabric [92], polyester fiber [112], and paper-based substrates [183] are also used as substrates or supporting structures in specific types of flexible sensors, leveraging their unique properties such as breathability, wearing comfort, or foldability.

### Innovative Structural Designs for Multifunctional, Multidimensional, Multimodal Sensors

Sensing systems develop rapidly and are used in increasing cutting-edge applications. The related research has changed from traditional single-function, single-dimensional sensing capabilities to integrated multifunctional and multidimensional sensing systems [237]. This evolution has three distinct yet interrelated concepts. Specifically, multifunctionality, which refers to the ability of a sensor to simultaneously detect multiple types of mechanical/non-mechanical quantities; multidimensionality, which denotes the ability of a sensor to resolve both the magnitude and direction of stimuli along multiple spatial or feature axes simultaneously; and multimodality, which describes the ability of a sensor to perform detection using numerous types of signals. Multimodality serves as an optional approach to achieving the other two objectives. It can fully leverage the strengths of various sensing mechanisms, offering distinct advantages. Consequently, it has garnered increasing attention in recent years.

**Fig. 9:**
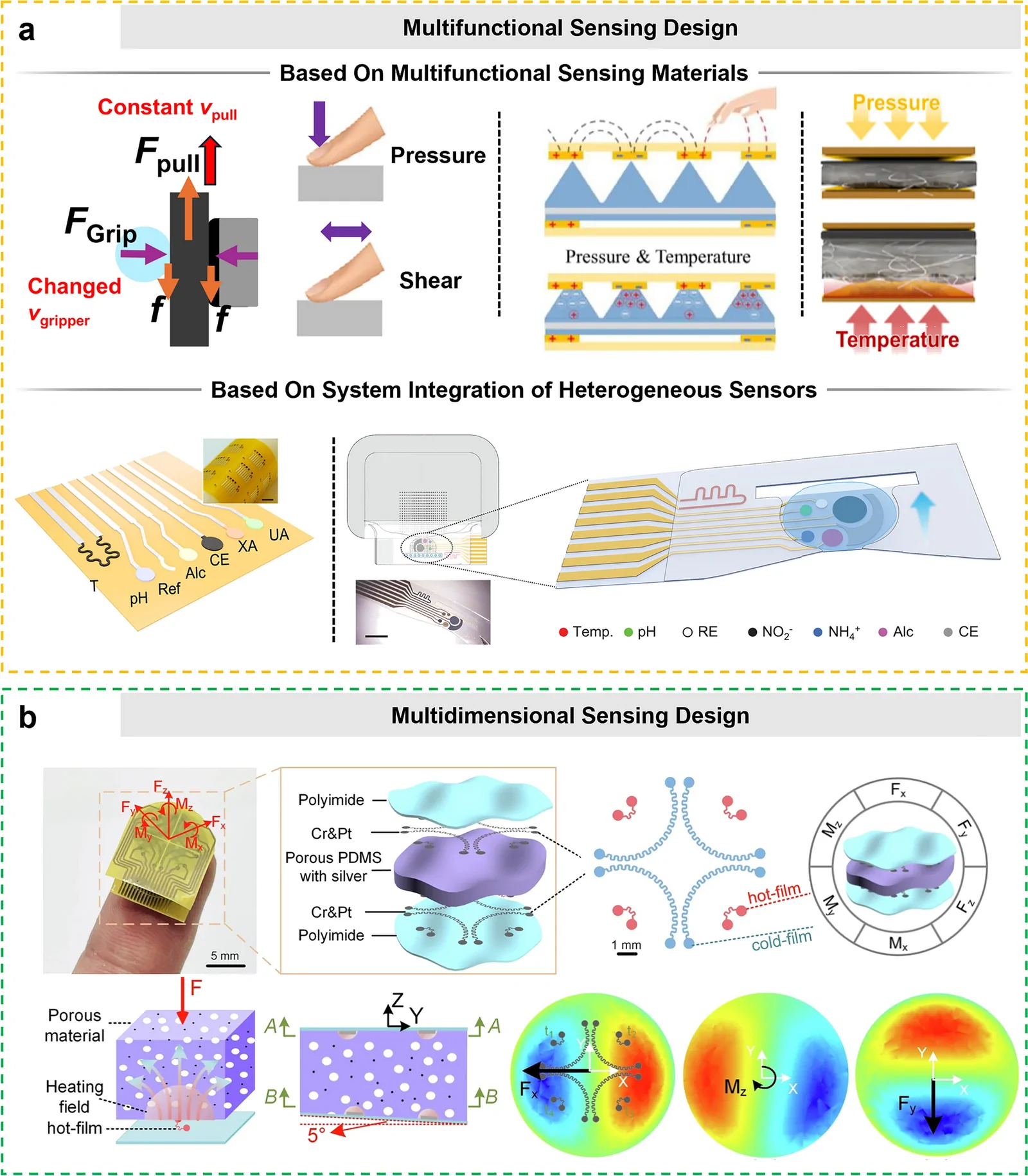
Multifunctional, multidimensional designs of flexible sensors. **a** Multifunctional sensing based on multifunctional-sensing materials [82, 91, 208], and based on the system integration of heterogeneous sensors [53, 57]. **b** Multidimensional force/moment sensor based on the mechanism of force-induced-thermal-field [43]. **a** Reproduced with permission from Ref. [82]. Copyright 2025, Springer Nature. Reproduced with permission from Ref. [91]. Copyright 2025, John Wiley and Sons. Reproduced with permission from Ref. [208]. Copyright 2025, John Wiley and Sons. Reproduced with permission from Ref. [53]. Copyright 2025, The American Association for the Advancement of Science. Reproduced with permission from Ref. [57]. Copyright 2024, The American Association for the Advancement of Science. **b** Reproduced with permission from Ref. [43]. Copyright 2025, Springer Nature

For multifunctional sensors, two primary technical strategies exist. First, leveraging multifunctional-sensing materials to enable a single sensor device to perceive multiple types of stimuli [82, 91, 118, 208]; second, constructing multifunctional-sensing systems through the system integration of heterogeneous sensors [11, 53] (strategies that integrate multiple independent sensors via a sandwich structure also fall into this category [66, 88, 219]), as shown in Fig. 9a. The choice between these strategies, along with the potential need for multimodal signal design and decoupling algorithms, depends heavily on the specific application requirements.

The realization of multidimensional sensing requires sophisticated multi-axis structural design paired with corresponding decoupling algorithms. Just as three non-coplanar coordinate axes define a three-dimensional world, multi-axis designed multi-sensor systems and their corresponding decoupling algorithms remain indispensable [221, 227].

Mao, Zhu et al. [43] designed a flexible 3D force/torque sensor based on a force-induced thermal field transduction mechanism. This sensor featured 8 pairs of Cr/Pt thermal sensing electrodes on the upper and lower surfaces of a silver-doped porous PDMS sensing layer. When mechanical loads altered the material’s thermal conductivity field, a multi-layer perceptron (MLP) neural network mapped the thermal field distribution into a six-dimensional force/torque vector, achieving a sensing accuracy exceeding 99% for forces and moments along three dimensions (6 signals in total), as shown in Fig. 9b.

### Architectural Innovations in Sensor Systems for Wider-scope and Higher-dimensional Detection

The inherent limitations in the information dimensionality of isolated sensor units constrain their perceptual capabilities. Although scientists continue to optimize the performance of individual sensors, pursuing high sensitivity, multidimensionality, and multimodality, the capacity for high-information-density perception at a macro-scale, encompassing discrete multi-point or continuous field mapping (e.g., stress/strain field distribution, temperature/concentration gradient fields, posture recognition, and substance identification), remains reliant on system-level architectural design. This is analogous to imaging. While the brightness and color of a single pixel are essential, it is the collaborative information from multiple pixels that constitutes a rich visual world. Furthermore, achieving this requires the co-development of tailored hardware and algorithms.

**Fig. 10:**
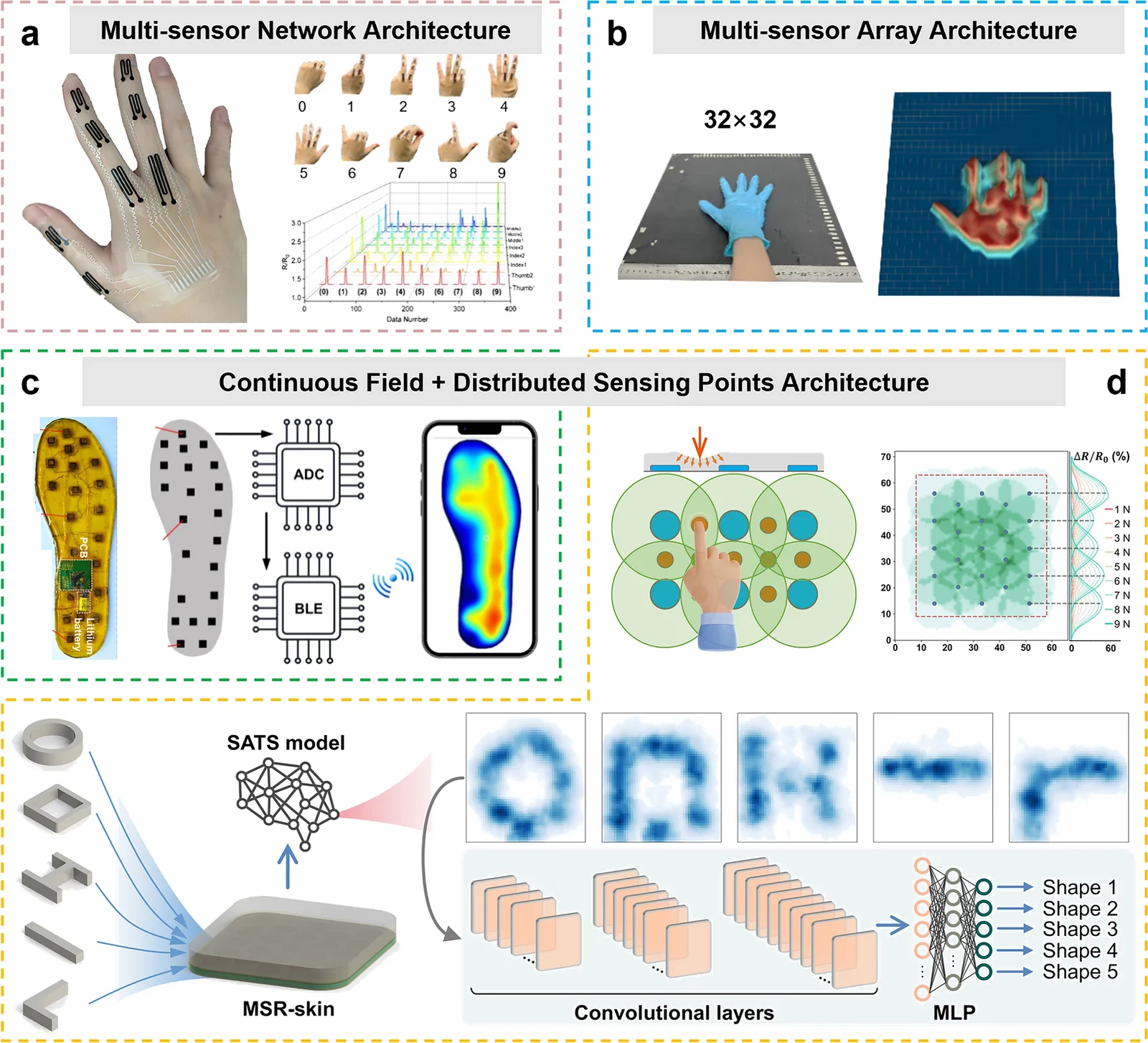
Schematic diagram of architectural designs of sensors. **a** Multi-sensor network architecture [31] Copyright 2025, John Wiley and Sons. **b** Multi-sensor array architecture [92] Copyright 2025, Springer Nature. Continuous field + distributed sensing points architecture: **c** mapping of pressure distribution using the smart insole system with a 22-sensor network [165] Copyright 2025, The American Association for the Advancement of Science, and **d** mapping of pressure distribution using the 23-sensor array system with the SATS super-resolution algorithm [210]. Copyright 2025, The American Association for the Advancement of Science

Current sensor system architectures primarily fall into three categories:

- 1. Multi-sensor network architecture. This design employs a loosely coupled network of multiple, independent sensor units, homogeneous or heterogeneous, to form a multi-point perception system. The spatial arrangement of sensors is not confined to a specific shape or contour and can be customized for the application. This exceptional flexibility allows for its adaptation to complex spatial structures and diverse sensing applications, typically including gesture recognition, posture recognition, and multi-parameter environmental detection [31, 78, 99, 205]; Lv, Xie et al. [31] designed a flexible and highly adhesive CNT/PDMS-PEG sensor based on the concept of mono-polymer-matrix substrate-matrix-package. The PDMS-PEG matrix showed a suitable elastic modulus (186 kPa), high elongation at break (> 220%), and strong adhesion strength (1.2 N cm^−1^), which allowed for conformal skin attachment without requiring adhesives or straps. The CNT/PDMS-PEG strain sensor accurately detected finger joint angles. Eight such sensors were integrated to form an 8-unit E-skin, as shown in Fig. 10a, which simultaneously monitored the flexion angles of 8 major hand joints and achieved a gesture recognition accuracy of 99.61% for hand signs representing the digits 0 ~ 9.
- 2. Multi-Sensor array architecture, illustrated in Fig. 10b, consists of a two-dimensional matrix of regularly arranged sensor units (homogeneous or heterogeneous), enabling high–spatial resolution multi-point perception while maintaining high performance at each sensing node [238]. The performance, size, quantity, and density of sensor units collectively determine the array’s sensitivity, resolution, and effective range [135, 239]. Consequently, high-performance sensor arrays place extreme demands on materials and fabrication processes [105]. Similar to high-resolution displays composed of tiny pixels, when the sensor density is sufficiently high [142, 240] (reference value in Ref. [84] was 10 units cm^−2^, equal to the density of Merkel cells in the human palm, approximately), this can achieve the high-fidelity field perception of external stimuli [241, 242]. Ensuring the stable operation and reliable signal transmission of each unit/type necessitates complex structural, hardware, and algorithmic designs. These are predominantly used in cutting-edge applications such as biomimetic dexterous hands and electronic skin for embodied intelligence [46, 125]. It is impractical to allocate enough separate I/O lines for every sensor. A time‑division multiple access (TDMA) strategy for the efficient utilization of signal lines therefore represents an inevitable choice [197, 206, 227, 243]. The individual sensors are interconnected via shared row and column signal lines, and multiplexers together with switching matrices (e.g., TMUX1134 [75], TMUX1112 [53]) are adopted to realize temporal isolation and array addressing for each sensing unit in the array, thereby significantly reducing the number of required signal lines [135, 239].
- 3. Continuous field + distributed sensing points architecture involves deploying multiple sensing points to monitor a field (e.g., stress [43], thermal [43], magnetic [51]) within a continuous material in real-time. Perception over a larger, more continuous area is achieved by detecting the influence of external stimuli on this field. Signals from individual points are then decoded based on the coupling relationship between the field and spatial distribution of the points, enabling a super-resolution reconstruction of the continuous information field rather than discrete point measurements [165, 210]. This architecture is a refined version of the former two, which means that sensing can be rapidly enhanced by using improved algorithms (e.g., interpolation [165] or super-resolution algorithms [75, 210]). This architecture’s resolution, sensitivity, and effective area depend on the properties of the sensing material, the number, density, and distribution of the sensing points, and the algorithm. The layout of these points is critical and specific patterns [43], shapes, or contours are usually used [165] to ensure reliable field-point coupling and required applications.

As shown in Fig. 10c, Wang, Lan et al. [165] constructed a highly integrated and self-powered wireless smart structure that used a resistive CNT/CB/PDMS pressure sensing composite material with a linearity of > 99.9% within the 0 ~ 225 kPa range. The system achieved self-powering via a flexible perovskite solar cell combined with a lithium-ion battery (LIB), integrating 22 sensors with a dense distribution in the toe/heel regions and two additional sensors in the arch area for flatfoot diagnosis to collect plantar pressure data. A super-resolution algorithm process was implemented using biharmonic spline interpolation. This method constructed a kernel function matrix based on the Green’s function and, after matrix inversion via Gaussian elimination, transformed the 22 discrete pressure data points into a high-resolution 150 × 200 plantar pressure distribution map. The resulting real-time pressure distribution map from the sensors, as well as real-time pressure curves from individual sensors, could subsequently be displayed on a smartphone application.

As shown in Fig. 10d, Kong, Yang et al. [210] fabricated a porous CNT/PDMS resistive pressure sensor. Specifically, they designed an end-to-end self-attention-assisted tactile super-resolution (SATS) machine-learning framework to estimate pressure distribution on the sensing surface by processing multichannel signals. The resulting multi-point super-resolution skin generated 2700 virtual tactile units from just 23 physical tactile units, achieving a super-resolution ratio of approximately 117. The average surface localization error was only 0.73 mm, comparable to the localization accuracy of a human fingertip (0.3 ~ 1 mm).

Critically, these architectures are not mutually exclusive and can be synergistically integrated.

Furthermore, when these system-level architectural sensing systems are endowed with biomimetic traits [244] (e.g., thinness, softness, stretchability, multifunctional perception, high density/accuracy) and biocompatible properties (e.g., biochemical compatibility, necessary adhesion, gas/liquid exchange, mechanical matching, self-healing [24, 37]), they constitute an E-skin system [204, 245]. This related field is detailed in our previous work [246].

To achieve this leap, a series of intertwined challenges for devices and arrays must be considered:

- 1. For material selection, in addition to the basic principles mentioned in Sects. 3.4 and 3.5, for temporary implantation scenarios, attention should also be paid to the controllable degradability of materials, which should degrade at a harmless rate and be absorbed by the body after completing their functions [232, 247];
- 2. For device structures, in addition to the basic role of protecting the device, they should also allow necessary gas/liquid exchange to adapt to bio-wearable application scenarios [197];
- 3. Safety and crosstalk issues: Crosstalk (discussed in detail in the next section) imposed on sensor signals by fluctuations in the physiological environment (e.g., inflammation, fever, or environmental changes) and the organism’s own bioelectrical signals (e.g., ECG, EMG) cannot be ignored. Consequences such as the device’s operating voltage/current harming the organism or disrupting normal physiological electrical patterns are unacceptable. Therefore, the operating voltage, operating current, and electrical frequency must be limited within safe ranges, and safe isolation between the device and the organism should be achieved through isolation layer design, differential signaling, frequency filtering, and other strategies;
- 4. Efficient communication and energy supply technologies: Complex sensing systems such as high-density arrays impose high requirements on high-speed communication and stable energy supply, and bulky communication/power supply cables or modules are undesirable in relevant working scenarios [197]. Thus, highly integrated hardware modules, high-speed wireless communication modules, and high-energy–density flexible batteries [248] are favorable. Designs including high-efficiency energy harvesting units [37] (e.g., from body motion, biochemical reactions, or radio-frequency fields), passive designs [247, 249–251], flexible hybrid electronics [197, 252], and in-sensor/near-sensor computing [253, 254] are increasingly preferred;
- 5. The pursuit of high-precision heterogeneous integration technology remains consistent: Components with distinct functions (e.g., physical sensing, chemical detection, signal processing, communication) and highly different material systems should be integrated onto the same flexible substrate with high density and high yield, while ensuring reliable interconnection under mechanical deformation.

### Anti-Crosstalk Designs for High-Fidelity Sensor Systems

The realization of these advanced sensing capabilities has greatly expanded the application fields of high-performance sensors, but it also brings a critical challenge: signal crosstalk. Severe crosstalk leads to a low signal-to-noise ratio (SNR) in the sensor system, and even causes truly useful signals to be submerged in crosstalk components, which greatly affects the effective analysis of sensor data. The academic community has established a systematic solution to this problem [73]. Since crosstalk signals originate from two stages — signal generation (flexible devices) and signal transmission (hardware circuits and communication)—targeted design of these two stages, combined with algorithms in the final signal processing step, can ultimately achieve optimal suppression of crosstalk:

(i) *Suppression at the Source*: the physical properties of flexible devices are the main inducements of multi-source crosstalk. Sources include undesired electrical signal variations of electrodes during device deformation, responses of sensing materials to unintended stimuli, mutual interference between multiple sensing material regions, and performance drift of materials caused by environmental changes. Another inducement is electrical interference such as power supply ripples and parasitic signals during current transmission. Their suppression requires co-design from the perspectives of material selection, structural design, packaging design, and hardware schemes of the sensing devices. The main strategies include: using strain-insensitive electrodes (e.g., the liquid metal mentioned earlier), reducing crosstalk paths between functional regions via regional segmentation and physical isolation [255], applying local enhanced packaging to interference-sensitive materials, constructing independent signal readout channels for each functional region, adopting high-quality power supplies such as batteries, and equipping dedicated filter circuits, so as to suppress interference coupling at the source [66, 75, 91, 243].

**Fig. 11:**
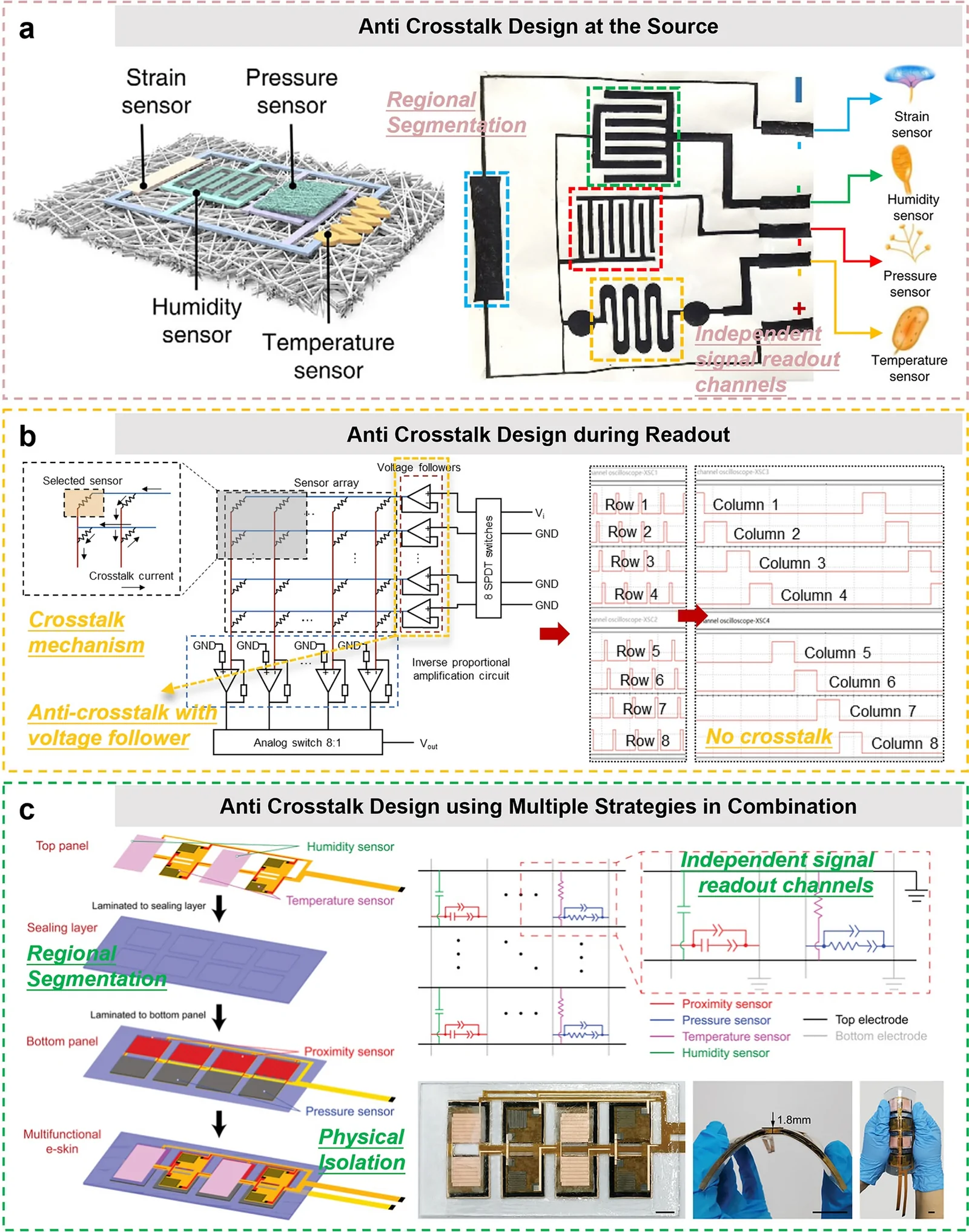
Anti-crosstalk designs of flexible sensors. **a** Multifunctional integration and the anti-crosstalk design of the SMED sensor [143] Copyright 2025, John Wiley and Sons. **b** Crosstalk in sensor arrays and anti-crosstalk design based on voltage followers [76] Copyright 2024, The American Association for the Advancement of Science. **c** Multifunctional integration, device architecture, and the anti-crosstalk design of the dual-modal, quad-functional E-skin [89] Copyright 2023, John Wiley and Sons

As shown in Fig. 11a, Zheng, Cao et al. [143] designed a quad-functional, uni-modal skin-like multifunctional sensor termed SMED based on MXene/PEDOT:PSS multifunctional-sensing materials and thermoplastic polyurethane (TPU) nanofiber mat. Employing a system integration strategy for heterogeneous sensors, this system integrated four functions: strain, pressure, temperature, and humidity, all based on resistive sensing mechanisms. Through designs incorporating functional zoning, physical isolation, and independent signal channels, excellent anti-crosstalk performance was achieved. When humidity changed (43% ~ 75% RH), the resistances of the temperature and strain sensors showed no significant variation, and when pressure was applied (0 ~ 38.3 kPa), the resistances of the temperature, humidity, and strain sensors remained stable. Only the corresponding sensing unit responded, ensuring independence and reliability across multiple functions and enabling stable applications such as ECG monitoring and gesture recognition.

Although complete elimination of all crosstalk is difficult to achieve, the magnitude can be greatly reduced at the source, laying a foundation for subsequent signal processing. If hardware-based suppression is insufficient, algorithmic innovation can be used for compensation. However, these strategies often require compromises in device structure, layout density, and miniaturization level, leading to increased overall complexity, and offer low flexibility for post-adjustment once the design and fabrication are finalized.

(ii) Suppression during readout: this type of crosstalk mainly occurs in large-scale sensor arrays and originates from the non-ideal characteristics of hardware and circuits. Sensors are connected via shared row/column signal lines. When a sensor unit is selected, its readout signal is disturbed by several factors [76] include resistive coupling (leakage current caused by incomplete turn-off of unselected sensor paths, which superimposes with the signal current of the selected path), capacitive coupling (parasitic capacitance between unselected sensors and their signal lines and the selected path, leading to deviation of the readout voltage), as well as other non-significant factors such as electromagnetic interference.

The basic suppression strategies are impedance conversion and buffer isolation. For example, a voltage follower with high input impedance (e.g., AD8574 [76], ADA4691-4 [135]) is connected to the post-stage of the sensor unit. Its high input impedance can effectively reduce the load on the front-end sensor and avoid signal attenuation; its low output impedance stabilizes the node voltage and suppresses interference currents caused by parasitic capacitive coupling, thus ensuring intact signal transmission over long buses [243], as shown in Fig. 11b.

This crosstalk suppression method provides critical signal integrity assurance for complex sensor array systems with a large number of sensing units, and has thus been widely adopted in increasingly large-scale sensor groups/arrays. The cost is increased complexity and power consumption of the hardware module, as well as a limitation on the maximum signal acquisition rate of the system (which is inevitably lower than the theoretical operating speed of devices such as MCUs, operational amplifiers, and multiplexers).

(iii) *Decoupling via Algorithms*: residual crosstalk may still exist after intervention by the above two stages, which can be further filtered out by algorithms. Advanced algorithms can separate the contributions from different sources in the superimposed signals and achieve remarkable effects in extracting useful signals (this topic will be discussed in detail in Sect. 6), thereby correcting crosstalk that is difficult to completely eliminate only by material, structural, and hardware design. The main drawback is the high demand for high-quality datasets and computing power; the more irregular the crosstalk, the higher the cost required.

These three schemes are not mutually exclusive; instead, they cooperate with each other to jointly achieve excellent and high-fidelity performance of the sensor.

Ge, Zhang et al. [89] proposed a quad-functional, dual-modal electronic skin (E-skin) integrating different sensing materials and structures, as shown in Fig. 11c. This E-skin could simultaneously and accurately detect four signals: pressure (capacitive, 0 ~ 30 N, sensitivity 16.34 N^−1^, CB/PDMS composite), proximity (capacitive, 0 ~ 100 mm, sensitivity 0.72 mm^−1^, CB/PDMS composite), humidity (capacitive, 20% ~ 70% RH, sensitivity 15.2 pF/%RH, PVDF/PVA/LiCl composite film, sensitive only to humidity), and temperature (resistive, 20 ~ 120 °C, sensitivity 0.0032 °C^−1^, serpentine nickel film, sensitive only to temperature). At the physical level, a sandwich structure, dedicated independent sensing units for each function, targeted package/isolation, and independent signal lines were employed to reduce intrinsic crosstalk. The sandwich structure design mitigated the impact of ambient humidity on the capacitive signals for pressure and proximity. The PDMS package material provided further moisture isolation for the temperature, proximity, and pressure sensing units. The design of mutually independent signal lines for different signal types prevented electrical crosstalk. At the algorithmic information, a quantitative temperature-compensation model was developed to reduce signal crosstalk further. By fitting a large dataset, a cubic polynomial relationship between temperature and the proximity sensor’s capacitance was derived, eliminating temperature crosstalk with pressure and proximity sensing. Ultimately, the maximum cross-coupling error values for proximity, pressure, temperature, and relative humidity were 1.96%, 1.08%, 2.65%, and 1.64%, respectively. The sensing system was successfully demonstrated on a commercial robot (AUBO i − 10), confirming its excellent performance in enhancing the robot’s capability for complex tasks, including distance tracking, pressure tracking, and active safety control.

Looking forward, sensors will develop along the path of co-optimizing materials, structure, hardware, and algorithms toward the direction of integrated multifunctionality and multidimensionality. Intelligent sensing systems with high spatial resolution, high-dimensional resolution, and low crosstalk will also develop based on the advances in novel, sensitive materials and artificial intelligence algorithms, which will play an increasingly vital role in the applications including dexterous robotic manipulation, wearable health monitoring, and natural human–computer interactions.

## Hardware

The sensed signals, such as temperature, pressure, light intensity, biopotentials, or compound concentration have to be converted into digital signals to be recognized and processed by digital systems in a precise, fast, and reliable way. This critical conversion is accomplished via dedicated hardware systems.

### Core Stages of Sensor Hardware Systems

**Fig. 12:**
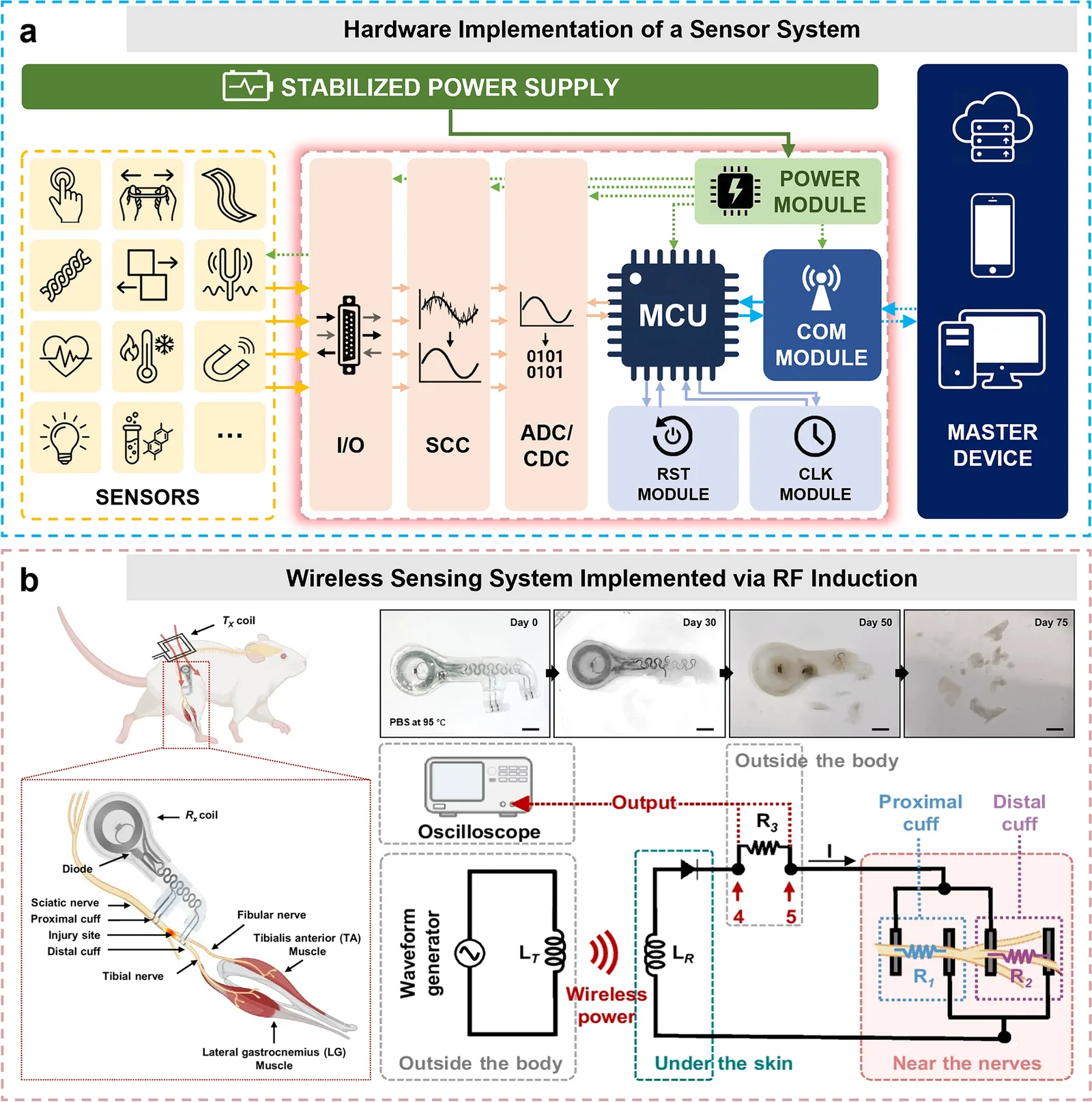
**a** Typical hardware implementation of a sensor system. **b** Schematic diagram of a wireless sensing system implemented via RF induction [27]. I/O: input/output interfaces, SCC: signal conditioning circuit, ADC: analog-to-digital converter, CDC: capacitance-to-digital converter, RST: reset module, CLK: clock module, COM module: communication module. **b** Reproduced with permission from Ref. [27]. Copyright 2025, Springer Nature

As illustrated in Fig. 12a, a typical hardware implementation for a sensor system comprises the following core stages [180, 255, 256]:

- 1. Forming a closed front-end circuit that connects the electrode(s) of one or multiple sensors to analog signal processing circuitry, excitation circuitry (if required), and a power supply (regulated power source or battery), converting the original stimulus into an electrical signal;
- 2. Signal conditioning circuit (SCC, including but not limited to amplification and filtering [133]);
- 3. Converting the analog signal into a digital signal using a single- or multichannel analog-to-digital converter (ADC) or capacitance-to-digital converter (CDC [84, 89]);
- 4. Preliminary signal processing by a microcontroller (MCU);
- 5. High-speed data transmission via a communication interface (UART [65], RS232 [209], RS485 [89], Bluetooth [256], Wi-Fi [211], RF [257]);
- 6. Reception by a master device;
- 7. Algorithmic processing.

The signals generated by sensors are diverse, while back-end information processing circuits must operate within specific amplitude and frequency ranges. Furthermore, environmental noise remains inevitable. Therefore, *signal conditioning circuits*, tailored explicitly to the sensor and responsible for amplification and filtering, are indispensable.

Except for a few applications with relatively simple functionality [94, 95], most advanced sensor systems and their measurement principles require the inclusion of an *analog-to-digital converter* (ADC, for converting analog signals to digital form) and a *microcontroller* (MCU, responsible for preliminary signal processing, embedded system control, and communication management).

In terms of *power supply*, although triboelectric and piezoelectric sensors can harvest energy, a power source must still be used to ensure the stable operation of their back-end hardware circuits when these sensors are not actively triggered [93–95]. In some systems with simpler functionality and structure, *radio-frequency (RF) communication technologies* [247, 250, 251] and magnetoelectric wireless power transfer technologies [249] can enable an entirely passive device design, as shown in Fig. 12b.

### Signal Conversion Principles in Sensor Circuits

For resistive signals, common detection principles include the voltage-divider method [214], the Wheatstone bridge [257], the impedance spectroscopy technique [69], and the operational amplifier (op-amp) conversion principle [239]. The voltage-divider method has been widely adopted in wearable and portable devices where moderate accuracy suffices [243, 258], due to its simple structure and low cost. However, it requires compensation in subsequent calibration algorithms caused by its inherent systematic errors [31, 208]. In Wheatstone bridge, a differential measurement structure is used that effectively suppresses common-mode interference including temperature drift, and therefore significantly enhances sensitivity to minute resistance variations, which allows for its high-precision applications, such as slight strain and temperature measurement [11]. In impedance spectroscopy, an AC excitation is applied to the sensor. It then analyzes the response spectrum to obtain impedance magnitude and phase information, which has special advantages in characterizing complex impedance behavior while also rejecting environmental noise [69]. With op-amp conversion principle, sensors are directly incorporated into the amplifier circuit as feedback or input elements, which ensures efficient resistance-to-voltage conversion. This approach has a compact footprint which facilitates multiplexing and control, thus rendering it well-suited for large-scale, highly integrated sensor arrays [239].

For capacitive signals, the detection methods include AC impedance analysis based on LCR circuits [259, 260], frequency-shift detection using LCR resonant circuits [80, 261], AC excitation capacitance-to-voltage conversion based on oscillator circuits [87], capacitance-to-digital conversion using dedicated CDC integrated circuits [92], and capacitance-to-frequency conversion using multivibrators [85], all of which enable active and real-time monitoring.

For triboelectric and piezoelectric signals, the hardware system is primarily used to acquire the weak charges induced by the sensor, to be measured using the charge–voltage relationship (Q = C × V). In the first stage it is critical to have a charge amplifier or voltage amplifier with extremely high input impedance [102], which can convert the charge signal into a stable voltage signal while also suppressing interference. Subsequently, signal conditioning and filtering may be further required to improve the signal-to-noise ratio and thus enable reliable detection.

Beyond these primary sensing mechanisms, there are other mechanisms suited for specific signal categories. For example, biopotentials (e.g., electrocardiogram, ECG, 0.1 ~ 5 mV; electromyogram, EMG, *μ*V-to-mV range [79]), need high-gain amplifiers and precision filter circuits, and also high-resolution ADC, with the prevailing acquisition method being differential potential measurement [168]. For measuring the impedance of biological tissues, which exhibits both resistive and capacitive components and is also very weak, methods such as the excitation-response impedance analysis principle [47] and the active AC impedance analysis principle [121, 167] are used. For detecting specific molecules such as ethanol, lactate, uric acid, and purines, the surface enhanced Raman spectroscopy (SERS) principle [59] is an alternative to the (enzyme/-) amperometric detection principle [60, 62]. For pH sensing, the potentiometric measurement principle based on the Nernst equation has been typically employed [53].

### Parameter Selection Based on Sensor System Application Scenarios

Key parameters for the hardware component of a sensor system include response speed, signal-to-noise ratio (SNR), power consumption, integration density, ADC resolution, ADC sampling rate, communication scheme, on-chip computing capability, cost, and reliability. The response speed (contributed by the communication scheme and on-chip computing capability) determines the system’s real-time capability, with the SNR dictating its signal detection/analysis capability and interference immunity. ADC resolution and sampling rate assess its ability to detect weak signals. Power consumption directly affects the operation lifetime, and the integration density and cost influence its feasibility and deployment scope.

The specific requirements for these parameters highly depend on specific applications. For example, response speed or power consumption are typically not highly required in sensing applications involving non-transient phenomena with access to stable power, such as monitoring compound density fields [53] or ambient temperature fields [262].

### Implementation and Integration Techniques in Advanced Sensor Systems

For specific implementation approaches, the most straightforward method is to use an MCU with an integrated ADC [232] or to combine a high-precision discrete ADC/CDC module with an MCU [258], together with external *discrete electrical components* to interface with the sensor. This method has the advantages of simplicity, rapid development, and low cost. However, it has the disadvantages of low integration density, poor stability and reliability, a large footprint occupied by discrete components (e.g., resistors, filter circuits), and susceptibility of the signal to environmental interferences. Notably, related research usually investigate more on the intrinsic properties of the sensors rather than on the systematic optimization of the entire sensing system.

**Fig. 13:**
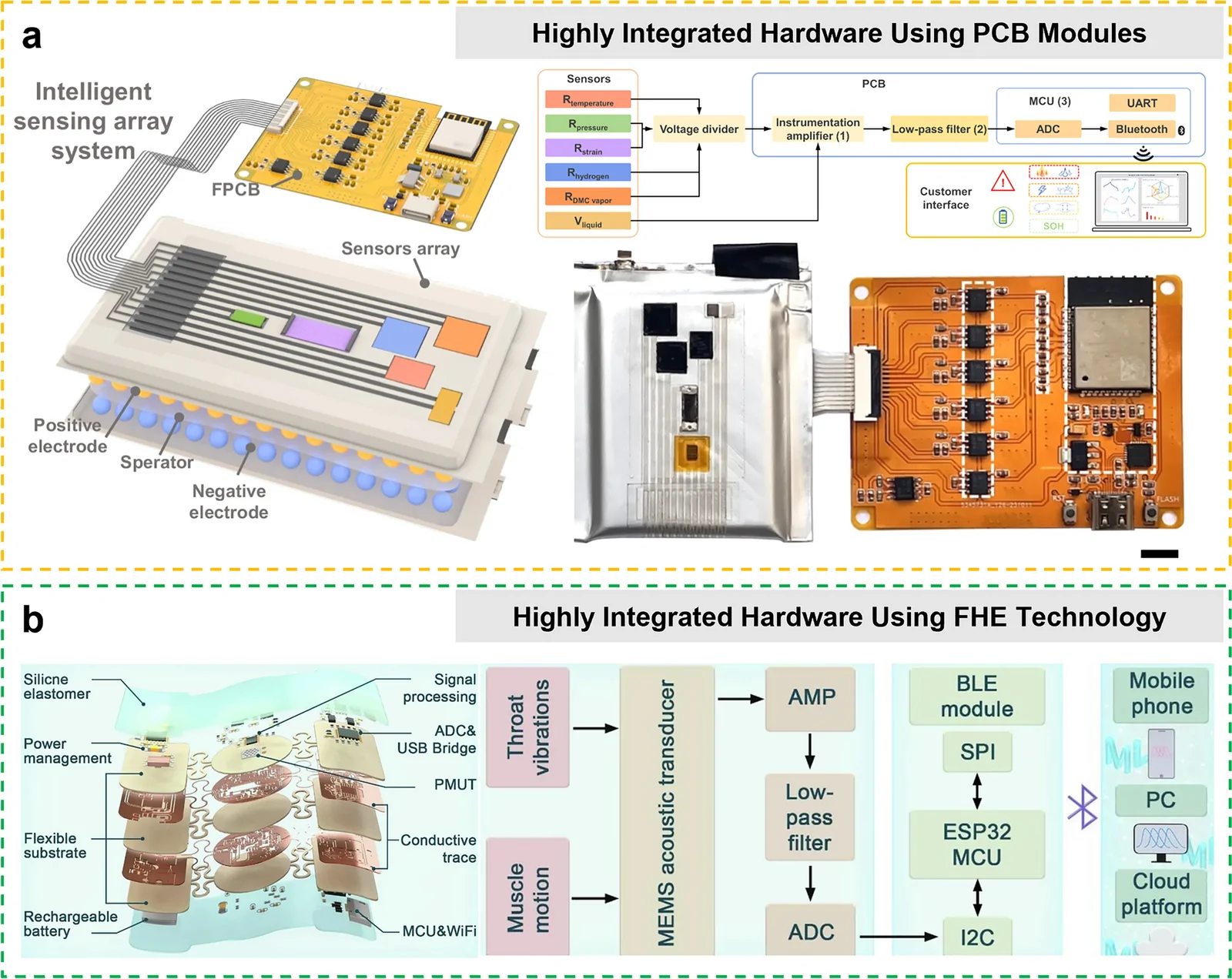
**a** Schematic diagram of a hardware architecture and system composition for the IISAS battery health sensing system [262] Copyright 2025, Springer Nature. **b** Schematic diagram of the hardware architecture and system composition for the PMUT array sensing system [102] Copyright 2025, Springer Nature

For high-fidelity application scenarios such as spatially sensitive, implantable, or wearable devices, some research teams have directly designed dedicated, highly integrated *printed circuit board (PCB) modules* [169]. Sun, Rong et al. [262] fabricated a fully printed highly integrated multifunctional sensor array system (IISAS) using laser etching and nanofabrication processes, as shown in Fig. 13a. This system integrated six types of sensors: temperature (20 ~ 100 C, sensitivity 0.42% °C^−1^, response time 9 s), pressure (25.0 ~ 500.0 kPa, response/recovery time 0.9 s), strain (0.1 ~ 1.0%, response/recovery time 0.3 s), H_2_ gas (1 ~ 2500 ppm), DMC vapor (10 ~ 2000 ppm), and electrolyte leakage (detection threshold 0.1 *μ*L). By integrating components such as voltage-divider resistors, AD8226 amplifiers, RC low-pass filters, and an ESP32 MCU chip onto a FPC module, it can be embedded in the packaging foil of a LIB to create a real-time health-monitoring system for LIBs. This integration added only 49 mg in weight and 16.844 mm^3^ in volume, with minimal impact on the battery’s electrochemical performance. The system was found to be adaptable to different battery types. Coupled with a CNN-LSTM hybrid deep learning model, it enabled state-of-health (SOH) estimation and real-time diagnosis and early warning of faults such as over-discharging/charging, low-temperature/high-rate Li-plating, internal short circuits, breakage, or thermal abuse. Its effectiveness has been validated in practical applications such as unmanned aerial vehicles (operating under 9.7C discharge rates).

A further step in integration employs *flexible hybrid electronics (FHE) technology* to realize a flex-on-chip solution [256, 263], achieving optimal performance and miniaturization [252, 264]. As shown in Fig. 13b, Liu, Li et al. [102] fabricated a 19-element Mo/Sc-AlN/Mo piezoelectric sensor array (PMUT) with a hexagonal arrangement and a compact size of only 3.5 × 3.5 mm^2^. This PMUT was then integrated with an ESP32 MCU chip, an ADS78223 ADC, a lithium-ion battery, and other electrical components onto a single FPC. The assembly was packaged using flexible materials such as Ecoflex and 3 M 9907 T medical adhesive to form a flexible laryngeal speech recognition system. Combined with a transfer-learned ResNet model, the system achieved high recognition accuracy of 99.5% for phonemes and 99.8% for daily sentences, offering advantages of small size, light weight, low-power consumption, and excellent recognition capability.

### Near-Sensor Computing & In-Sensor Computing

**Fig. 14:**
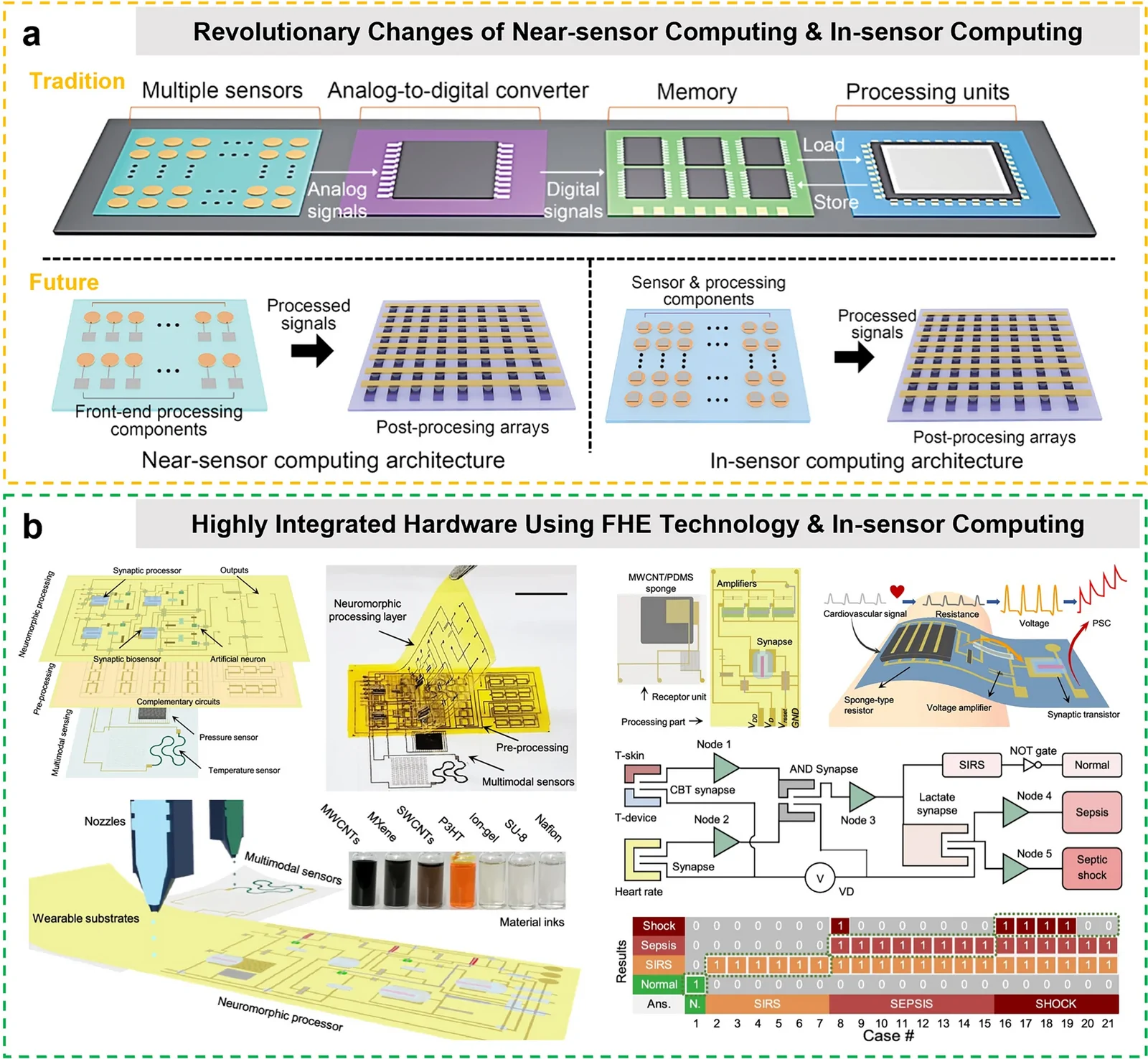
**a** Schematic diagram illustrating the trend toward integrated sensing and neuromorphic computing [265] Copyright 2025, John Wiley and Sons. **b** Schematic diagram of the system composition and operational logic of the chip-less CSPINS integrated sensing-neuromorphic computing system [160] Copyright 2025, Springer Nature

A cutting-edge, highly integrated approach involves integrating neuromorphic processing circuits with sensors into a unified, co-designed system [253, 254] (*near-sensor computing & in-sensor computing*), as depicted in Fig. 14a. In this configuration, the neuromorphic processing circuit fully assumes the functions of the SCC, ADC, and MCU mentioned previously [170, 253, 265], while also eliminating most of the communication chain. A power supply serves as the primary external requirement of the relevant system. Compared to the implementation methods mentioned earlier, this approach demonstrates significant advantages in terms of real-time performance and power consumption [254].

As shown in Fig. 14b, Choi, Gao et al. [160] developed a fully inkjet-printed, chip-less wearable flexible sensing system (CSPINS), in which flexible neuromorphic computing devices can replace traditional MCUs to enable local real-time health monitoring and decision-making. This system comprises four layers: a multifunctional sensor layer, a complementary circuit amplification layer, a neuromorphic processing layer, and a customized hardware neural network layer. These sensors include two biomarker sensors (lactate and glucose), two temperature sensors, and one heart rate sensor, for a total of five multimodal sensing channels. The complementary circuit amplification layer amplifies and denoises these five signal channels. The neuromorphic processing layer can convert the 5-channel multimodal signals into standardized synaptic currents. The hardware neural network layer (essentially a hardware-implemented medical algorithm based on predefined logic, consisting of 4 artificial synapses and 5 synaptic node circuits, each containing one Au/Nafion/Ag memristor and two MWCNT resistors) implements the physical neural network to perform local decision-making. All components were fabricated on a PI/PET flexible substrate using inkjet printing, enabling synchronous monitoring of multimodal physicochemical biomarkers (lactate 0 ~ 8 mM, glucose 0 ~ 8 mM, heart rate 55 ~ 160 BPM, core body temperature 30 ~ 40 °C) without rigid chips. This system can achieve a hierarchical diagnostic accuracy of 84.4% for differentiating healthy individuals, SIRS patients, septic patients, and septic shock patients (including validation against 11 non-inflammatory cases) and offering significantly lower power consumption (synaptic operation power of only 37.6 *μ*W, full system power of 5.3 mW), providing a new solution for real-time, portable, low-cost wearable medical diagnostic technologies.

Currently, neuromorphic computing technology is still in its early stages of development. The computational capacity of associated circuits falls significantly short of that of traditional von Neumann architecture hardware, as exemplified by the aforementioned ADCs and various master devices. Consequently, these designs remain confined to simple functional applications for now. Several representative hardware schemes are summarized in Table 6.

**Table 6:** Comparative analysis of circuit architectures of some representative sensors

Modal	Detection principle	Scenarios	Power	Signal conditioning circuit (SCC)	Signal dimension	ADC	MCU	Communication	Reaction rate (Hz or ms)	Power consumption (mW)	Ref
Resistive	Wheatstone bridge	Force/temperature detection	LIB	Two-level operational amplifier (OPA)	5-channel (2 for temperature, 3 for strain at least)	12-bit (in MCU)	–	Frequency shift keying (FSK) communication	0.5 Hz 0.033 Hz	~ 21.7 mW	[11]
		*Single-point temperature identification	LIB	Amplifier + low-pass filter circuit	1-channel	12-bit (in MCU)	ESP32	Bluetooth	~ 10 s	~ 150 mW	[127]
	Voltage dividing resistance	Gesture recognition	5 V	–	8-channel	10-Bit (in MCU)	ATmega328	UART-TTL	40 Hz	0.25 mW	[31]
		22-point pressure identification	LIB	–	22-channel	12-bit (in MCU)	AVR128DA64	Bluetooth	32 Hz	10.7 mW	[165]
		*6 functions, 6-channel sensing	LIB	AD8226 amplifier + RC low-pass filter circuit	7-channel	12-bit (in MCU)	ESP32-WROOM-32	Bluetooth	0.1 Hz	~ 100 mW	[262]
	Operational amplifier conversion	1152-point hand pressure field (24 × 48) identification	–	Operational amplifier	48-channel (6 ADCs)	12-bit (AD7928)	ESP32	UART-TTL	13 Hz	–	[135]
		548-point hand pressure field (32 × 32) recognition	–	Operational amplifier	1-channel Time-division multiplexing via 32 SPDT switches and a 32:1 analog MUX	10-bit (in MCU)	Arduino nano	UART-TTL	7.3 Hz (~ 137 ms)	–	[239]
Capacitive	AC impedance analysis	*Single-point pressure identification	–	Desktop LCR meter (TH2817B) + signal conditioning unit (M162LCR)	1-channel	16-bit (in MCU)	STM32H750	RS485	–	–	[26]
	Capacitance digital conversion	*Single-point pressure identification	LIB	FDC1004 CDC	1-channel	16-bit (in CDC)	nRF52832 Soc	Bluetooth (in Soc)	~ 10 ms	3.23 mW	[147]
Triboelectric	Direct voltage amplification	*Shear force identification	LIB	OPA333 amplifier + voltage follower	1-channel	–	–	–	28.7 ~ 39.8 ms	–	[82]
	Full bridge rectifier circuit	Power supply for small electrical appliances (468 leds/1 clock)	A capacitor, no other power	Full bridge rectifier circuit	1-channel	Not applicable	Not applicable	Not applicable	Almost real time	~ 0	[94]
	Charge-to-voltage conversion	Voiceprint vibration detection	LIB	JFET amplifier + low-pass filter circuit	19-channel	16-bit (ADS78223)	ESP32	Bluetooth	360 ms	Standby: ~ 150 mW Work: ~ 720 mW	[102]
Amperometric	Principle of enzymatic amperometric	*Two point biomarker detection	–	Complementary amplification circuit + neuromorphic modulation circuit	2-channel	Local processing of neural morphological operation circuit	–	–	Very low	5.3 mW in total	[160]
		*Body fluid detection	LIB	TLV9004 amplifier + low-pass filter circuit	3-channel	12-bit (in MCU)	STM32WB5M	Bluetooth (in MCU)	–	–	[53]

^*^this is a multimodal sensor. '-' denotes that corresponding parameters are not mentioned in the original work. All other performance data in this table are as reported in the original literature

## Algorithm

Following hardware processing, sensor signals are ultimately transmitted to master devices (e.g., PCs, smartphones, servers, or cloud platforms [266]). At this stage, algorithms play a critical role as the final link in the entire sensor system chain, directly determining the system’s ultimate performance and compensating for non-idealities introduced in upstream stages [267]. On this basis, advanced applications such as data visualization, sophisticated control strategies, and human–computer interaction (e.g., limb motion detection with remote control [47] and robotic multimodal tactile perception [118]) become feasible.

By technologies, algorithms can be categorized into mathematical model-based algorithms and machine-learning algorithms, and, by function, into decoupling (or calibration) algorithms and interpretation algorithms. Among these, mathematical model algorithms rely on the inversion of mechanical models based on physical principles, whereas machine-learning algorithms are data-driven; the latter demonstrates particularly outstanding performance in handling strong nonlinear coupling.

### Signal Preprocessing and Algorithm Design

Typically, raw digital signals undergo a series of preprocessing steps to computationally enhance their quality prior to algorithmic analysis, a process entirely distinct from the physical signal enhancement methods discussed in previous sections.

These mathematical operations generally involve the following: *digital filtering*, which retains frequency bands of interest while suppressing out-of-band interference [99, 101]; *algorithmic denoising*, which mitigates residual random noise and anomalous disturbances to reconstruct a high-fidelity waveform [268]; *domain transformation*, which mathematically maps the signal into a feature space optimal for information extraction [256]; and *time-domain segmentation* (e.g., using fixed-length frames or sliding windows), which partitions the continuous digital data into discrete segments to capture temporal dynamics and ensure dimensional compatibility with downstream processing units [123].

### Defect Compensation

During the algorithm preparation stage, dataset quality and algorithm generalization capability are two key factors that deserve full attention. They determine the defect compensation ability of the algorithm, and even the actual applicable range of flexible sensors.

First, data collection is of critical importance. For both signal decoupling tasks and sensing interpretation tasks, high-quality datasets—with sufficient volume, accurate annotation, and coverage of as many operating conditions as possible—serve as an essential prerequisite for algorithms to achieve satisfactory performance, and directly dictate the upper limit of the performance boundary that the entire sensing system can ultimately reach.

Second, the generalization capability of the algorithm determines the ultimate applicable scope of the sensing system. Most sensing systems are developed in laboratory environments; however, actual service conditions are highly diverse and variable, making it impossible to cover all application scenarios from the outset. Significant differences often exist in the physiological characteristics, living habits, and wearing modes of different users. The temperature–humidity ranges and mechanical deformation laws under distinct environments also vary considerably. Furthermore, performance degradation caused by material aging during long-term operation can lead to a drastic drop in the performance of models trained in the source domain (e.g., controlled laboratory environments) when deployed in the target domain (new users or new scenarios), resulting in pronounced drift issues in flexible sensors.

Methods to address these issues include the construction of diverse datasets, transfer learning, domain adaptation methods, and meta-learning. The first approach recommends initially accounting for as many scenarios as possible and encourages the establishment of datasets covering multiple conditions so as to enhance the model’s generalization ability. The transfer learning allows for continuous and adaptive supplementary fine-tuning (training) of pre-existing algorithms on new datasets and under unforeseen conditions. The domain adaptation method allows for continuous and online adaptation of the algorithms to long-term performance degradation of the sensors (e.g., aging, hysteresis). The meta-learning allows for fast adaptation to new applications of the models. In addition, the generalization should be verified on cross-dataset (e.g., testing across different populations and environments) rather than solely on the partitioning of a single dataset.

### Decoupling Algorithms

Decoupling algorithms disentangle and calibrate sensor signals to recover the actual physical quantities, a practice that is nearly indispensable in sensor systems. The anti-crosstalk algorithms mentioned above essentially fall into this category. In practice, after signals are generated by flexible devices and collected and transmitted by the hardware system, the signals received by the back-end master device are highly complex mixtures. These composite signals generally incorporate four components: (1) the target sensing signal (the most critical component, which may involve multiple functions, dimensions, or modalities); (2) intrinsic sensor artifacts (e.g., nonlinearity and hysteresis, which cannot be entirely eliminated despite the hardware-level optimizations discussed in previous sections); (3) crosstalk (as previously addressed); and (4) environmental clutter and baseline drift (caused by material aging, electrical interference, or variations in ambient factors like temperature, humidity, and pressure) [208].

Relying on the collected data (including input features and ground-truth labels), decoupling algorithms serve to extract the specific target signals (which may cover multiple functions, dimensions)—as defined by these labels—from such complex signal mixtures. The lower the relative intensity of the sensing signal within the composite signal, the more challenging the development of decoupling algorithms. The suppression of crosstalk discussed earlier is, in essence, a preventive measure against this consequence.

*Decoupling algorithms based on mathematical models* rely on specific sensor structures and layouts [112]. This category of methods establishes explicit mathematical fitting models through derivations based on first-principles mechanical models, finite-element simulations, and experimental data calibration, to achieve high-precision sensing [11, 89]. For sensor systems operating within their linear response range, decoupling can be achieved using linear equations fitted via low-complexity least-squares methods [167], offering the advantages of simple processing and low computational complexity [78, 147, 239].

**Fig. 15:**
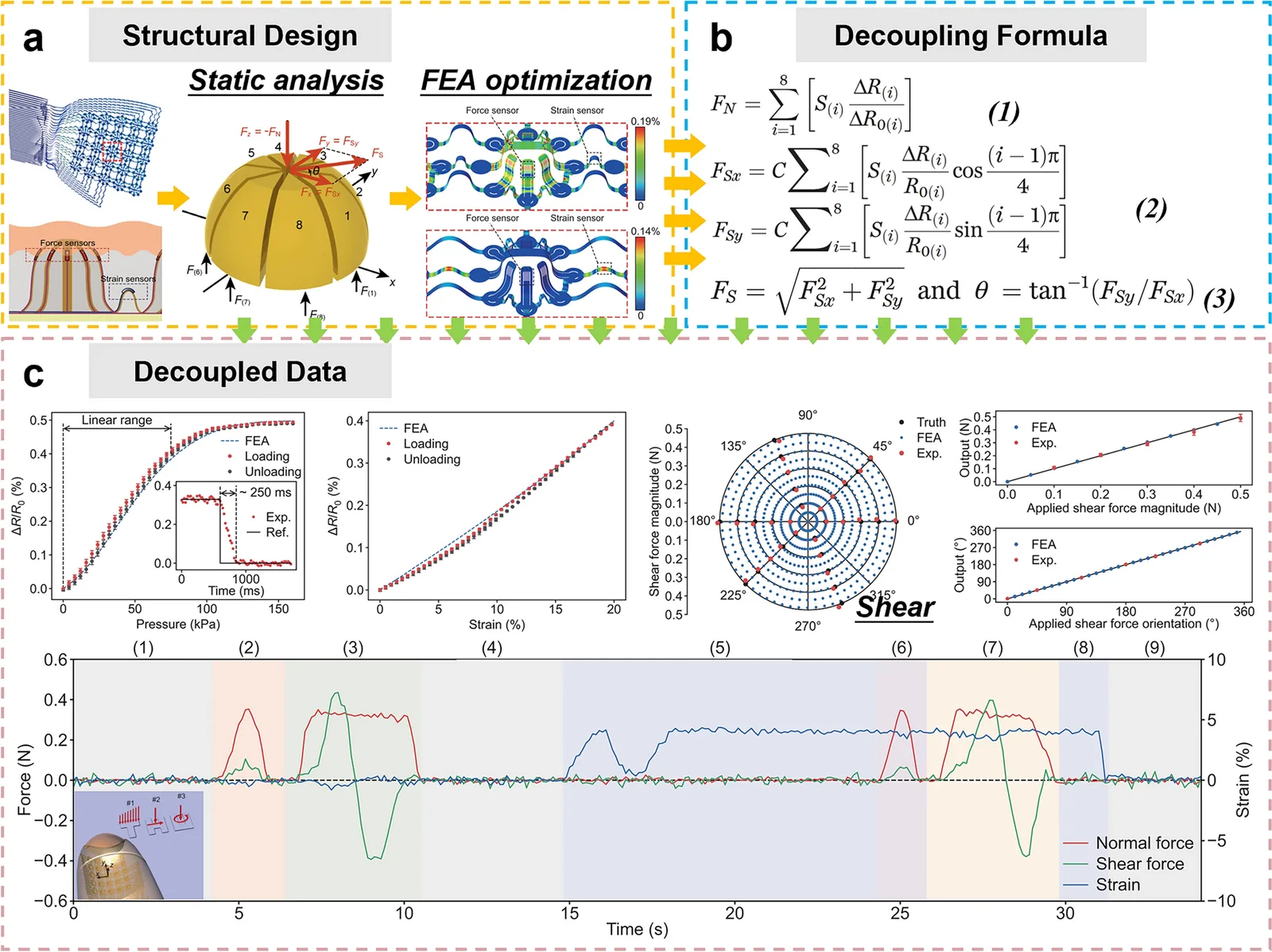
Schematic diagram of the mathematical decoupling process (pressure, strain and shear force) for the octopus-inspired, cage-like structured three-dimensional resistive flexible sensor array.** a** Structural design. **b** Decoupling formula. **c** Decoupled data [75] Copyright 2024, The American Association for the Advancement of Science

As shown in Fig. 15, Liu, Zhang et al. [75] designed an octopus-inspired, cage-like, three-dimensional (normal force, shear force, and strain) resistive, flexible sensor array. The researchers developed a mathematical decoupling algorithm based on static equilibrium analysis, with all decoupling formulas validated for accuracy through finite-element simulation and experimental calibration. For the normal force, a weighted summation of the relative resistance changes from 8 force sensors was used (Eq. 1 in Fig. 15). For shear force, it was first decomposed into x and y components based on the angular symmetry of the cage structure (Eq. 2 in Fig. 15), and then vector synthesis was applied to obtain the force magnitude and direction angle (Eq. 3 in Fig. 15). For strain, a linear relationship based on the simple piezoresistive effect was fitted, requiring only the calibration of a coefficient by applying a known tensile strain. The entire process achieved accurate, effective quantitative decoupling of three-dimensional signals solely through analytical formulas, without requiring machine learning. Prediction errors for shear force magnitude and angle were only 0.008 N and 1.5°, respectively, demonstrating the reliability and effectiveness of mathematical decoupling.

**Fig. 16:**
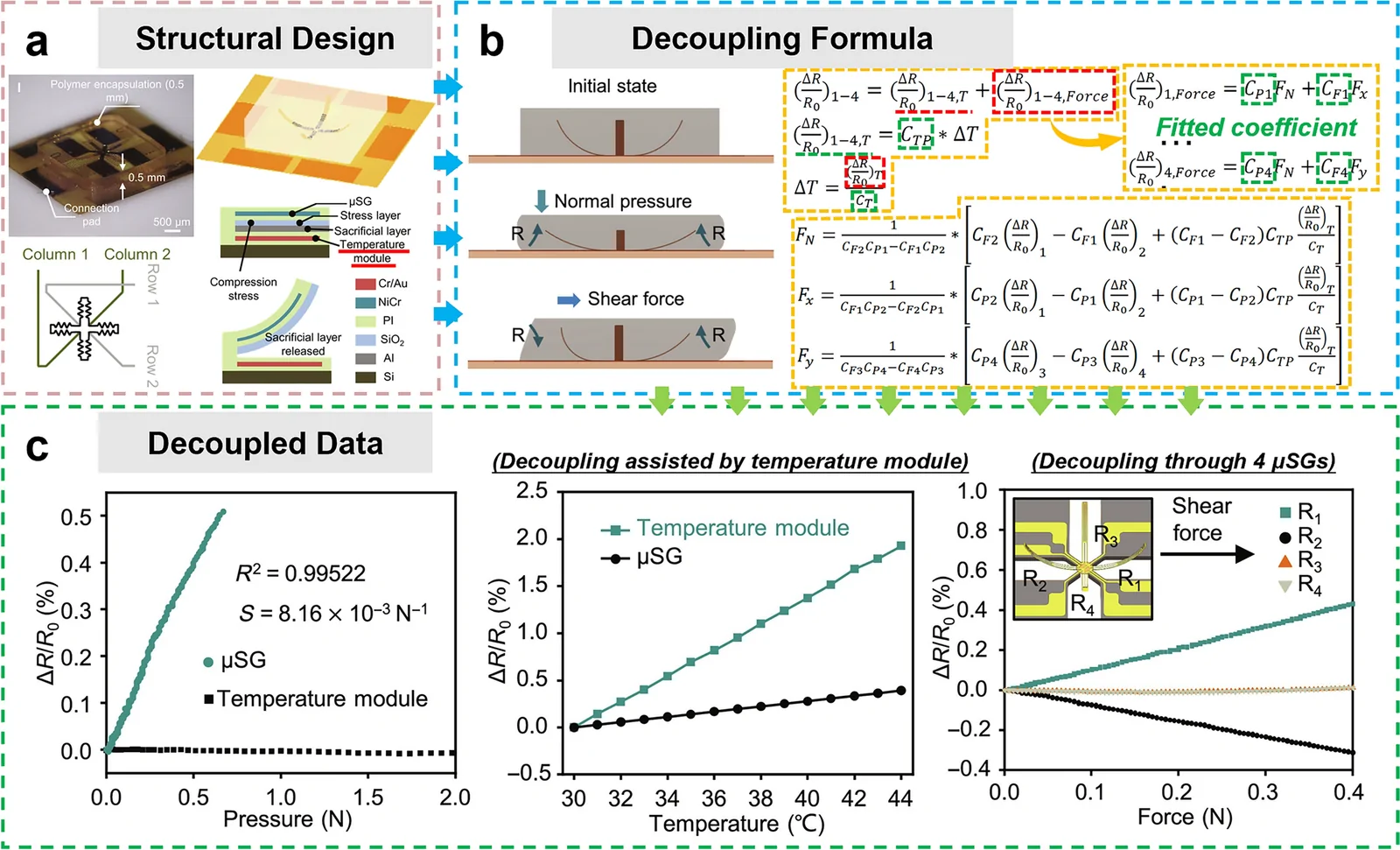
Schematic diagram of mathematical decoupling process (normal force, shear force, and temperature) of the μSGs flexible sensor array. **a** Structural design. **b** Decoupling formula. **c** Decoupled data [76] Copyright 2024, The American Association for the Advancement of Science

Using a similar approach, as shown in Fig. 16, Xu, Han et al. [76] designed a highly linear flexible sensor composed of orthogonally warped micro-strain gauges (*μ*SGs) and an independent Cr/Au temperature module. By deriving mathematical decoupling formulas from the principles of differentiated resistive responses and experimentally fitted linear relationships, the researchers achieved high-precision decoupling of three-dimensional/multifunctional quantities (normal force, shear force, and temperature), with decoupling deviations for each amount less than 8%.

*Decoupling algorithms using machine-learning models* have gained widespread adoption. Machine-learning algorithms are, in essence, powerful fitting tools adept at capturing and mapping relationships that are difficult to model explicitly. They can learn mathematical relationships from large volumes of data [83, 208] and are particularly effective for modeling highly complex nonlinear patterns [166]. The super-resolution algorithms mentioned previously [75, 210] also fall within this domain. Various algorithms have been successfully applied, including the multiple linear regression model [111], MLP [43, 75], convolutional neural network (CNN) [26], long short-term memory (LSTM) [118, 210], and hybrid architectures such as the CNN-LSTM-AM hybrid neural network [87].

**Fig. 17:**
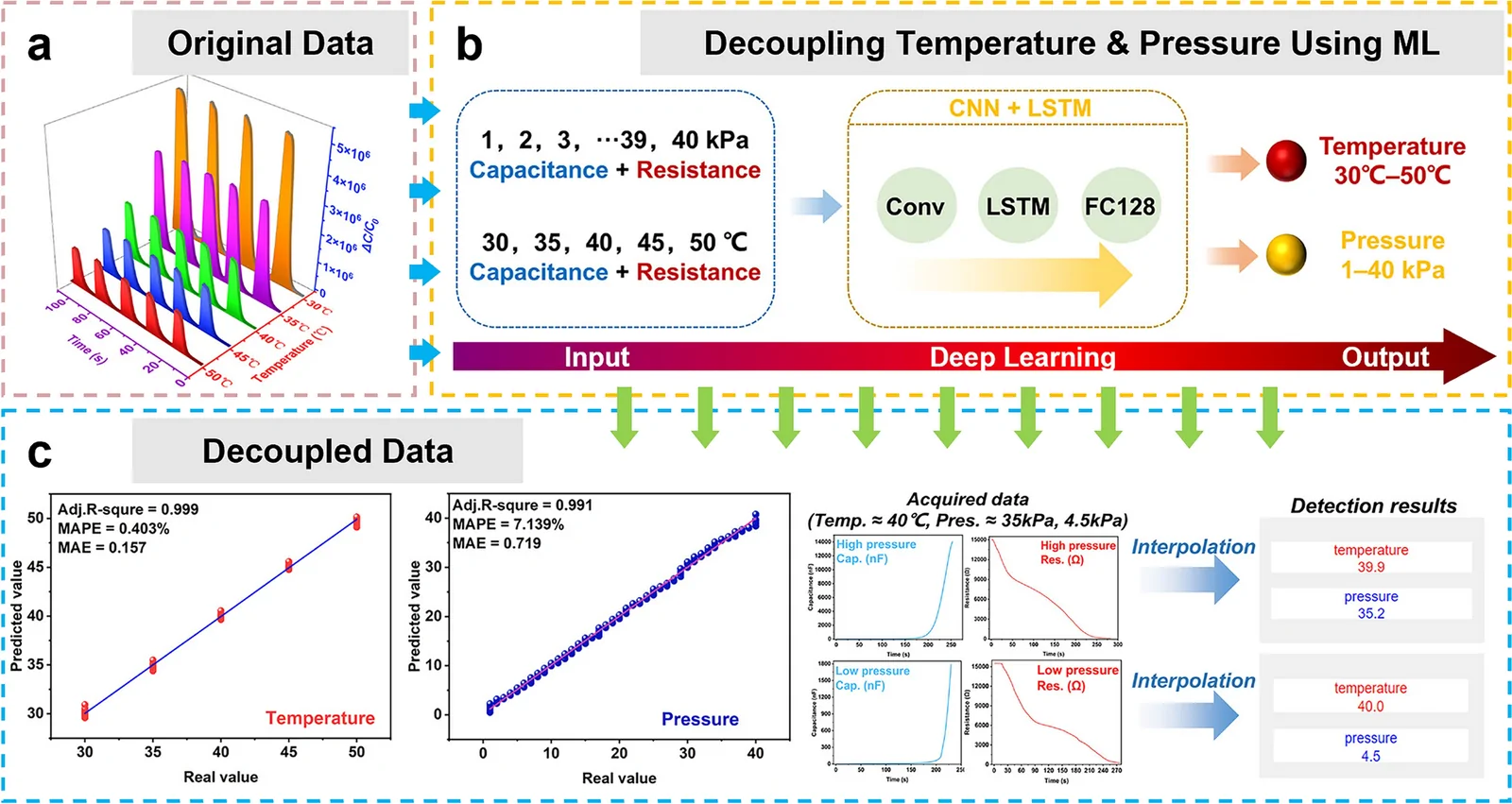
Decoupling of pressure and temperature with a CNN-LSTM hybrid network.** a** Original data. **b** Decoupling process. **c** Decoupled data [208] Copyright 2025, John Wiley and Sons

Pu, Zhang et al. [208] fabricated a bifunctional (pressure + temperature), bi-modal (capacitive + resistive) dual-dielectric-layer iontronic pressure sensor (DLIPS) with an Au/SCOG/PU foam/Au sandwich structure. The researchers trained a CNN-LSTM hybrid neural network (FC16 + 1Conv + 1LSTM + FC16 + FC128 + FC32 + 2) on a dataset of 2601 samples from a single sensor, covering a pressure (capacitive) range of 1 ~ 40 kPa and a temperature (resistive) range of 30 ~ 50 °C. This model achieved decoupling accuracies of 99.1% for pressure and 99.9% for temperature, respectively. In a subsequent silent speech recognition (SSR) application, the same DLIPS sensor was attached to the throats of four volunteers to capture signals during unvoiced articulation. An LSTM classification network (FC13 + LSTM16 + LSTM16 + FC16 + softmax) was then trained on a collected dataset of 600 samples (100 samples per word), achieving an overall recognition accuracy of 98.5% for 6 target words, as shown in Fig. 17.

### Interpretation Algorithms

In contrast, interpretation algorithms are designed to extract in-depth understanding from the information derived from decoupled data [269]. Such algorithms enable a more comprehensive and high-dimensional perception [88, 127], for example, the perception of gestures/postures/expression/emotion [140], texture [270], speech decoding [208], and physiological states [127]. This includes both in-depth mining of individual sensor data [40] and fusion of multi-sensor information [127].

*Interpretation algorithms using machine-learning models* play a key role in sensor systems. Machine-learning algorithms can not only decouple complex signals and recover specific physical quantities, but can also deconstruct and analyze the sensor data at a higher-dimension. This capability also transcends basic signal processing, because it can extract high-level information embedded in the data, recognize complex dynamic patterns, and, ultimately, it can realize intelligent decision-making and control, all of which represent a transition from mere sensing to actual cognition.

A technological framework should be used as required by the specific processing task and the data’s characteristics. For fundamental classification and regression tasks, traditional models are usually used, such as linear regression [71], partial least-squares regression [116], logistic regression [59, 108], Bayesian methods [108], support vector machines (SVM) [98, 112], random forest [108, 165], XGBoost [150], K-nearest neighbors (KNN) [214], and MLP [221]. For the handling of sensor data with spatial structure, CNNs [256, 259] and their derivatives, such as AlexNet [268] and ResNet [239, 252, 268], usually have exceptional feature-extraction capabilities for modeling complex spatiotemporal dependencies. For the processing of temporal dynamic information, sequence models with inherent memory can efficiently capture temporal dependencies, which include recurrent neural networks (RNNs) [127, 271], quasi-recurrent neural networks (QRNNs) [31], and LSTMs [118, 129]. In recent years, novel networks such as CNN-LSTM hybrid networks [208], LMN + ITM hybrid networks [127], encoder-CycleGAN cascaded hybrids [69], and transformer + CNN hybrid networks [268] have used the strengths of different networks and thus made breakthroughs in processing complex multichannel, multimodal data. The efficacy of these algorithms has been fully demonstrated in several representative cases.

**Fig. 18:**
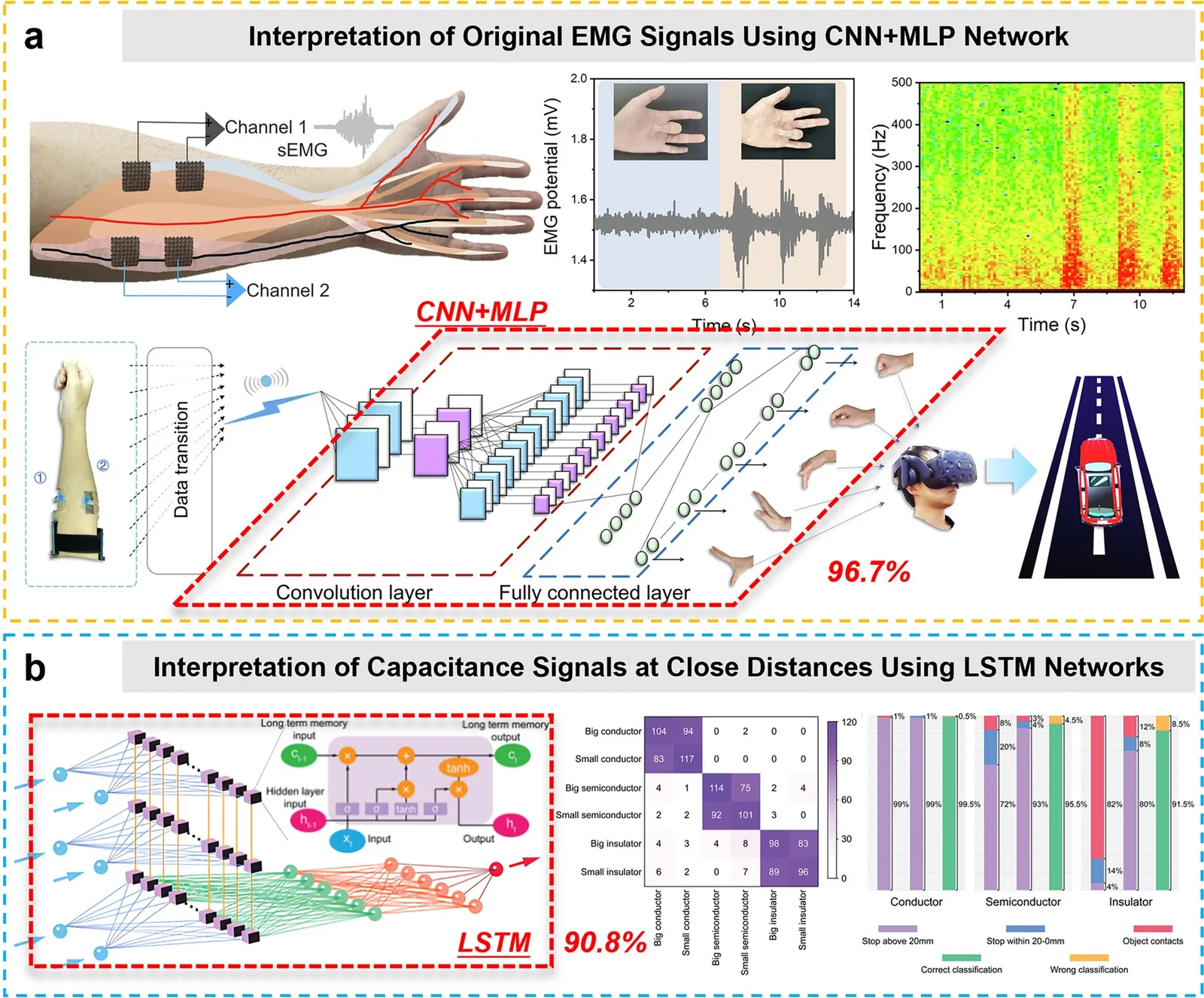
**a** Interpretation of EMG signals using CNN networks [79] Copyright 2025, Springer Nature. **b** Interpretation of capacitance signal for proximity distance via LSTM Network [89] Copyright 2023, John Wiley and Sons

As shown in Fig. 18a, Gao, Wang et al. [79] developed a flexible smart electronic tape (SET) with CNT-PDMS composite sensing material and serpentine-shaped structure. This device showed excellent biocompatibility, breathability, and adhesion, maintaining an SNR of 19.8 ± 0.48 dB after 5 days of service, which allows for precise acquisition of ECG and EMG signals.

The researchers further developed a CNN-based EMG gesture recognition algorithm with five convolutional layers and two fully connected layers, which processed 2D EMG signals of size 2 × 1000. It achieved an average recognition accuracy of 96.7% when trained on a dataset covering five gestures (right-hand swinging left/right, clenching a fist, extending the fingers, and natural relaxation) and with 130 data instances per gesture. Its recognition rate for thumb, ring finger, and little finger gestures reached 100%, thus enabling real-time control of a virtual vehicle’s steering, acceleration, and braking.

As shown in Fig. 18b, Ge, Zhang et al. [89] constructed a LSTM model and analyzed the object type (conductor, semiconductor, insulator, and their sizes, 6 types in total) using capacitive sequence data from the object’s approaching process as input. The classification accuracies were 99.5%, 95.5%, and 91.5% for the conductors, semiconductors, and insulators, respectively, with an overall accuracy of 90.8%. Based on the classification results, the researchers dynamically adjusted the detection threshold (5.7% for conductors, 4.2% for semiconductors, and 2.7% for insulators) and finally increased the robot’s safe recognition rate for insulators from 18% to 88%. This effectively solved the problem of traditional robots that cannot reliably detect insulating objects such as nylon and wood and are prone to cause collisions. It also significantly enhanced the safety and accuracy of human–robot collaboration.

In practical sensing systems, decoupling and interpretation algorithms are not mutually exclusive: interpretation algorithms are typically predicated on the efficient execution of decoupling algorithms. Although some studies do not explicitly mention a decoupling step and directly output interpreted results, their interpretation algorithms implicitly embed decoupling processes and functionalities.

**Fig. 19:**
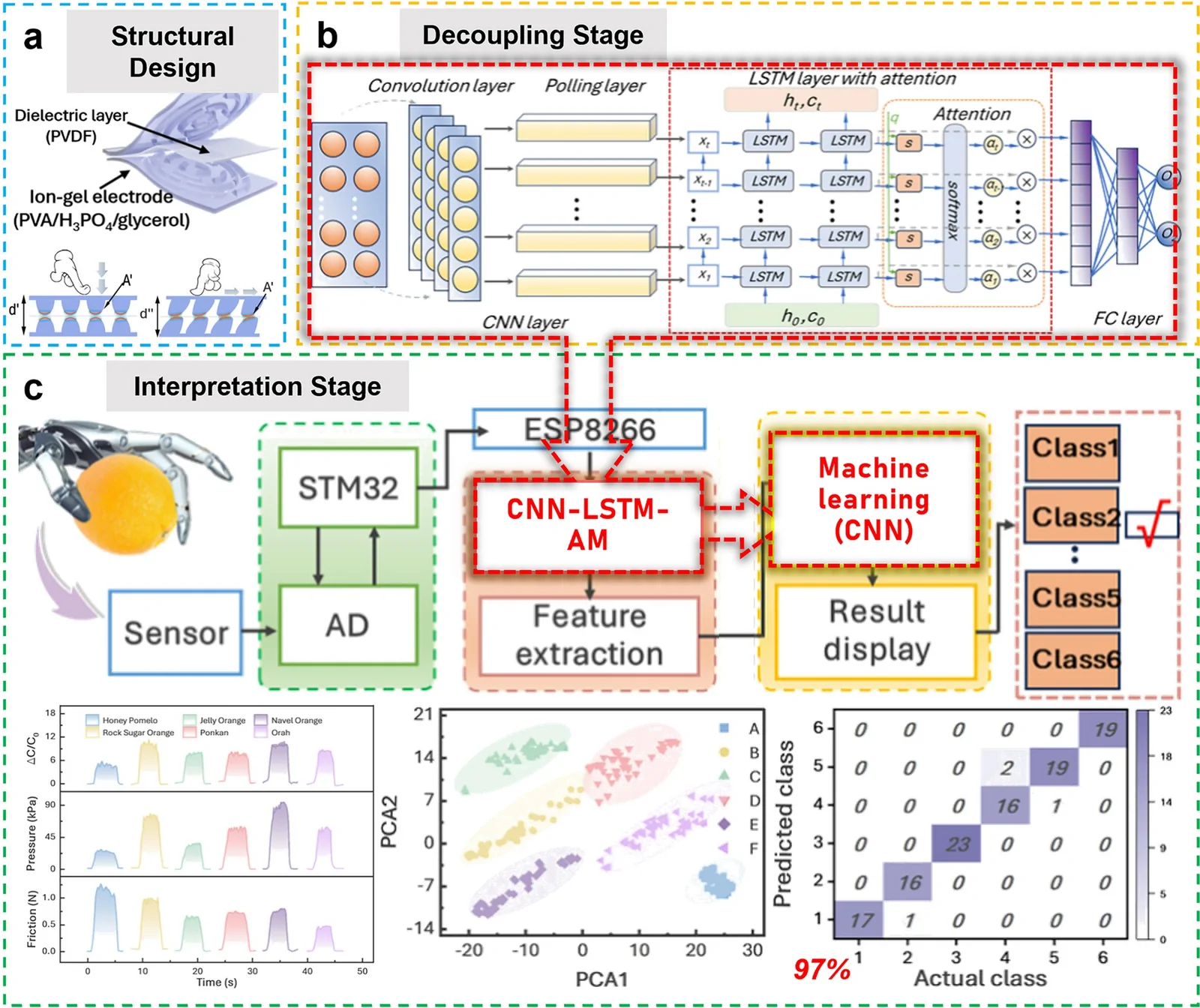
Decoupling of pressure and shear via a CNN-LSTM-AM hybrid network, and high-accuracy citrus variety differentiation using a CNN model with the decoupled data in the BFTS sensors. **a** Structural design. **b** Decoupling stage. **c** Interpretation stage [87] Copyright 2025, John Wiley and Sons

As shown in Fig. 19, Liu, Yao et al. [87] developed a bifunctional capacitive flexible sensor (BFTS) capable of simultaneously detecting pressure and friction, using a PVA/H_3_PO_4_/glycerol iono-gel as the electrode and PVDF as the dielectric layer, inspired by the mechanism of human fingerprints. A CNN-LSTM-AM hybrid network integrating CNN, LSTM, and a dual-head attention mechanism (AM) was designed to decouple the mixed signals of pressure and shear force. Specifically, the CNN component extracted local spatial features from the coupled signals (e.g., capacitance variations induced by pressure across sensor regions). At the same time, the LSTM modeled the temporal dynamics of the signals (e.g., continuous signal fluctuations during frictional sliding). The dual-head AM part adaptively assigned weights to key spatiotemporal features, thereby suppressing noise interference. This approach achieved high-accuracy signal decoupling, with coefficients of determination (R^2^) of 0.97 for pressure and 0.95 for shear. Leveraging the accurately decoupled pressure and shear data, higher-level feature extraction was performed. A subsequent CNN-based classification model was applied to distinguish 6 visually similar citrus varieties, achieving 97% classification accuracy.

### Edge-Deployment Optimization

Computational power, including number of chips, memory capacity, and operating frequency, affect the algorithm deployment strategy. Edge-computing devices (e.g., MCUs, SoCs) have limited computational power and undertake critical tasks including the control of flexible devices, operational amplifiers, power supplies, and signal transmission, which must be fully considered during the algorithm preparation stage.

For simple application scenarios, the required algorithms are generally lightweight (simple structure, few parameters, high operation speed, low memory occupancy, and low-power consumption). In such cases, algorithms can be deployed on these edge-computing devices to realize timely local processing of front-end signals, yielding sensing systems with extremely low latency and fast response (in-sensor computing and near-sensor computing described earlier represent the ideal form for this scenario).

For complex scenarios, sophisticated algorithms are unavoidable, requiring the transmission of raw data (or data after simple local preprocessing) to a host with superior computational power for algorithm execution. In this case, edge-computing devices focus on control tasks, but the system incurs higher latency and power consumption. Therefore, algorithm design must strike a trade-off among accuracy, computational power, and power consumption, and model compression techniques should be considered to adapt to edge hardware constraints.

### Optimization and Challenges

While machine-learning methods have demonstrated excellent applicability in solving nonlinear, non‑analytical problems, they also present limitations, such as the need for extensive training datasets, high training costs, and susceptibility to noise. Several new algorithms and optimization strategies have been proposed to overcome these limitations, including data smoothing [26], multi-network ensemble learning [12], data augmentation [239], transfer learning [93, 127], semi-supervised learning [92, 117], and hybrid neural networks [12, 56]. Rather than simply incrementally improving accuracy, these technologies address the fundamental bottlenecks of real-world deployment. By alleviating the burden of massive data collection and bolstering robustness against dynamic environmental clutter, they drive sensor systems toward truly adaptive and highly reliable edge-intelligent platforms. Some representative works on sensor algorithms are summarized in Table 7.

**Table 7:** Comparison of representative algorithms for sensors

Function	Algorithm	Sensors / signal dimensions	Dataset	Scenario	Accuracy	Ref
Decoupling algorithms	MLP (8 + FC20 + FC10 + 6)	Single sensor with 8-channel signals (2 electrodes per channel)	418	Decoupling of single-point six axis force/torque	Fx: 99.3% Fy: 99.5% Fz: 99.9% Mx: 99.9% My: 99.9% Mz: 99.8%	[43]
	LSTM (LSTM + 2BiLSTMs + 1LSTM + 1FC)	3-channel sensor signals (one each for temperature/pressure/strain)	Not mentioned	Decoupling of stretch and temperature	Stretch: 85%	[118]
	LSTM (2BiLSTMs + 1LSTM + 1FC)	3-channel sensor signals (one each for temperature/pressure/strain)	Not mentioned	Decoupling of pressure and temperature	Press: 93%	[118]
	CNN-LSTM hybrid neural network (16 + 1Conv + 1LSTM + FC16 + FC128 + FC32 + 2)	(2,16) shape data with single sensor, dual-mode signal and sequential sampling	2601	Decoupling of pressure and temperature	Press: 99.9% Temperature: 99.1%	[208]
Interpretation algorithms	SVM	22-channel pressure sensor signals	1275 (8 postures in total)	Posture recognition	100%	[165]
	Random forest (100 trees)	22-channel pressure sensor signals	1275 (8 postures in total)	Posture recognition	100%	[165]
	CNN (22 + 2Convs + 1FC + softmax)	22-channel pressure sensor signals	1275 (8 postures in total)	Posture recognition	99.55%	[165]
	TCNN-LSTM hybrid neural network	1-channel sensor signals	~ 27,000 (6 gestures of 40 volunteers)	Hand movement recognition	94.72%	[100]
	Transfer learning LMN + ITM hybrid neural network	8-channel EMG sensor signals	300 (14 gestures in common) based on pre-trained model	Gesture recognition by EMG	98.03%	[127]

Performance data adopted in this table are as reported in the original literature

## Coupling

The core of high-performance sensor systems lies in the disciplined art of harnessing faint signals from the physical world and converting them into reliable digital information. Therefore, a sensor system must be designed considering the trade-offs across the entire chain, from the operating principle, material selection, structural design, the hardware implementation, until the signal processing algorithms.

### Integration of Materials, Structure, Hardware, and Algorithms in Sensor Systems

**Fig. 20:**
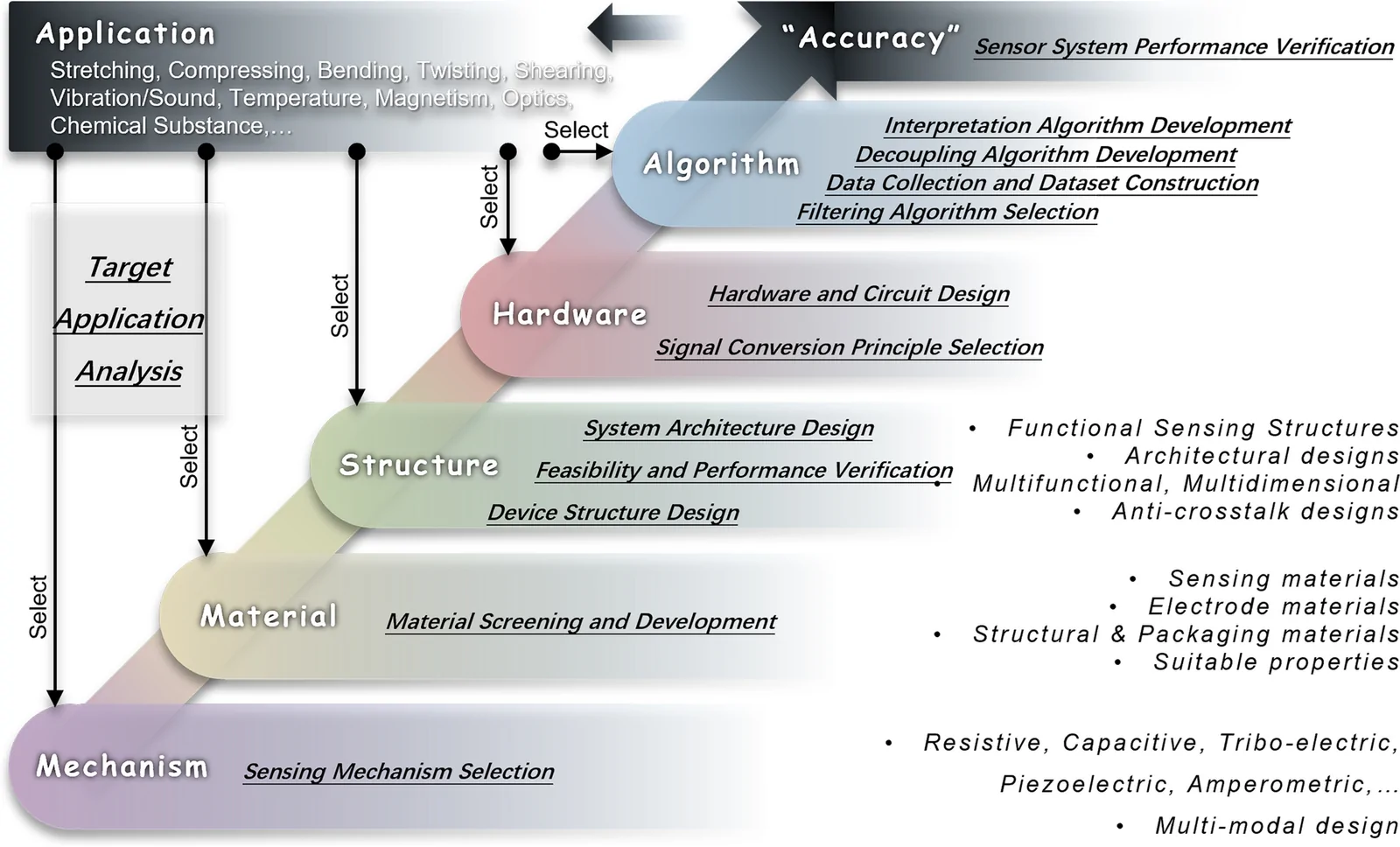
Holistic approach to sensor systems throughout the entire process from basic mechanisms, materials, structures, hardware to algorithms

A high-performance sensor system has to be constructed through the collaborative innovation of key links in order to achieve their synergistic improvement for better critical performance metrics such as sensitivity, range, linearity, response time, and stability toward specific external stimuli. These links include material selection, structural design, hardware integration, and algorithm optimization. It is also necessary to conduct targeted screening and integration of the core links based on the differentiated requirements (e.g., environmental stability, biocompatibility, and cost control). Any compromise along this chain can influence the final performance ceiling of the overall system, as shown in Fig. 20.

Single-level innovation—whether in the synthesis of new materials, the fabrication of microstructures, the design of low-power hardware, or the development of high-performance algorithms—undoubtedly constitutes the core driving force advancing the field of flexible sensors. Such innovations continuously push the performance limits of individual dimensions and provide higher-quality “components” for system integration. However, when these isolated “pearls” are integrated into a complete sensing system, a deep-seated dilemma emerges: state-of-the-art materials may suffer performance degradation due to incompatibility with subsequent structural processing; elaborately designed structures may be overshadowed by noise from readout circuits; and powerful algorithms, without understanding the front-end physical characteristics, can only provide limited compensation within the constraints of a “black box”. This is exactly the “silo effect” facing the current field: each subfield progresses rapidly along its own track, yet the system-level performance ceiling is often determined by the most lagging “bottleneck”.

The value of this co-design paradigm lies precisely in breaking this fragmented, isolated development. It pursues not the extreme parameters of individual devices, but the globally optimal solution of the system under real-world application scenarios. This collaboration is not a simple linear superposition, but a deep synergy: material selection must anticipate the feasibility of structural fabrication; structural deformation must be converted into electrical signals detectable by hardware; the power consumption and noise of hardware must reserve compensation space for algorithms; and algorithm design must inversely capture the full physical transfer function from materials to hardware. Only through such deep integration across disciplinary boundaries can the systematic performance dividend be unleashed, which is rarely attainable through any single-point optimization.

In classical component-based design and sequential optimization paradigms, there is a pervasive reliance on the paradigm of “additive enhancement”—a linear integration approach which presumes that simply stacking the peak performances across the material, structural, hardware, and algorithmic tiers will inherently yield an optimal overall system (i.e., the expectation of a quantitative accumulation where “1 + 1 + 1 + 1 > 4”). However, as cutting-edge flexible electronic systems increasingly exhibit a highly coupled developmental trajectory in practical applications, the mathematical premise of this purely additive model becomes physically incompatible with systemic realities.

Accordingly, we aim to move beyond simple linear additive thinking, as the core thesis of our work is inherently nonlinear. The essence of the four hierarchies co-design paradigm lies not in the mechanical superposition of extreme individual parameters, but in achieving a holistic systemic leap through “mutual constraint, adaptive compromise, and trade-off resolution (Synergy)”. True synergy mandates the deliberate relinquishment of extreme peak metrics in specific components (e.g., sacrificing ultimate absolute sensitivity in favor of superior linearity) to secure globally optimal information flow. In this context, the “gain” manifests as the emergence of expanded application boundaries and systemic robustness in complex, real-world scenarios, rather than a cumulative mathematical high score. As explicitly delineated in Table 8, the underlying definition of “performance gain” undergoes a profound paradigm shift across these contrasting research paradigms.

**Table 8:** Comparison of optimization paradigms in flexible sensor systems

Optimization hierarchy	Performance gain	Type of evidence	Typical evaluation metrics	Example scenarios	Limitations
Single-hierarchy optimization (e.g., material or algorithm only)	Maximizing extreme performance of individual components	Isolated characterization of a single property	Specific physical parameters (e.g., conductivity, micro-structure, modulus)	Fundamental mechanism discovery, new material synthesis	“Silo effect”: Extreme component performance may cause mismatches or failures in system integration
Partial co-design (e.g., material + structure)	Coupling adjacent mechanisms to improve specific performance and decouple local physical contradictions	Device-level cross-correlation experiments	Device-level functional metrics (e.g., sensitivity, linearity, range)	High-performance devices development	Often lacks optimization for downstream data extraction, remaining vulnerable to hardware noise or complex data interpretation
Full-chain co-design (M + S + H + A)	Holistic synergy and system-level trade-off resolution. Components constrain and compensate for each other	System-level validation under realistic/complex operating conditions	System-level task efficiency & robustness (e.g., Recognition accuracy, Environmental adaptability)	System-level applications such as intelligent human–machine interfaces	High multi-disciplinary barrier; lacks unified quantitative evaluation frameworks to benchmark synergistic gains

Clarifying this systemic philosophy by no means negates single-tier innovations; conversely, the two are profoundly complementary. Single-tier breakthroughs furnish the ultimate high-performance “building blocks”, whereas co-design serves as the “architect”. It dictates how these discrete modules must mutually adapt and make concessions during integration, thereby dismantling the silo effect of single-point optimization, broadening the holistic application boundaries of the system, and satisfying increasingly stringent real-world imperatives.

An ideal sensor system design should follow the full-chain closed-loop logic of “application-defined requirements – requirement-driven design – design hierarchical optimization”.

Specifically, it should start with a comprehensive and thorough analysis of the application scenarios to accurately identify the essential characteristics of the target sensing objects (e.g., the type and dynamic characteristics of related quantities), and clarify the environmental conditions that the sensor will face under actual working conditions (e.g., temperature, humidity, electromagnetic interference, mechanical loading), so as to determine specific and quantifiable performance indicators [272]. These include: basic performance metrics (LOD, range, response, sensitivity, resolution, bandwidth, repeatability; see Table 2), environmental adaptability (e.g., corrosion resistance, mechanical robustness), and application-specific requirements (e.g., biocompatibility, flexibility/stretchability, self-healing/reconfigurable/recyclable properties, optical transparency). Based on this requirement list, the most suitable sensing mechanism shall be determined; a multimodal design can be adopted if necessary (see Table 1 and Sect. 2).

The subsequent “materials–structure–hardware–algorithm” co-design is the core step that determines the fundamental performance of the sensor. This is not a strictly sequential, one-way pipeline, but rather a dynamic, iterative process characterized by continuous bidirectional feedback among the following steps:

- 1. Screen or develop sensing materials, electrode materials, structural and packaging materials based on the unique material requirements of the determined mechanism, the material preferences specified in the requirement list, material processability, mechanical compatibility between materials, and interface connections (see Table 3 and Sect. 3);
- 2. Conduct customized device structure design (e.g., micropillar arrays, porous structures, island-bridge structures) according to the specific preferences of the requirement list for performance parameters (e.g., high sensitivity, wide linear range, large range) to directionally optimize device performance, with attention paid to crosstalk suppression (see Table 4 and Sects. 4.1 and 4.4);
- 3. Verify manufacturability and sensing performance indicators of the device (see Table 2);
- 4. Determine the system architecture of the sensor according to the requirements for the target information dimension (multidimensional/multifunctional/single-point/multi-point/array detection), including the type, quantity, layout of devices, and overall shape (see Sects.  4.2–4.4);
- 5. Select the signal conversion principle according to the determined sensing mechanism and the preferences in the requirement matrix (Sect. 5.2);
- 6. Carry out hardware and circuit design based on the architectural design, signal conversion principle, and device structure, including the number and layout of signal-extraction electrodes, front-end design for signal readout paths (e.g., filter circuits, conversion circuits, bridge circuits), hardware scheme design (chip selection, power supply scheme, computing power and power consumption budgeting, communication mode, communication protocol and bandwidth), concrete implementation of time-division gating strategies, electrical design for crosstalk resistance, as well as necessary filtering and anti-electromagnetic interference designs;
- 7. Select or develop corresponding filtering algorithms (e.g., Kalman filtering, wavelet transform) for noise and interference in raw signals;
- 8. Collect data and construct high-quality datasets according to the target application, device structure, architectural design, and received signal dimensions;
- 9. Design decoupling algorithms for the target application, device structure, architectural design and device performance;
- 10. Develop interpretation algorithms combined with target applications (e.g., gesture recognition, health assessment, fault diagnosis);
- 11. Perform system-level performance verification.

This workflow also remains valuable for studies focusing on innovations in a single aspect (new materials, new structures, new hardware, or new algorithms). It provides a lens of “global vision”: it inspires materials scientists to proactively consider the compatibility of new materials with subsequent structures and circuits during development; makes structural designers aware of the potential impacts of microtopography on electrical performance; and encourages algorithm developers to actively draw on their understanding of the physical properties of devices, rather than simply regarding them as “black boxes”. This paradigm enables every “point innovation” to clearly anchor its position and interface specifications in the overall system at the outset, so that local breakthroughs can be translated into system-level performance gains more quickly and accurately.

Compared with the traditional paradigm, the core breakthrough of this materials–structure–hardware–algorithm co-design lies in the shift from “local breakthrough” to “system-wide optimality”. Its potential impact on sensor performance is reflected in the release of system-level performance dividends that are highly challenging to achieve through single-aspect optimization. Through deep coupling and two-way feedback between materials, structure, hardware, and algorithm, this paradigm can break through the traditional performance “ceiling” and approach the theoretical limit of the system. It requires researchers not only to possess a profound understanding of all four hierarchies but also to build bridges at the intersection of the scientific principles governing each level. This entails high learning costs, technical barriers, and financial investment.

Encouragingly, this trend has begun to emerge at the scope of partial co-design. Several studies focusing on bi-level integration, specifically the synergistic coupling of materials and structure (as summarized in Table 4), have pioneered the feasibility of cross-boundary collaboration. Instead of relying solely on intrinsic material synthesis, these works deliberately match specific material properties with engineered structural topologies (e.g., porous, mesh, or micro-protrusions) to cooperatively amplify device-level functional metrics, such as sensitivity, linear range, and response time. This empirical evidence demonstrates that opening the “interfaces” between just two hierarchies can effectively overcome the performance bottlenecks inherent to single-level optimization.

What truly reveals the revolutionary potential of this paradigm is the pioneering work that dares to challenge full-chain co-design. In the following two sections, we specifically summarize two highly representative studies that demonstrate the systematic wisdom of hierarchical coupling and synergy. They reveal a compelling trend: in contrast to performance improvements typically achieved by innovations within a single hierarchy, full-chain co-design works demonstrate powerful adaptability beyond what is typically possible in many application scenarios—a combination where the whole fundamentally exceeds the sum of its parts. Although direct numerical comparisons between different studies remain challenging due to the lack of standardized benchmarks, the qualitative leap in system capability provides strong empirical evidence for the co-design paradigm.

While the proposed framework demonstrates robust theoretical comprehensiveness, it must be explicitly acknowledged that, at the current stage, the validation of this “material-structure-hardware-algorithm” paradigm relies primarily on qualitative logical deduction and the semi-quantitative analysis of representative cases. Precisely because of the intrinsically nonlinear and highly coupled nature of this system (as discussed previously), traditional scalar quantification is rendered inadequate. Developing universally applicable unified quantitative evaluation frameworks—ones capable of dynamically quantifying “synergistic trade-offs” rather than mere “additive gains” across heterogeneous tiers—remains an exciting and formidable frontier challenge for the entire field. Therefore, the current core utility of this co-design framework manifests not in a unified cumulative numerical score, but in mapping optimal systemic compromises and structural boundary expansions (as detailed in Table 8). A further perspective on the urgent need for standardized nonlinear evaluation metrics is provided in Sect. 8.

### Hierarchical Optimization for High-Performance Sensor Systems

**Fig. 21:**
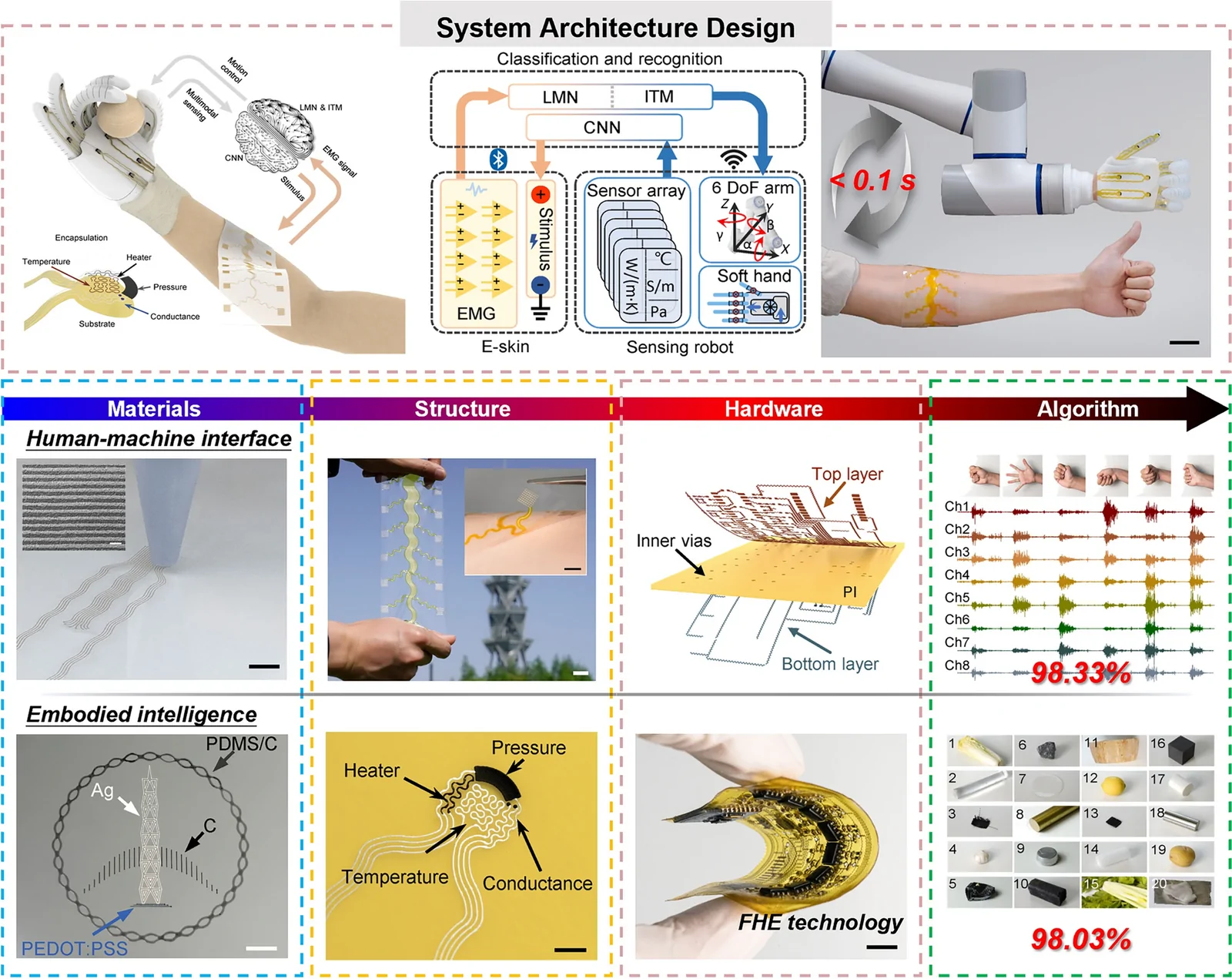
Typical examples of hierarchical optimization coupling design: Architecture design of the sensing system. Materials and process selection. Structural designs. Hardware implementation. Algorithmic processing [127] Copyright 2025, The American association for the advancement of science

As illustrated in Fig. 21, Wang, Yu et al. [127] developed a fully printed human–machine sensing system suitable for scalable manufacturing, comprising two independent subsystems: human–machine interface terminal and embodied intelligence terminal.

The human–machine interface terminal consisted of an 8-channel surface electromyography (sEMG) sensor with inkjet-printed silver-paste electrodes featuring a mesh-like, serpentine structure in a split configuration. It operated on the principle of differential potential measurement to capture electrical signals generated by muscle contraction. This was coupled with precision amplification and filtering circuits and an STM32F103C8T6 MCU chip to enable real-time detection of EMG signals. A hybrid LMN + ITM neural network incorporating transfer learning, where LMN adjusted signal weights to accommodate individual differences, and ITM captured temporal features, enabled efficient operation in data-scarce and low-edge-computing scenarios. The system achieved 98.33% recognition accuracy across 14 distinct gestures, with a latency of only 0.1 s.

The embodied intelligence terminal consisted of a multimodal sensor system integrating four sensing mechanisms: pressure (capacitive mode, with a printed layered structure of C electrode/CB + PDMS foam/PVDF + IL/C electrode), temperature (resistive mode, using printed carbon ink), thermal conductivity (a carbon heater + a temperature sensor), and electrical conductivity (using an operational amplifier conversion method, with PEDOT:PSS hydrogel electrodes). These components were integrated onto a PI substrate using a flex-on-chip flexible hybrid electronics design and an ESP32 MCU chip, resulting in a highly compact flexible sensing system. A compact CNN neural network processed the four-modal data, achieving 98.03% recognition accuracy for 20 different objects.

The two subsystems communicated via Bluetooth in a closed loop (enabling bidirectional interaction between the ESP32 and STM32) to achieve coordinated operation, collectively forming a high-performance prosthetic system intended for individuals with limb impairments. In practical tests with amputees, the system achieved 89.82% recognition accuracy after only 5 user gesture repetitions. This work presented a low-cost, highly adaptable human–machine interaction solution for medical prosthetics and collaborative robotics.

More importantly, it exemplifies the advantages of full-chain, hierarchical co-design spanning materials, fabrication, structures, hardware, and algorithms over traditional single-level optimization strategies. By synergistically integrating inkjet printing, multimodal sensing, flexible hybrid electronics, and transfer learning, the proposed system achieves markedly improved performance in recognition accuracy, response latency, integration density, and real-world applicability. These results clearly highlight that cross-level co-design, rather than isolated design at any single hierarchy, is essential for unlocking the full potential of intelligent sensing systems.

### Coupled Design Strategies for Flexible and Adaptive Sensing Solutions

**Fig. 22:**
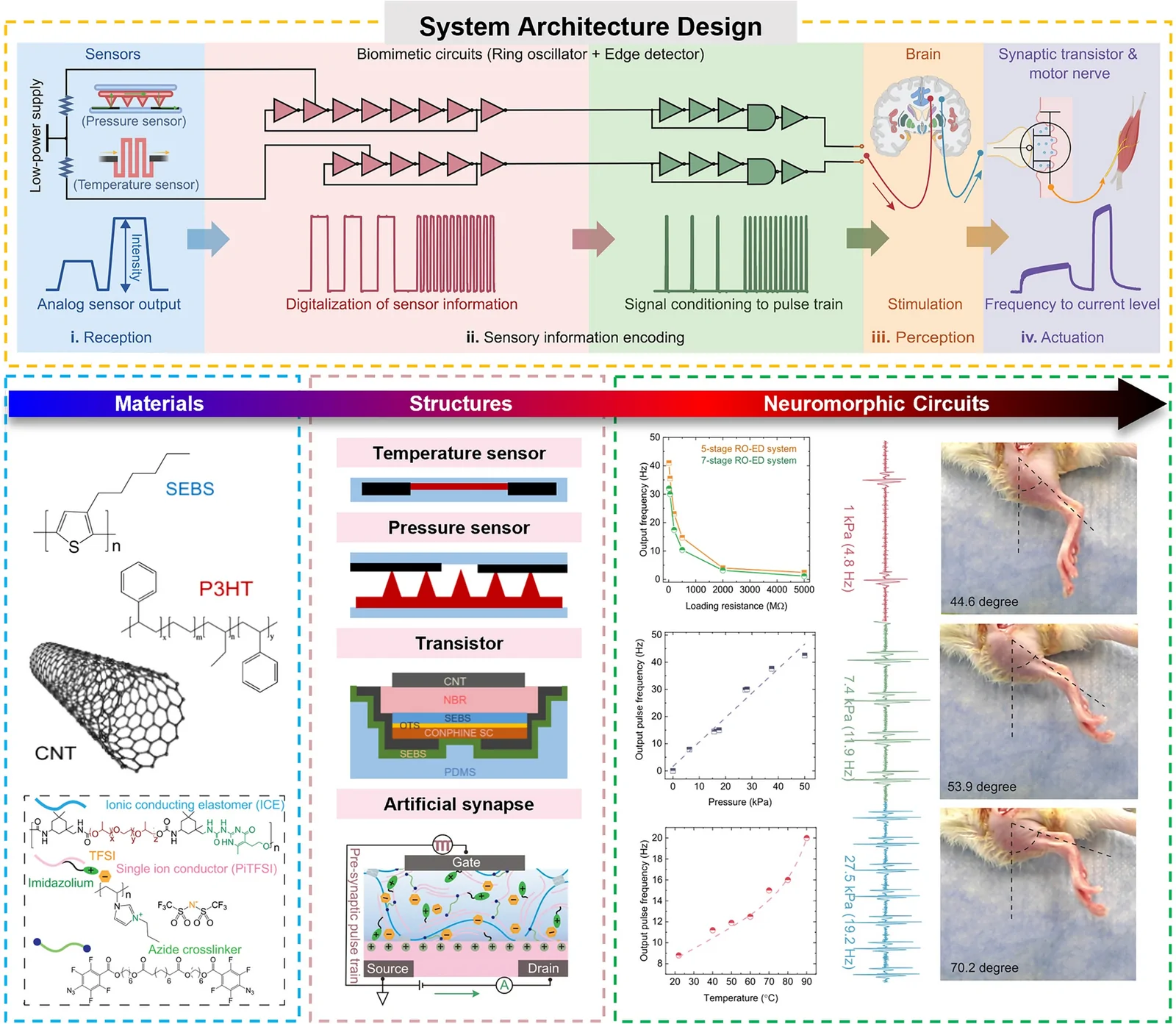
Typical examples of hierarchical optimization coupling design: Architecture design of the flexible sensing system. Material selection. Structural design. Signal processing logic and neuromorphic circuits design [132] Copyright 2023, The American association for the advancement of science

Wang, Bao et al. [132] designed a single-chip, low-voltage, implantable, flexible sensing system, as shown in Fig. 22. Through the co-design of materials, devices, and hardware, they realized a bionic sensory-motor circuit that interfaced directly with biological systems. The system integrated three primary modules: multimodal sensors that acquired signals, a neuromorphic signal conditioning circuit (comprising a ring oscillator and an edge detector, 54 thin-film transistors in total) that collected sensing signals and converted them into amplitude-decoupled frequency-modulated pulses (1–50 Hz), matching the characteristics of biological action potentials, and PEDOT:PSS biocompatible electrodes. Pulses were transmitted through PEDOT:PSS electrodes into the somatosensory cortex of rats to elicit neural responses. Solid-state artificial synapses then converted the response signals into variable-amplitude output currents, ultimately enabling stimulation and actuation of biological muscle tissue.

The sensors consisted of a resistive pressure sensor with a pyramidal microstructure composed of CNTs, and a temperature sensor, capable of detecting pressure in the range of 0 ~ 50 kPa and temperature in the range of 22 ~ 90 °C. The neuromorphic signal conditioning circuit comprised 54 low-voltage stretchable organic transistors featuring a triple-layer dielectric design. The NBR/SEBS/OTS triple-layer gate capacitor configuration enabled operation at a safe voltage of 3 V, achieving a peak mobility of 2.01 cm^2^ V^−1^ s^−1^ and a static power consumption of only 0.25 pW. The solid-state artificial synapse, fabricated using an ion-conducting elastomer (ICE) and a polyelectrolyte (PiTFSI), avoided stability issues associated with liquid components.

All components were fabricated from flexible, stretchable materials with excellent biocompatibility. Through meticulous materials and structural design, high device performance, density, and stability were achieved, with an array density of 330 devices cm^−2^. The system maintained functional stability even under 100% strain, and sensors attached to the skin surface for 30 h induced no irritant reactions, confirming good biocompatibility. After being immersed in water or physiological solution at 37 °C for 8 h, the output current of the artificial synapse changed by less than 10%, much better than traditional liquid-based synapses, which showed 98.2% current loss.

The functionality was validated in rat experiments, where the varied pressure correspondingly increased the leg twitch angles from ≈ 44.6° to ≈ 70.2°, successfully mimicking the positive correlation between the stimulus intensity and the observed motion amplitude in natural skin.

This work illustrates the potential of a full-stage hierarchical co-design paradigm over traditional single-level optimization. The proposed system covers materials, structures, hardware, and neuromorphic signal processing. It seamlessly bridges artificial sensors with biological neural networks by synergistically integrating biocompatible elastomers (materials), triple-layer dielectric and pyramidal microstructures (structures), low-voltage stretchable transistor arrays (hardware), and biomimetic frequency-modulated pulse logic (algorithms/system logic). This cross-level co-design fundamentally resolves the inherent trade-offs between mechanical compliance, electrical performance, and physiological stability. In addition, it realized several milestones that is highly challenging for isolated component-level improvements, which are ultra-low-power consumption, high array density, and robust operation under extreme strain and wet environments. Ultimately, this work suggests that an integrated, hierarchical design strategy is essential for realizing next-generation, high-performance bio-machine interfaces.

## Prospects and Outlooks

Flexible sensors have demonstrated enormous potential across multiple fields [273], significantly reshaping the practical manifestations of two core concepts, human–computer interaction (e.g., gesture recognition [186], virtual reality [256]) and embodied intelligence [270, 274], and profoundly revolutionized key application domains such as healthcare [275], smart home [276], industry [11], and agriculture [277].

**Fig. 23:**
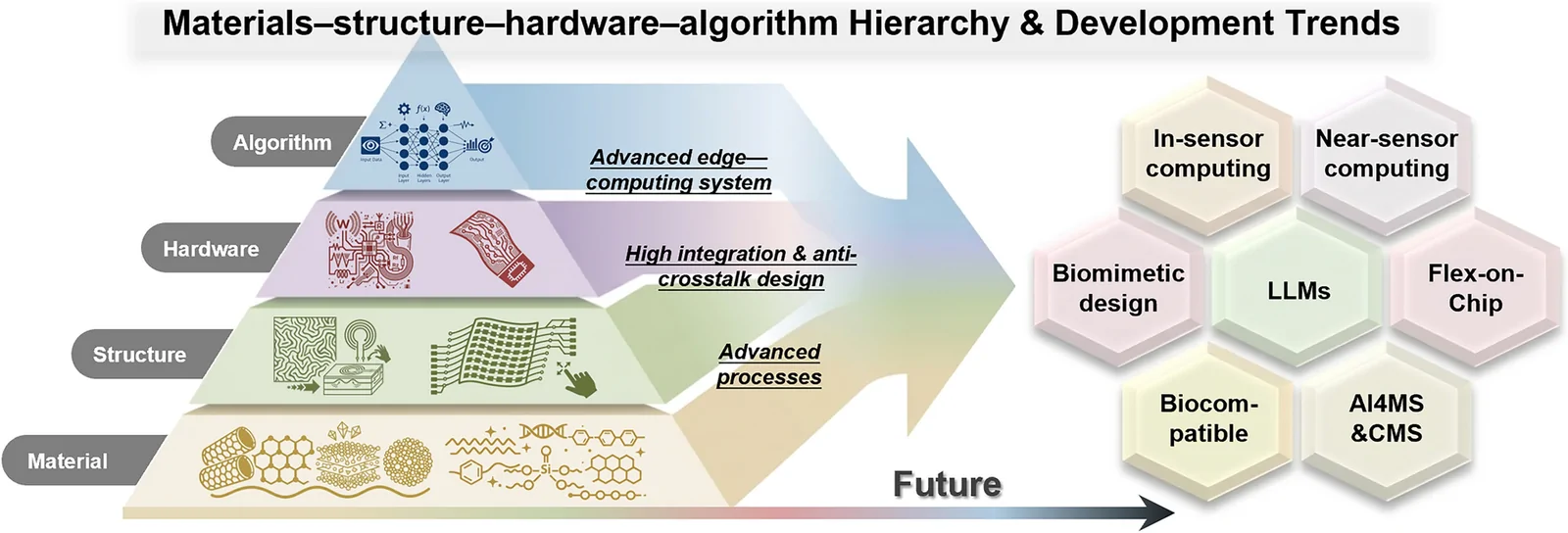
Materials–structure–hardware–algorithm co-design paradigm: Mechanism → Material → Structure → Hardware → Algorithm, and related development trends

Currently, scientific and technological breakthroughs continue to emerge in this field, which is now in a critical stage of development [37]. Among these advances, the hierarchical co-design paradigm spanning the materials–structure–hardware–algorithm hierarchy overcomes the inherent limitations of traditional fragmented design methods through its system-level optimization. This paradigm has thus become an indispensable pathway to drive further breakthroughs in the field [113], as illustrated in Fig. 23. In contrast to the conventional paradigm that only pursues innovation at an isolated stage, this approach can unlock system-level performance dividends beyond the reach of single-component optimization. It demonstrates strong developmental potential and is expected to become the core methodology for flexible electronics in the future.

It should be emphasized that this is inherently accompanied by relatively high costs and technical barriers. The core objective of this paper is to conceptualize this emerging approach, systematically review the technical frameworks of the related fields, and inspire researchers to adopt a co-design mindset, thereby advancing the overall development of the field. We fully recognize that innovations in individual components—including new materials, novel structures, emerging hardware principles, and advanced algorithms—will remain crucial driving forces for the future progress of this discipline.

For studies focusing on single-component innovation, the co-design paradigm proposed here provides a “holistic perspective” for researchers in the highly interdisciplinary field of flexible sensors, allowing every point innovation to be grounded in a system-level view from the outset: It delivers demand-driven guidance for new material development, encouraging materials scientists not to simply pursue extreme values of individual parameters, but to conduct targeted design according to system-level requirements such as processability and interfacial compatibility; It identifies application targets for structural exploration, helping researchers in structural design clarify their roles within the overall system; It establishes interface specifications for new hardware development, ensuring that circuit designs are naturally compatible with front-end sensitive materials and back-end algorithms; It provides physical priors for new algorithm design, integrating an understanding of device architecture and material properties into algorithm development.

With growing demand for relevant applications, thoroughly understanding and systematically integrating the principles of co-design is crucial for the development and deployment of next-generation high-performance, flexible sensors.

Looking forward, we outline the following key development trends:

*Diversification and Integration of Sensing Mechanisms*. Traditional sensing mechanisms (e.g., resistive, capacitive, and piezoelectric) have become well‑established, while emerging mechanisms including force‑thermal field conversion [43], impedance spectroscopy [105], and magnetic sensing [107, 108] are continuously expanding the boundaries of perception. More importantly, the inherent limitations of single‑mechanism sensing have driven the rise of multimodal fusion design. By integrating orthogonal information, such designs can not only enhance the detection dimensionality but also enable cross‑validation and interference suppression via information redundancy. Multimodal sensing is thus shifting from an “optional solution” to a “necessary strategy” for complex application scenarios.

*Emergence and Innovative Applications of New Materials.* In addition to referring to inventions of entirely new materials [185, 244], this also includes the widespread adoption of specialized materials such as 2D materials [273], self-healing materials [232, 278], bio-based/degradable materials [149], biocompatible materials [279], metamaterials [280], edible materials [281] and textiles [282], as well as the enhancement of properties in existing materials through innovative methods (e.g., solvent welding [192], mono-polymer-matrix system design [31], tunable-modulus/stiffness design [33], and particle engulfment [193]) and their deployment in novel application scenarios. Concurrently, the growing adoption of artificial intelligence for materials science (AI4MS) and computational materials science (CMS) is significantly accelerating the discovery of new materials and the optimization of manufacturing processes [117], even enabling scenario-specific material customization.

*Customizable Performance and Continuous Expansion of Information Dimensionality.* Driven by in-depth research into sensor structures, the field of flexible sensing is undergoing a profound shift toward “on-demand customization”. Significant advancements in structural design methodologies—including biomimetics [230], topology optimization [283, 284], and high-precision finite-element simulations [259, 285, 286]—have empowered researchers to precisely modulate device structural features, thereby enabling the programmable customization of individual sensor performance. At the system level, the information dimensionality of flexible sensing systems is continuously expanding to accommodate increasingly complex and diverse application scenarios. This leap in information acquisition extends beyond merely increasing the array density of homogeneous sensors; instead, it is evolving toward the deep integration and synergistic fusion of heterogeneous, multifunctional, multidimensional, and multimodal sensors [238, 287]. Drawing inspiration from the human somatosensory system—where densely distributed mechanoreceptors in the fingertips provide millimeter-level spatial resolution, while high-frequency vibration receptors in the palm are specialized for slip detection [288]—future flexible sensing systems must rationally configure the types, structures, spatial layouts, and densities of sensors tailored to specific application scenarios. This strategic configuration will achieve an optimal design balance among environmental perception, precise manipulation, and rapid response. These trends impose unprecedentedly stringent requirements on structural design of flexible sensors. Beyond guaranteeing the superior performance of individual devices, it is imperative to ensure the high-fidelity transmission of electromechanical signals under complex operational conditions and to physically suppress signal crosstalk across multiple channels. Meeting these criteria is essential to establish a robust physical foundation for efficient back-end information processing [37].

*System-Level Convergence of “Sensing-Actuation” Closed Loops*. When high-performance flexible sensing devices are deeply integrated into equipment and continuously provide multidimensional environmental and proprioceptive information including force, deformation, temperature, and chemical signals [289], the technological vision of building fully functional closed-loop systems becomes increasingly clear. The edge-computing units of these sensing systems will be integrated into the intelligent core of the equipment, deeply combined with components such as actuators, and dynamically adjust the output based on feedback signals, thereby establishing low-latency closed-loop pathways of “sensing–processing–feedback–actuation” [21, 290] and fundamentally improving the autonomy, precision, and environmental adaptability of the equipment [21]. When sensors, edge-computing units, and actuators are seamlessly integrated at the system level, the equipment will be able to interact with the physical world with the sensitivity and insight of natural systems, ultimately giving rise to human–machine integrated intelligent systems with genuine environmental adaptability [288], paving the way for cutting-edge applications such as soft surgical robots, neuro-prosthetics.

*Computing’s Migration to the Edge, Culminating In-Sensor.* The migration of computing power toward the network edge in flexible sensing systems is becoming a primary catalyst driving the implementation of the aforementioned closed-loop intelligence. With the maturity of advanced manufacturing technologies such as FHE, the computational power of intelligent sensing systems is accelerating its migration from centralized hubs—such as PCs, smartphones, and the cloud—toward the edge. In addition to flex-on-chip design, as illustrated in Fig. 14a, in-sensor computing and near-sensor computing are attracting growing research attention, enabling the preliminary processing of data from sensor devices and even the entire closed-loop system at the source, which greatly reduces the data transmission burden and response latency between the cloud or processing modules [253, 254]. Meanwhile, dedicated computing mechanisms and hardware architectures tailored for such integrated sensing-processing systems are emerging [291], providing new technical pathways for realizing low-power, high-efficiency edge intelligence. When these edge-computing units achieve seamless coordination with sensing, actuation, and other devices at the system level, flexible sensors will truly acquire the capability to leap from passive collection to active cognition and actuation.

*Flexible Energy Storage and Its Integration with Sensing Systems*. Stretchable, high-energy–density, and highly safe flexible energy storage devices will be widely applied in the field of flexible sensors, serving as the core driving force to promote the next generation of wearable systems toward true skin-conformal integration and all-weather deployment. Over the past decade, the performance of stretchable batteries and supercapacitors has been rapidly catching up with that of rigid devices. Through diversified design strategies such as wave structures, rigid islands, and intrinsically stretchable materials, researchers have been able to maintain stable electrochemical output under mechanical deformation, and gradually overcome the inherent trade-off between energy density, cycling durability, and packaging reliability [248]. In the future, intrinsically stretchable systems with both isotropic stretchability and high active material content, as well as printable large-scale solutions compatible with commercial manufacturing processes, will become the key paths to achieve low-cost, high-safety, and long-lifespan flexible energy storage [292]. When these energy units can achieve system-level seamless integration with flexible sensors, control circuits, and wireless communication modules, truly meeting wearable-level requirements such as thinness, softness, conformality, and washability, flexible sensors will evolve from “sensing components” relying on external power supply to fully independent “intelligent sensing nodes”, releasing their full application potential in scenarios such as healthcare, human–computer interaction, and motion monitoring.

*Advanced Manufacturing for Sensor Fabrication Demands.* Facing the above advancements, miniaturized and reliable manufacturing of sensors with sophisticated architectures and electrodes is increasingly needed. Accordingly, advanced manufacturing techniques and stringent fabrication processes that can monolithically produce complex structures [293, 294] and that can produce delicate patterning of high-density device arrays [84] are expected to be widely used, such as 3D printing and high-precision lithography.

*Integration of Large Language Models (LLMs) into Cognitive Sensing.* Neural networks discussed in this review primarily perform initial processing and primary cognition of sensor data. On the opposite, LLMs allow the systems to undertake higher-level cognitive functions, including contextual understanding, multimodal information fusion, and complex decision-making, so as to facilitate their effective integration into embodied intelligence systems. Therefore, LLMs critically function across the core operational stages, spanning perception, planning, decision-making, and motion control [295]. This will also promote the development of the closed-loop system. However, this enhanced capability also demands more on the communication infrastructure and edge-computing resources.

*Deepening Interdisciplinary Collaboration.* In the context of fast progress in the field of flexible sensors, increasing close cooperation and convergence across multiple disciplines are needed, which include mechanical engineering, electronic information, materials science, chemistry, mathematics, and computer science [296].

*Balance between Technological Advancement and Ethical Principles.* Specifically, with flexible sensors now it is increasingly easy to get, transmit, and process biometric and privacy-sensitive data, such as fingerprints, voiceprints, bodily fluids, and health status. Therefore, ethically, we should carefully define the boundaries of the development and application of the technological innovation. We should clearly understand the associated constraints and safeguards, so as to ensure that technological progress remains aligned with ethical standards and fulfills societal responsibilities.

Despite the promising prospects, several critical bottlenecks must be overcome to realize the long-term goals:

*Lack of Standardization and Metrology Systems*. Unlike mature and widely deployed conventional rigid electronic devices, the development of flexible devices lacks unified research frameworks and technical standards for performance metrics, testing, and calibration. Specifically for the highly coupled co-design paradigm, the absence of a standardized cross-disciplinary metrology makes it currently challenging to construct a unified quantitative evaluation frameworks capable of dynamically assessing nonlinear synergistic trade-offs, systemic robustness, and boundary expansions. Consequently, rather than reducing complex system behavior to a simplistic numerical comparison against single-point optimizations, the current validation of co-design frameworks remains constrained to qualitative logic and representative system-level cases. Establishing multidimensional, system-level evaluation benchmarks—shifting the focus from single-parameter peak values to holistic structural reliability and environmental adaptability—will be a critical future step to translate academic breakthroughs into industrial quality assurance [286].

*Manufacturability and Scalability*. Achieving high‑yield, low‑cost, and reliable fabrication of devices composed of multiple materials, with complex three‑dimensional structures and high‑density interconnection requirements, on flexible and irregularly shaped platforms remains a severe economic and engineering challenge.

*Demand for High‑Energy‑Efficiency Edge Computing*. High‑efficiency edge computing is still essential for the development of sensors requiring rapid response. The visions of in‑sensor computing and near‑sensor computing remain long‑term goals. The FHE technology solutions must achieve a fundamental breakthrough to meet the urgent need.

*Insufficient Reliable Testing for Long‑Term Device Stability*. Although the research community has increasingly focused on the repeatability and stability of devices, studies are still limited by laboratory‑scale conditions. Rigorous testing of long‑term stability under harsh environments such as humidity, temperature, and corrosion remains restricted.

*Credibility Issues in Machine Learning*. The deep integration of machine-learning models in flexible sensors is an inevitable trend, but their “black‑box” nature introduces risks in safety‑critical applications including biomedicine, healthcare, and industrial manufacturing. Machine-learning algorithms with high robustness, interpretability, and fairness will help expand the deployment scope of flexible sensors.

*Sustainability and Whole-Life-Cycle Responsibility*. To achieve large-scale popularization of flexible sensors, the issues of electronic waste and carbon footprint must be directly addressed [37]. The large-scale application of carbon-based materials, recyclable/reconfigurable materials, eco-friendly materials, biomass-based materials, as well as low-carbon processes such as additive manufacturing and solution processing should be encouraged. Life-cycle analysis should be an essential step in the evaluation of new materials and devices.

## Abbreviations

CNTs: Carbon nanotubes
CB: Carbon black
rGO: Reduced graphene oxide
GO: Graphene oxide
AgNW: Silver nanowire
AgNPs: Silver nanoparticles
PDMS: Polydimethylsiloxane
ER: Epoxy resin
IG: Iono-gel
WPU: Waterborne polyurethane
PVDF: Poly(vinylidene fluoride)
TPU: Thermoplastic polyurethane
PI: Polyimide
PET: Polyethylene terephthalate
SBS/SEBS: Styrenic block copolymers
Gly: Glycerol
PANI: Polyaniline
PZT: Lead zirconate titanate
AlN: Aluminum nitride
NiO: Nickel oxide
IGZO: Indium gallium zinc oxide
ICE: Ion-conducting elastomer
IoT: Internet of things
LOD: Limit of detection
t_res: Response time
t_rec: Recovery time
ECG: Electrocardiogram
EMG: Electromyogram
E-skin: Electronic skin
RF: Radio-frequency
SNR: Signal-to-noise ratio
PCB: Printed circuit board
FPC: Flexible printed circuit
LIB: Lithium-ion battery
SOH: State-of-health
FHE: Flexible hybrid electronics
I/O: Input/Output interfaces
SCC: Signal conditioning circuit
ADC: Analog-to-digital converter
CDC: Capacitance-to-digital converter
MCU: Microcontroller
VNA: Vector network analyzer
SPDT: Single-pole double-throw
MUX: Multiplexer
TDMA: Time‑division multiple access
Conv: Convolution layer
FC: Fully connected layer
SVM: Support vector machines
KNN: K-nearest neighbors
MLP: Multi-layer perceptron
CNN: Convolutional neural network
RNN: Recurrent neural network
QRNN: Quasi-recurrent neural network
LSTM: Long short-term memory network
FEM: Finite-element method
LLM: Large language model
AI4S: Artificial intelligence for science
CMS: Computational materials science
AI4MS: Artificial intelligence for materials science
